# Learning the properties of adaptive regions with functional data analysis

**DOI:** 10.1371/journal.pgen.1008896

**Published:** 2020-08-27

**Authors:** Mehreen R. Mughal, Hillary Koch, Jinguo Huang, Francesca Chiaromonte, Michael DeGiorgio

**Affiliations:** 1 Bioinformatics and Genomics at the Huck Institutes of the Life Sciences, Pennsylvania State University, University Park, Pennsylvania, United States of America; 2 Department of Statistics, Pennsylvania State University, University Park, Pennsylvania, United States of America; 3 Department of Computer and Electrical Engineering and Computer Science, Florida Atlantic University, Boca Raton, Florida, United States of America; University of Western Ontario, CANADA

## Abstract

Identifying regions of positive selection in genomic data remains a challenge in population genetics. Most current approaches rely on comparing values of summary statistics calculated in windows. We present an approach termed *SURFDAWave*, which translates measures of genetic diversity calculated in genomic windows to functional data. By transforming our discrete data points to be outputs of continuous functions defined over genomic space, we are able to learn the features of these functions that signify selection. This enables us to confidently identify complex modes of natural selection, including adaptive introgression. We are also able to predict important selection parameters that are responsible for shaping the inferred selection events. By applying our model to human population-genomic data, we recapitulate previously identified regions of selective sweeps, such as *OCA2* in Europeans, and predict that its beneficial mutation reached a frequency of 0.02 before it swept 1,802 generations ago, a time when humans were relatively new to Europe. In addition, we identify *BNC2* in Europeans as a target of adaptive introgression, and predict that it harbors a beneficial mutation that arose in an archaic human population that split from modern humans within the hypothesized modern human-Neanderthal divergence range.

## Author summary

As populations adapt to their environments, specific patterns indicating selection remain in the distribution of genetic diversity across their genomes. A hallmark of positive natural selection is the reduction of genetic diversity surrounding beneficial mutations. The origin of the beneficial mutation, or whether it originated in a population being examined or within another, can be uncovered through the spatial distribution of the reduction of genetic diversity. In addition, other information about the strength, timing, and initial frequency of beneficial mutations can be learned by examining patterns of diversity across genomic regions. We use functional data analysis to capture differences among the spatial distributions of genetic variation expected by diverse evolutionary processes, and further apply it to dissect how selection parameters affect such patterns. Using this method, we learn the underlying origins, timings, and strengths of beneficial mutations that have impacted modern human genomic diversity.

## Introduction

Positive selection is one the most fundamental forces shaping the diversity of life that we can observe today [1, 2]. When positive selection acts on a beneficial mutation, it causes a “wave-like” pattern in the decrease in diversity of the genome [3]. As in waves found in the ocean or air, certain patterns might emerge depending on the properties of the cause and the environmental materials (genetic background). Examining these patterns might allow us to learn about the forces causing them. For example, the angle between the crest (top of a wave) and trough (bottom of a wave) might be informative for learning about the strength of selection (and concurrently the time taken for a selective event to occur). Similarly, different modes of positive selection may have on average different patterns. For example, if the crest of the wave extends above the rest position (neutrality), then this may be the signal of adaptive introgression as shown in ref. [4].

Capturing diversity patterns as they vary spatially has been the goal of a number of recent methods [5, 6, 7]. References [5] and [6] attempt to recognize sweeps by learning how diversity (measured by summary statistics) changes across a number of windows encompassing the sweep. However, these methods do not explicitly model the overall patterns formed by selection events. Other methods forgo explicitly measuring diversity and transform SNP data directly to images to learn population-genetic parameters such as recombination rates [8, 7] and to identify selected regions [7]. The complementary approach shown in ref. [9] explicitly models the spatial autocorrelation of summary statistics to capture the underlying wave patterns produced by selective sweeps.

Fortunately, there exist techniques not widely applied in genomics that allow observations on continuous data [10]. Functional data analysis is a recent sub-field of statistics in which measured values are known to be the output of functions [11, 12]. Relatedness between data points is inherent in this type of data analysis, which operates on values across a continuum. Transforming our measures of genetic diversity across a genomic region into functional data ensures that the spatial pattern is used to draw conclusions. Although we will be applying this method to assess how genetic diversity varies across the space of a genomic region, there is potential to apply this method to understand how diversity changes temporally [*e.g*., 13, 14, 15]. With the deluge of ancient genome datasets emerging, it may be possible to examine how the spatial distribution of genetic diversity changes across time at different positively-selected genomic regions to learn their adaptive parameters, such as selection strength, sweep softness, and timing of selection. Functional data analysis can also be applied to understand how genetic diversity changes across physical geographic regions and can potentially be useful in ecological modeling [*e.g*., 16, 17, 18].

We present a method termed *SURFDAWave* (Sweep inference Using Regularized FDA with WAVElets) in which we first model genetic diversity as functions, and then learn the importance of different aspects of genetic diversity across the examined genomic space in predicting selection parameters. We show that *SURFDAWave* accurately predicts parameters such as selection strength, initial frequency of mutation before becoming beneficial, and time of selection. We also demonstrate that *SURFDAWave* can be used to classify selective sweeps, while remaining robust to confounding factors. Finally, we apply *SURFDAWave* to empirical data to predict the selection parameters on regions classified as sweeps.

## Results

**Fig 1:**
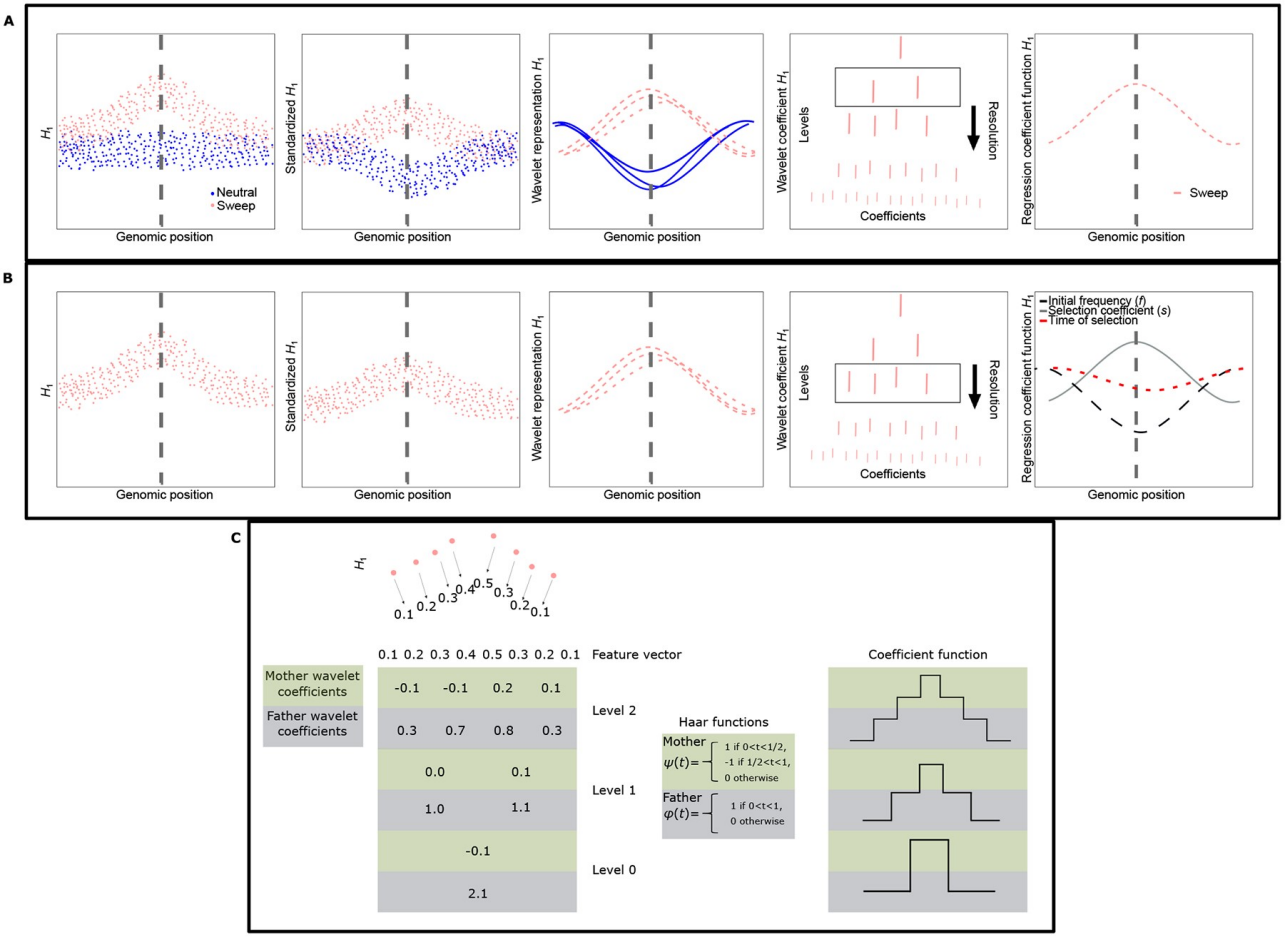
Cartoon illustrating *SURFDAWave* function. For each statistic, *SURFDAWave* standardizes values before transforming values into their wavelet representations (Middle boxes in panels A and B.). The wavelet representations are analyzed at all possible levels from the most detailed or highest level to least detailed or lowest level (Right of middle boxes in panels A and B). The top row shows how a binary classifier chooses wavelet coefficients to differentiate between sweeps and neutrality. Because the case shown is binomial, there is only one line showing the function for a sweep, as the function for neutrality would be the inverse. The middle row shows how there is a separate model for each selection parameter we predict. In this case the three different colored lines in the right box are the regression coefficient functions for three different selection parameters. (Panel C) A cartoon example of how a feature vector might undergo discrete wavelet transform. A feature vector (here of length eight) is transformed by either pairwise subtraction (for mother wavelet coefficients) or pairwise addition (for father wavelet coefficients) in subsequent steps to obtain a multiresolution breakdown of the data. Level zero provides the least amount of detail, while level two captures the feature vector values in higher resolution. *SURFDAWave* uses this breakdown of coefficients to identify important ones through penalized regression. Using wavelet functions, such as the Haar functions shown here it is then possible to generate wavelets (coefficient function) as shown in the final panel on the right.

*SURFDAWave* is a wavelet-based regression method used to classify selective sweeps and predict adaptive parameters (Fig 1; see Materials and methods for a brief discussion of wavelets). Here we briefly present its performance in terms of both classification of selective sweeps and in estimating parameters responsible for shaping sweeps. We compare classification performance of *SURFDAWave* to *Trendsetter* [9], as *Trendsetter* also models the spatial autocorrelation of summary statistics, and we also provide a comprehensive comparison to two other leading sweep classifiers—evolBoosting [19] and diploS/HIC [6]. See Materials and methods for details on these comparisons, as well as important considerations regarding the alteration of default settings of *Trendsetter* to use the same summary statistics as *SURFDAwave* and the use of two classes for diploS/HIC instead of the five classes that it was originally designed for. Although we modify *Trendsetter* from its original implementation, we chose to focus on how the modeling of the summary statistics, rather than the number or choice of summary statistics, would affect differences in classification rates between these two methods.

### Classification of selective sweeps

We trained the *SURFDAWave* classifier to differentiate between sweeps and neutrality as described in *Materials and Methods*. We conducted simulations under three different demographic histories—constant size, human sub-Saharan African (YRI), and human European (CEU)—to compare how different demographic histories affect our results [20]. All simulations are conducted using SLiM [21] using a mutation rate of 1.25 × 10^−8^ per site per generation [22] and recombination rate drawn from an exponential distribution with mean 3 × 10^−9^ per site per generation (truncated at three times the mean) to simulate two Mb regions (see Materials and methods). For all sweep simulations, we drew selection start time, initial frequency of beneficial mutation, and selection strength of the beneficial allele from a distribution, such that all sweep scenarios comprise a range of hard and soft sweep settings. The initial frequency of the beneficial allele and the selection coefficient are drawn from *f* ∈ [1/(2*N*), 0.1] and *s* ∈ [0.005, 0.5] per generation, respectively, while the start time of the mutation was drawn uniformly at random from between 1,020 and 3,000 generations ago. Though it is possible to apply *SURFDAWave* with many combinations of summary statistics in any number *p* = 2^*J*^ windows (where *J* is a positive integer), we use an implementation that employs the summary statistics $\hat{\pi }$, *H*_1_, *H*_12_, *H*_2_/*H*_1_, and frequencies of first to fifth most common haplotypes, all calculated in *p* = 128 genomic windows (see Materials and methods). The limitation that *p* = 2^*J*^ is necessary for the process of discrete wavelet transform as used by *SURFDAWave* (see Materials and methods). The discrete wavelet transform allows data to be resolved into several levels, each containing information with differing amounts of detail. The number of levels is determined by *J*, and the process of resolving data into levels is the limiting factor for *p*, as at each level the number of wavelets is half of the previous. Limiting the number of windows to 2^*J*^ ensures that the number of wavelets is an integer at all levels (Fig 1).

**Fig 2:**
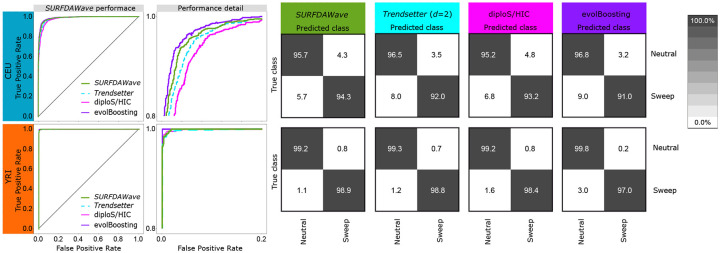
*SURFDAWave* classifier performance compared to *Trendsetter*, diploS/HIC, and evolBoosting when differentiating between sweeps and neutrality and trained and tested with simulations based on CEU (top row) and YRI (bottom row) demographic history. (Left) Power to differentiate between sweep and neutrality by comparing the probability of a sweep under sweep simulations with the same probability in simulations of neutrality including zoomed in region between 0.0 and 0.2 on the *x*-axis and 0.8 and 1.0 on the *y*-axis. (Right confusion matrices) Confusion matrices comparing classification rates of the methods. *SURFDAWave* applied using Daubechies’ least-Asymmetric wavelets to estimate spatial distributions of summary statistics with *γ* penalties and level chosen through cross validation (see *Training the models*). Summary statistics $\hat{\pi }$, *H*_1_, *H*_12_, *H*_2_/*H*_1_, and frequency of the first, second, third, fourth, and fifth most common haplotypes used by both *Trendsetter* and *SURFDAWave*.

**Fig 3:**
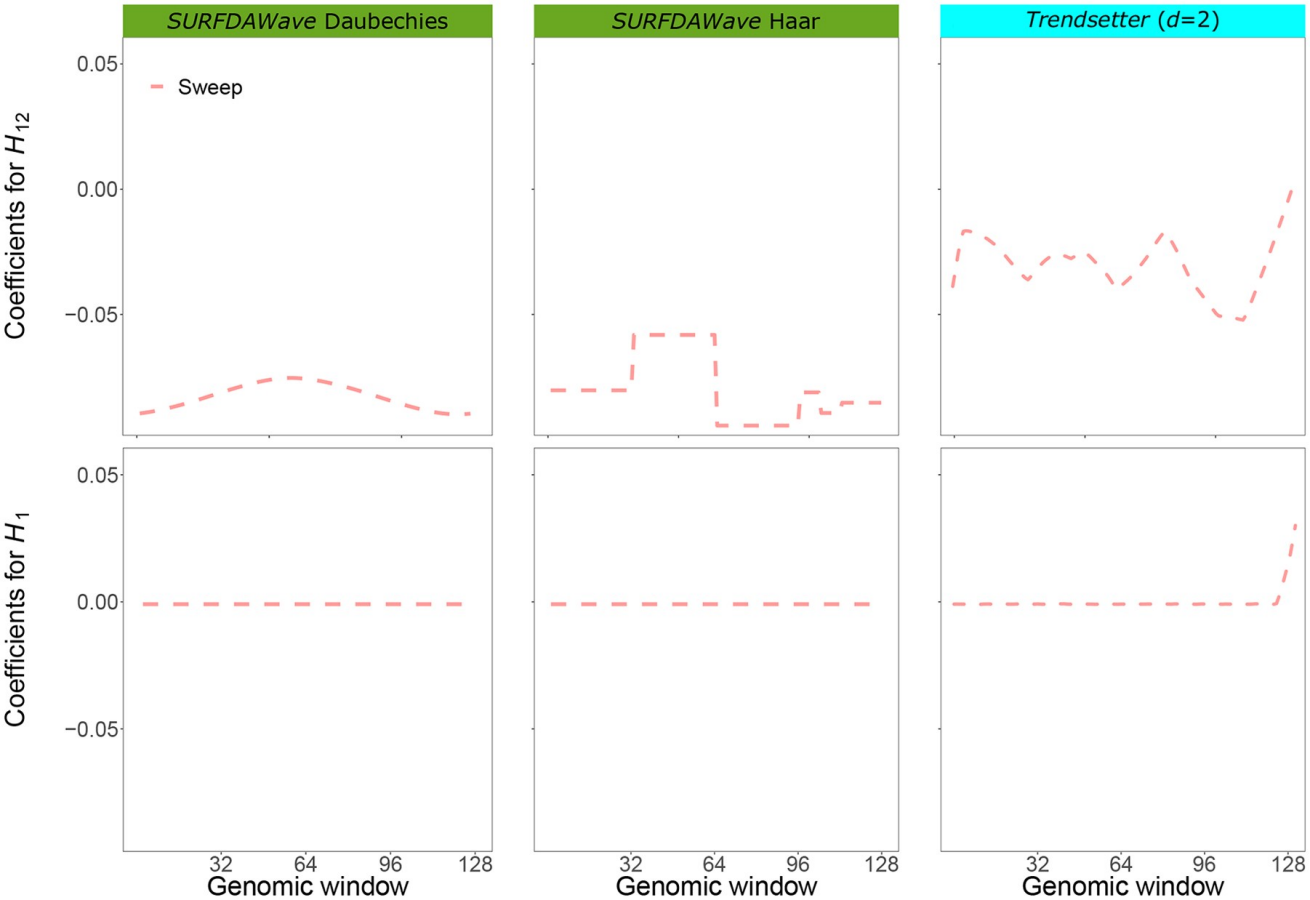
Reconstructed wavelets from regression coefficients (*β*s) in sweep versus neutrality scenarios for summary statistics *H*_1_ and *H*_12_ for *SURFDAWave* and *Trendsetter* when both methods were trained on simulations of scenarios simulated under demographic specifications for European CEU demographic history. Note that the wavelet reconstructions for all summary statistics are plotted on the same scale, thereby making the distributions of some summaries difficult to decipher as their magnitudes are relatively small. *SURFDAWave* results compare the use of Daubechies’ least-asymmetric to Haar wavelets to estimate spatial distributions of summary statistics. Summary statistics $\hat{\pi }$, *H*_1_, *H*_12_, *H*_2_/*H*_1_, and frequency of the first, second, third, fourth, and fifth most common haplotypes used by both *Trendsetter* and *SURFDAWave*. Level and *γ* chosen through cross validation for *SURFDAWave* (see *Training the models*).

We first train a classifier using summary statistics calculated on simulations that reflect the CEU European human demographic history [20]. Fig 2 and S3 Fig show that *SURFDAWave* has similar accuracy to *Trendsetter* regardless of the regularization penalty used [23]. This is reflected in the patterns we observe for importance of summary statistics through examining the regression coefficients (*β*s) for each model. Fig 3 shows how *SURFDAWave* and *Trendsetter* both identify *H*_1_ as uninformative, while *H*_12_ is informative. S1 and S2 Figs respectively provide information on how these two methods have similar patterns of importance for other summary statistics as well. In addition, comparison to diploS/HIC and evolBoosting show that these methods perform comparably to *SURFDAWave*, with evolBoosting classifying neutral simulations correctly more often than all other methods, but performing worse overall. However, classification by diploS/HIC differed from *SURFDAWave* only by a few percentage points.

To examine whether the type of wavelet used influences *SURFDAWave*’s classification rates, we incorporate a comparison of two popular wavelets (Daubechies’ least-asymmetric vs. Haar). The Haar wavelets are composed of block shaped functions, while Daubechies’ least-asymmetric wavelets are composed of more localized smooth functions (Fig 3). Because the shapes of the spatial distributions of genetic diversity are relatively simple, we anticipate both of these types of wavelets to be able to adequately capture the signal. This expectation is also motivated by results in ref. [9] comparing the classification accuracy of *Trendsetter* when using constant and linear trend-filtering functions, which respectively model curves with similar characteristics to the Haar and Dabechies’ least-asymmetric wavelets employed by *SURFDAWave*. We find that the type of wavelets used as basis functions does not dramatically influence the overall classification rates (S3 Fig). However, visualizing the coefficient functions for each summary statistic shows that the overall shape is much smoother when using Daubechies’ least-asymmetric (S1 Fig) compared to Haar (S4 Fig) wavelets. We find smoothness of the coefficient functions to be desirable, and for this reason, most of our results are shown using Daubechies’ least-asymmetric wavelets. We also notice that although classification rates are similar regardless of whether we use ridge penalization (*γ* = 0), lasso penalization (*γ* = 1), or choosing the optimal elastic net parameter *γ* through cross validation (S3 Fig), the resulting regression coefficient functions are vastly different, especially when we use *γ* = 0 (S1, S5 and S6 Figs).

We also train a classifier to differentiate between selective sweeps and neutrality using simulations of the YRI sub-Saharan African human demographic history [20] over a range of *γ* values in *SURFDAWave* and all compared classification methods. Overall, we notice an increase in the percentage of simulations classified correctly when we compare to classifiers trained under the CEU demographic history for all of the methods tested (Fig 2 and S7 Fig). Noticeably, evolBooting again outperforms all other methods in correct classification of neutrality, but still has smaller overall classification accuracy than the other methods. Comparing within *SURFDAWave*, we see that the patterns formed by the spatial distributions of the coefficients for each summary statistic are similar regardless of the *γ* penalty used (S8–S10 Figs). The noisy functions resulting from the use of *γ* = 0 tend to obscure any pattern in the spatial distribution of the underlying regression functions and as a result make the function more difficult to interpret. For this reason we proceed with either *γ* = 1 or *γ* chosen through cross validation.

Through cross validation (see *Training the models*) we also chose the level at which the discrete wavelet transform (DWT) has best performance for classification. Using wavelets as our basis functions has the advantage of allowing our regression coefficients to be represented at different resolutions, denoted by different levels *j*_0_ (see Materials and methods). Choosing these levels through cross validation allows our method to determine the smoothness of the regression coefficient function because choosing a coarser resolution (lower level) results in a smooth function, whereas choosing a finer resolution (higher level) will result in a more rugged function. As detailed in *Materials and Methods*, the total number of levels at which DWT can be applied equals log_2_(*p*) − 1, which when *p* = 128 (as is used here) means we have six different levels *j*_0_ ∈ {0, 1, 2, 3, 4, 5}. To illustrate the differences among levels, we show a model using DWT with the coarsest level (*j*_0_ = 0) compared to a model using DWT with the finest (*j*_0_ = 5), with both models employing Daubechies’ least asymmetric wavelets with a lasso (*γ* = 1) penalty (S11 Fig). It is clear that the summary statistic *H*_12_ is informative for both of these models, however the noisy wavelet reconstructions seen for *j*_0_ = 5 reveals an emphasis on local features that is absent when we enforce *j*_0_ = 0.

To compare the effect of bottlenecks and expansions on classification rates to those under a constant-size demographic history, we trained and tested a classifier using simulations of a constant-size demographic model to differentiate between neutrality and sweeps. As expected, we find both neutral and sweep simulations are classified correctly more often than when classifying simulations of more complicated non-equilibrium demographic histories (S3 Fig), such as those of the CEU and YRI populations.

**Fig 4:**
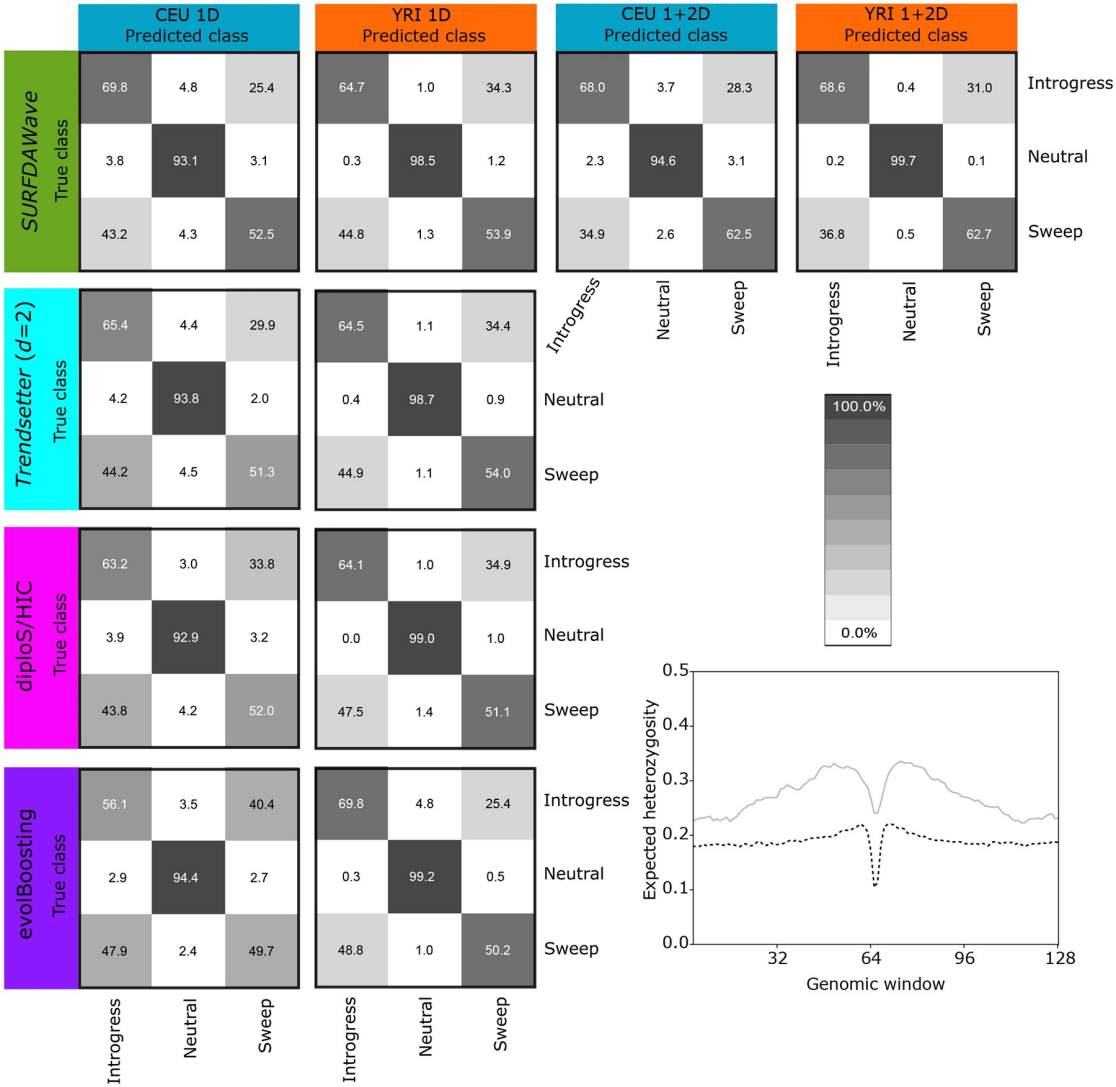
Classification rates when differentiating among adaptive introgression, sweeps, and neutrality. (Top row) *SURFDAWave* classification rates. The two first columns are showing classification rates when trained and tested with simulations conducted under CEU European and YRI Yoruban population demographic history specifications when trained with one-dimensional statistics ($\hat{\pi }$, *H*_1_, *H*_12_, *H*_2_/*H*_1_, and frequencies of first to fifth most common haplotypes) while the second two columns show results for the same two populations using both one and two-dimensional statistics (including the preceding statistics as well as the mean, varaiance, skewness, and kurtosis of *r*^2^). The *γ* for the classifiers trained with only the one dimensional statistics is chosen through cross validation (see *Training the models*), but is specified *γ* = 1 for the results in the two columns on the right. The level is chosen through cross validation for all *SURFDAWave* models. (Bottom three rows of confusion matrices) Confusion matrices comparing classification rates for *Trendsetter*, diploS/HIC, and evolBoosting with simulations conducted under European (CEU) and Yoruba (YRI) demographic history specifications. Summary statistics $\hat{\pi }$, *H*_1_, *H*_12_, *H*_2_/*H*_1_, and frequency of the first, second, third, fourth, and fifth most common haplotypes used by *Trendsetter*. (Bottom right) Value of expected heterozygosity across simulated regions of adaptive introgression with varying divergence times. The black dotted line shows the value of the statistic when the divergence time is shorter (30,000 generations ago) and the gray line shows the value when the divergence time is longer (400,000 generations ago).

Adaptive introgression is a complex form of natural selection that produces genetic diversity footprints distinct from typical selective sweeps [4]. In both selective sweeps and adaptive introgression, diversity generally decreases surrounding the beneficial mutation. In adaptive introgression, however, diversity increases before the signal decays to the level of neutrality. This slight increase in diversity compared to the neutral background is most clearly seen when the two populations (donor and recipient) are highly diverged (Fig 4). We test how well *SURFDAWave* can differentiate among adaptive introgression, sweeps, and neutrality, using the same summary statistics discussed in the *Results* section (see Material and methods for simulation details). Similar to previous sweep simulations, for adaptive introgression simulations we drew selection start time, initial frequency of beneficial mutation, selection strength of the beneficial allele, and the donor and recipient divergence time from a distribution, such that all adaptive introgression scenarios comprise a range of hard and soft sweep settings. As shown in Fig 4, *SURFDAWave* is only able to correctly classify sweep simulations in 52.5% of cases, misidentifying them as adaptive introgression 43.2% of the time under the CEU-based simulations with similar results for YRI. As we saw in Fig 4, this is because when divergence times for donor and recipient populations are recent, then the signature of adaptive introgression looks more like a selective sweep. We also compare *SURFDAWave* to the classifiers evolBoosting, diplo/SHIC, and *Trendsetter* and see that classification results from other methods are similar to *SURFDAWave* (Fig 4), with correct classification ability decreasing significantly when compared to the two class problem of distinguishing between sweeps and neutrality. Overall, we find that evolBoosting performs better than other methods when differentiating neutrality from selection, but slightly worse in the classification of sweeps. We also note that all methods seem to perform more similarly to each other when trained and tested with the YRI demographic history.

**Fig 5:**
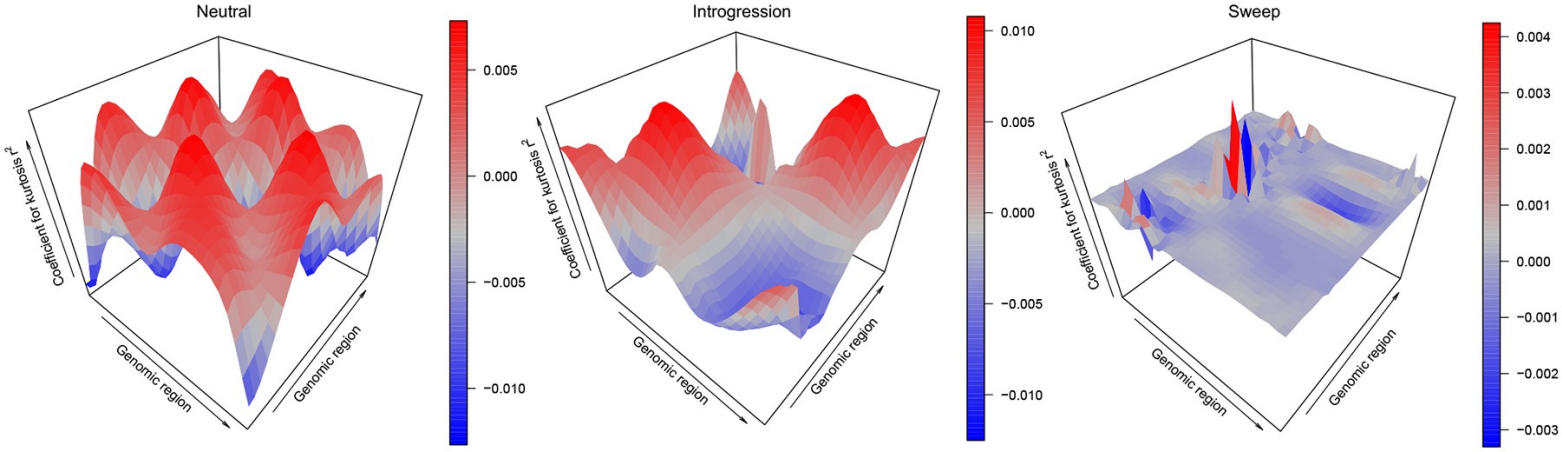
Three dimensional representations of reconstructed wavelets from regression coefficients (*β*s) when differentiating among adaptive introgression, sweeps, and neutrality for summary statistics kurtosis of pairwise *r*^2^ for *SURFDAWave* when *γ* = 1, when trained with statistics $\hat{\pi }$, *H*_1_, *H*_12_, *H*_2_/*H*_1_, frequencies of first to fifth most common haplotypes, and mean, variance, skewness, and kurtosis of pairwise *r*^2^. *SURFDAWave* was trained on simulations of scenarios simulated under demographic specifications for European CEU demographic history. Note that the wavelet reconstructions for all summary statistics are plotted on the same scale, thereby making the distributions of some summaries difficult to decipher as their magnitudes are relatively small. *SURFDAWave* results shown are using Daubechies’ least-asymmetric wavelets to estimate spatial distributions of summary statistics. Level chosen through cross validation.

To investigate whether the inclusion of other summary statistics, which may better assess genomic variation, boosts classification accuracy of *SURFDAWave* we include an additional set of summary statistics, specifically adding the mean, variance, skewness, and kurtosis of the squared correlation coefficient *r*^2^ [24] calculated between all possible SNPs sampled from each pair of windows (see Materials and methods). Because visualizing these statistics in square matrices is informative, we refer to them as two-dimensional statistics, and refer to $\hat{\pi }$, *H*_1_, *H*_12_, *H*_2_/*H*_1_, and frequencies of first to fifth most common haplotypes as one-dimensional statistics. We see that the inclusion of two-dimensional statistics increases the correct classification of selective sweeps substantially for both populations to 62.5% in CEU and 62.7% in YRI (Fig 4). The percent of adaptive introgression simulations classified correctly also increased for YRI going from 64.7% to 68.6%. With the addition of two-dimensional statistics we find that *SURFDAWave* has the most significant increase in correct classification rates, compared to all other methods. We can see how the inclusion of the two dimensional statistics affects the model by directly comparing the reconstructed wavelets across the spatial distributions of the nine summary statistics included in both models. By examining the coefficients of the two-dimensional statistics for the model using both types of statistics, we can see that the skewness and kurtosis of *r*^2^ are informative in separating neutrality from the other classes (Fig 5 and S12 Fig). Interestingly, the statistic *H*_1_ is important in separating neutrality from both types of selection in the model including two-dimensional statistics for the CEU demographic history, but clearly does not serve this purpose in the model trained with only one-dimensional statistics (S13 and S14 Figs). However, this is not the case when examining the same statistic for YRI demographic history (S15 and S16 Figs). This may be due to the fact that when different statistics are included the importance of other statistics in the model is changed.

### Classification with confounding factors

**Fig 6:**
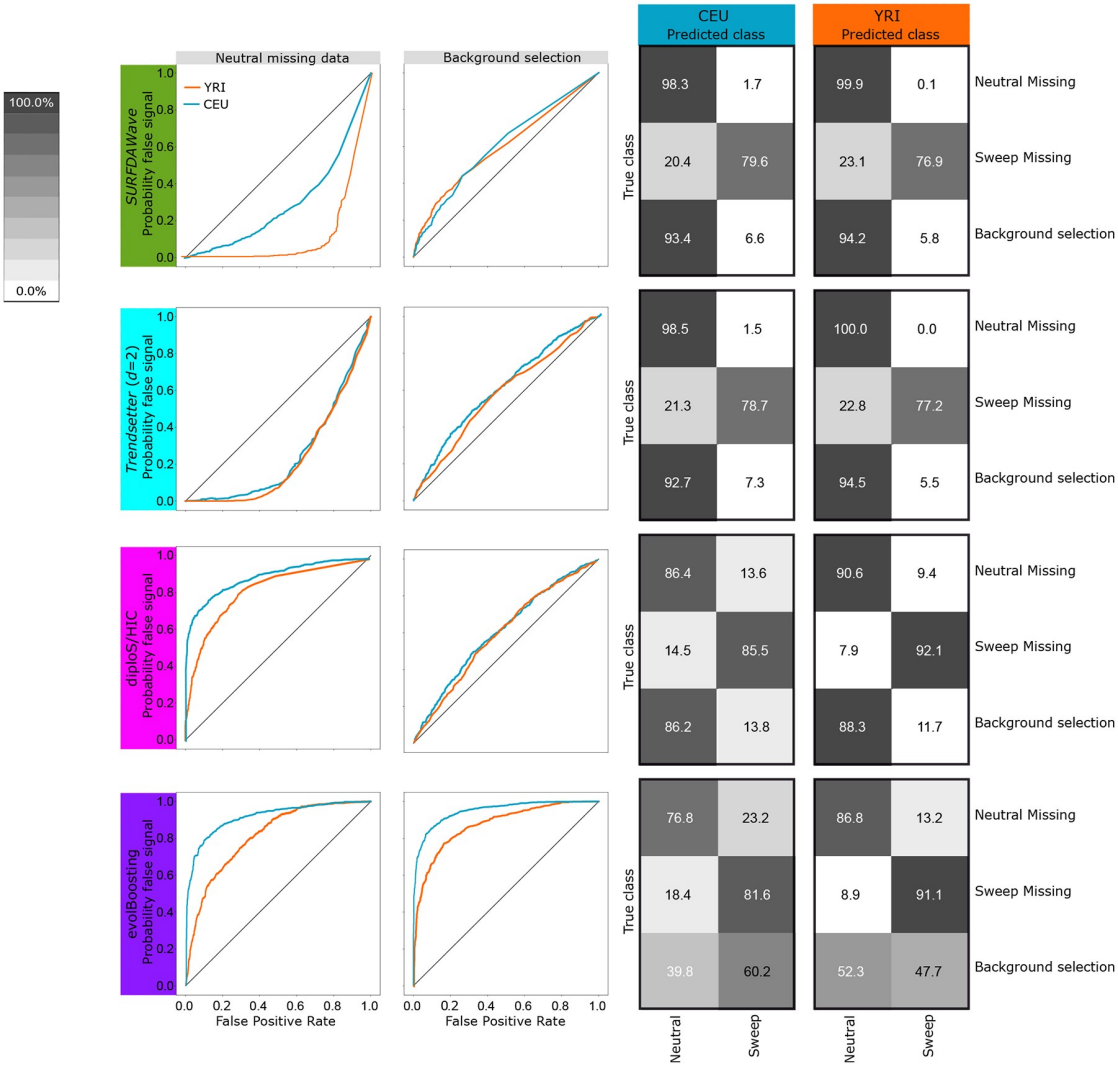
Comparison of method performance under confounding factors (missing data and background selection) when trained with either CEU or YRI demographic histories when *SURFDAWave*, *Trendsetter*, diploS/HIC, and evolBoosting are trained to differentiate between sweeps and neutrality. (Left column) Probability of mis-classifying neutrally-evolving genomic regions missing data as a sweep. Comparing probability of sweep in simulations missing data (probability of false signal) with the probability of any sweep in neutral simulations (false positive rate). (Left middle column) Probability of mis-classifying background selection simulations as sweep. Comparing the probability of a sweep in simulations of background selection (probability of false signal) with probability of sweep in neutral simulations (false positive rate). (Right columns) Confusion matrices showing classification rates when classifying simulations of each class with missing data, and when classifying background selection simulations. Results for both CEU (Right middle column) and YRI (Right column) demographic history. *SURFDAWave* is trained using Daubechies’ least-Asymmetric wavelets. Optimal *γ* and and level were chosen through cross validation (see *Training the models*). Summary statistics $\hat{\pi }$, *H*_1_, *H*_12_, *H*_2_/*H*_1_, and frequency of the first, second, third, fourth, and fifth most common haplotypes used by both *Trendsetter* and *SURFDAWave*.

Testing *SURFDAWave* on simulations of biological events that might confound classification is necessary to ensure that it can be applied under diverse empirical scenarios. For this reason we test classification performance of *SURFDAWave* under simulations with extensive missing data. To simulate missing data as we might find it in genome sequences due to technical issues, such as alignability and mappability [25], we remove large randomly spaced chunks of the simulated data (see Materials and methods). We show that missing data does not substantially affect the performance of *SURFDAWave* when classifying neutral simulations with missing data (Fig 6). We do, however, observe a slight decrease in performance in the classification of selective sweeps when sweep simulations are missing data, with an increase in the percentage of sweep simulations missing data being classified as neutral. This robustness to missing data can be attributed to the types of summary statistics applied and the manner in which they are calculated using SNP-delimited windows [9]. Another common confounding factor is background selection, in which deleterious mutations cause a loss of diversity which might be confused for selection signatures [26]. For this reason we test *SURFDAWave*’s performance on background selection, which we simulate based on the distribution of effect sizes and spatial distribution of coding elements in the human genome, as in refs. [27] and [9] (see Materials and methods for details). We find that 93.4% and 94.2% of background selection scenarios under the CEU and YRI demographic histories, respectively, are classified as neutral (Fig 6). In comparison to other classifiers we notice the performance of *SURFDAWave* is comparable to *Trendsetter* and diploS/HIC under these background selection scenarios, but that evolBoosting erroneously classifies background selection as a sweep often (Fig 6). We also notice that while both *Trendsetter* and *SURFDAWave* tend to conservatively misclassify sweep simulations missing data as neutral, evolBoosting and diploS/HIC tend to misclassify neutral simulations missing data as sweeps. The reason for this elevated rate of misclassifying neutral regions missing data as sweeps is because evolBoosting and diploS/HIC use as input summary statistics computed in fixed physical length windows by default, meaning that reductions in haplotypic diversity due to missing data can masquerade as false sweep signatures. However, ref. [9] demonstrated that these issues can be avoided by ensuring that evolBoosting and diploS/HIC are trained with simulations containing missing data, and so we believe missing data would not be a major issue for any of the classifiers that we examine.

Along with issues of background selection and missing data, there are also known difficulties with establishing accurate demographic histories of present populations. Similar to the results shown in ref. [9], we again show that *SURFDAWave* loses performance when demographic specifications are less accurate. With a classifier trained to differentiate between sweeps and neutrality in CEU European populations, we mis-classify 37.9% of sweep simulations conducted under YRI sub-Saharan African demographic history as neutral. However, the percentage of neutral YRI simulations classified as neutral increases to 99.1% when tested with a CEU trained demographic history. In the opposite case, with the classifier trained to differentiate sweeps from neutrality with simulations of YRI, when we test sweep simulations conducted under the CEU demographic history we classify 99.2% correctly, however we mis-classify simulations of neutrality as sweeps 26.0% of the time (S18 Fig). These mis-classifications are largely rescued when we train a classifier trained across a diverse set of demographic histories (S18 Fig), with classification rates for CEU almost reaching classification rates of when only trained with simulations conducted under the CEU demographic history and a slight decrease in correct percentages for the YRI history.

We next probe the effect of sample size on classification rates in simulated data (S19 Fig). In many cases, large sample sizes may be unavailable such as in the case of rare species, like bonobos, chimpanzees, and other great apes [28], and for this reason testing *SURFDAWave* on a variety of sample sizes (*n* = 20, 50, or 200) beyond the *n* = 100 already considered allows us to evaluate whether it still has power to distinguish sweeps from neutrality with more uncertainty in estimates of summary statistics. We observe a slight decrease in classification ability of *SURFDAWave* with a lower sample size of *n* = 20, but it is still able to classify greater than 90% of sweeps correctly. Similarly, *Trendsetter*, diploS/HIC, and evolBoosting also have noticeable decreases in classification rates when summary statistics are calculated from smaller samples. For all methods, the greatest increase in correct classification rate tends to stem from a sample size increase from *n* = 20 to *n* = 50.

Although we have designed *SURFDAWave* to be used to detect and understand selection in human populations, we believe its application can be extended to other species. To test this we apply the *SURFDAWave* classifier using simulations conducted under *Drosophila* parameters to differentiate between sweeps and neutrality (S20 Fig), and include a comparison to *Trendsetter*, diploS/HIC, and evolBoosting. Demographic parameters were based on the model of ref. [29], and we detail the procedure for simulating training and testing data under this model in the *Materials and Methods* section. In a similar pattern to human parameters, we see that neutrality is classified correctly more often by all methods than selection, and the overall correct classification percentages are lower for *Drosophila* parameters. However we note that all methods tend to be more conservative when classifying sweeps, often misclassifying sweeps as neutrality. This is because our simulations of *Drosophila* are conducted by drawing demographic parameters from posterior distributions of their estimates [29]. Uncertainty in this distribution make sweeps more difficult to detect as shown by refs. [30] and [31].

Finally, we test *SURFDAWave* to see how it performs under varying recombination rates. Training and testing with simulations using recombination rate drawn from an exponential distribution with mean 10^−8^ per site per generation shows results similar to our previous models (S21 Fig). Results from a model trained and tested with recombination rates drawn randomly from the CEU human recombination map show classification rates for neutrality that are similar to classification rates from the model using rates drawn from an exponential distribution with mean 3 × 10^−9^ per site per generation, but with lower percentages of sweep simulations classified correctly.

### Prediction of selection parameters

**Fig 7:**
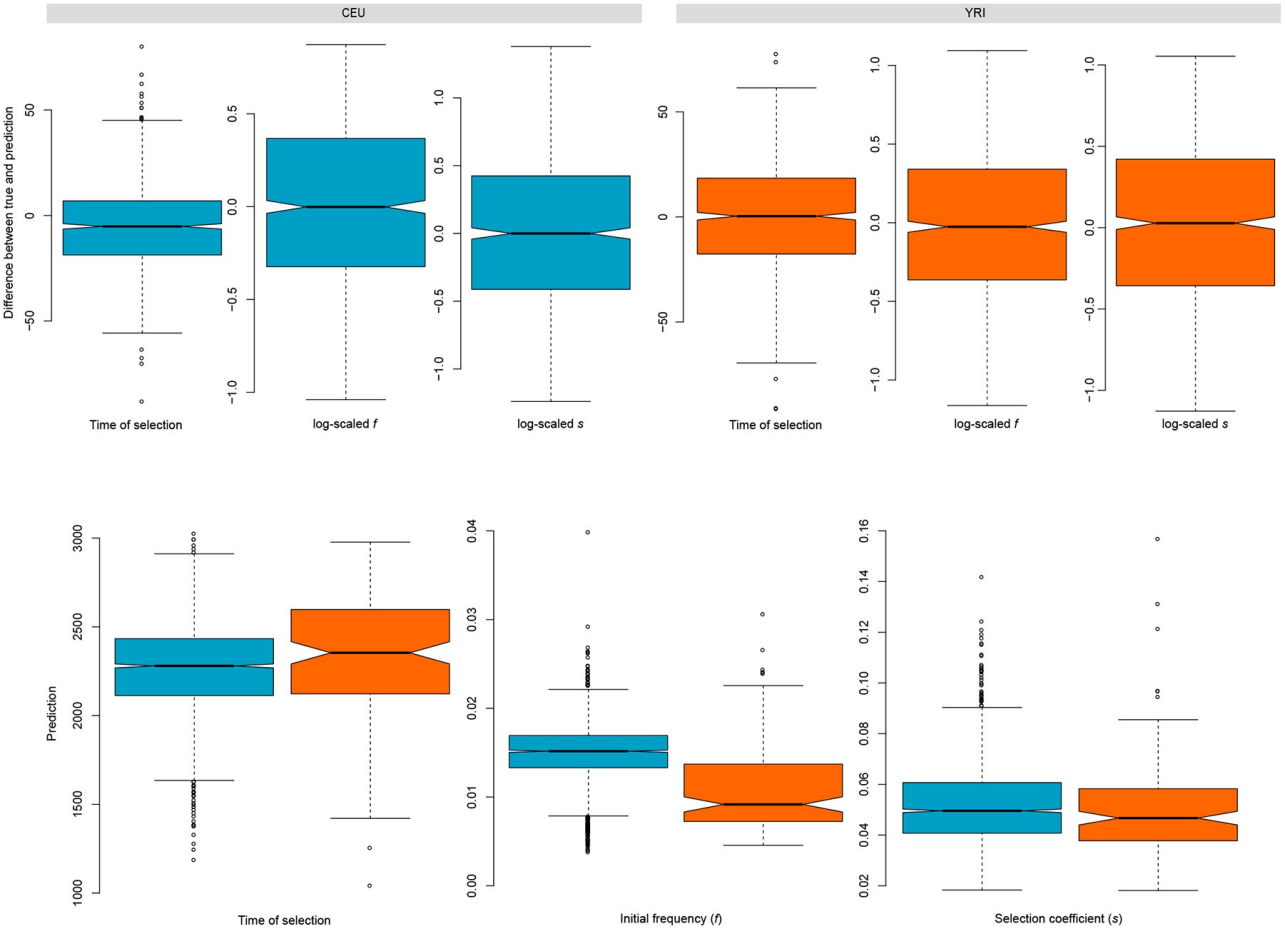
*SURFDAWave* predictors’ performance on simulated data and prediction results on empirical data. (Top row) Difference between unstandardized selection parameters with *SURFDAWave* for the CEU and YRI demographic models. (Left box plot) Difference in prediction and truth of log scaled time at which mutation became beneficial (measured in generations before present). (Middle box plot) Difference in prediction and truth of log scaled frequency reached by mutation prior to it becoming beneficial (*f*). (Right box plot) Difference in prediction and truth of log scaled selection coefficient (*s*). (Bottom row) Predicted distribution of selection parameters for all genes in YRI and CEU with probability of being classified as sweep greater than 0.7. (Left) Distribution of predicted time of selection (measured in generations before present). (Middle) Distribution of predicted frequency reached by mutation before becoming beneficial (initial frequency (*f*)). (Right) Distribution of predicted selection coefficient (*s*).

Classification of selective sweeps provides a limited understanding of the evolutionary processes shaping genomic regions. To gain deeper insight about the underlying adaptive processes, we also tested the ability of *SURFDAWave* to predict the selection parameters involved in shaping sweeps. We trained a multi-response linear regression model to jointly learn the log-scaled initial frequency of the adaptive allele prior to it becoming beneficial, the log-scaled selection coefficient, and the time at which the mutation becomes beneficial (see Materials and methods) using demographic specifications for the CEU and YRI populations. We include the same set of *m* = 9 summary statistics as used to train the sweep classifier in the preceding section, each computed across *p* = 128 windows. Prediction of initial frequency, selection coefficient, and time of selection is accurate (S22 Fig) with the root mean squared error (RMSE) equal to 0.49 for the log-scaled selection coefficient, 0.43 for the log-scaled initial frequency, and 20.3 for time at which selection began for unstandardized log-scaled selection coefficient, unstandardized log-scaled initial frequency, and the unscaled and unstandardized time of selection, respectively (Fig 7). We find that the mean absolute error (MAE) is always lower in value than the RMSE (S1–S13 Tables). The RMSE for the YRI population is similar to that of the CEU (S1 and S2 Tables). Visualizing the coefficient functions after regularized regression conveys that most summary statistics are informative in predicting parameters (S23 and S24 Figs), with the exception of the frequency of the most common haplotype, which is flat across the entire spatial distribution in both models.

To test the influence of confounding factors such as missing data on the prediction model, we simulate missing data as in the *Classification with confounding factors* section above. We find that predicting parameters with missing data increases RMSE slightly (S3 and S4 Tables), with standardized RMSE for selection coefficient (*s*), initial frequency (*f*), and time of selection (*T*_sel_) changing from 0.91, 0.98, and 0.67 to 0.93, 1.03, and 0.87 in CEU and from 0.95, 0.96, and 0.76 to 1.12, 1.11, and 1.21 in YRI, respectively. This results in a percent change in the RMSE in CEU of 2.2% for *s*, 5.1% for *f*, and 29.8% for *T*_sel_. Similarly, for YRI we observe a percent change of 17.8%, 15.5%, and 59.2% for *s*, *f*, and *T*_sel_, respectively. We also test robustness of the *SURFADAWave* prediction model to demographic mis-specification, by considering test simulations performed under CEU demographic specifications with a model trained with simulations performed under YRI demographic specifications, and vice versa (S5 and S6 Tables). Again, we find that the RMSE increases compared to training and testing with the same population demographic histories for both experiments, but the RMSE is less than the error due to missing data with a percent change in *s*, *f*, and *T*_sel_ for CEU of 3.2, 8.1, and 32.1, respectively. For YRI, we find respective percent changes of 4.1, 22.9 and 47.4. In order to test whether it is possible to rescue this decrease in predictive ability because of demographic mis-specification, we train a model with a mixture of CEU and YRI simulations (S7 Table and S25 Fig). We notice that for most selection parameters our ability to predict is better than when we mis-specify demography.

We also simulate selective sweeps including background selection, simulated as described in *Classification with confounding factors*, with the exception of including a beneficial mutation in the center of the simulated chromosome. We find that the RMSE values are very close to the RMSE with no confounding factors (S8 and S9 Tables). Using simulations of differing sample sizes we test whether the number *n* of haploid genomes sampled influences our ability to predict selection parameters (S10 Table and S25 Fig). We show that there is clearly a decrease in error as sample size increases. We notice that the selection coefficient *s* has a more significant decrease in RMSE between sample sizes of *n* = 20 and 50 than between 50 and 200, whereas *f* experiences the opposite. In addition, we test two models with differing recombination rates to see how this type of variation affects our predictions. We find that using a recombination rate drawn from exponential distribution with mean 10^−8^ per site per generation truncated at three times the mean has similar results to using recombination rate drawn from exponential distribution with mean 3 × 10^−9^ per site per generation truncated at three times the mean (S11 Table and S25 Fig). This is likely because there is substantial overlap between these distributions. However, using varying recombination rate across simulated genomic segments drawn from a human empirical recombination map decreases our ability to predict selection parameters (S11 Table and S25 Fig). We notice our ability to predict all these selection parameters, especially *f* decreases. Specifically, our ability to predict *f* decreases by 6%. Finally, we test how selection parameter prediction would be affected if we tested with parameters that are not included in the training parameter range (S12 Table and S25 Fig). We test models trained under *f* ∈ [1/(2*N*), 0.1] with test simulations for which *f* ∈ [0.1, 0.2], such that starting allele frequency for sweeps is completely outside the distribution of the training data. Under this setting, we see that not only is the error for *f* inflated substantially, but for the other selection parameters as well.

In addition to being able to predict the selection parameters responsible for shaping classical selective sweeps, we also probed whether *SURFDAWave* could predict selection parameters important in shaping sweeps due to adaptive introgression. An interesting parameter specific to adaptive introgression is the time at which the donor and recipient populations diverged. Instead of predicting the time at which a mutation became beneficial, as we show above in *Prediction of selection parameter*, we train models to predict the donor-recipient split time, along with the selection strength and initial frequency of the mutation before it became beneficial (S13 Table and S26 Fig). The RMSE values for the selection strength and time of selection are similar to the values predicted for regular selective sweeps (S1 Table).

### Application to empirical data

**Fig 8:**
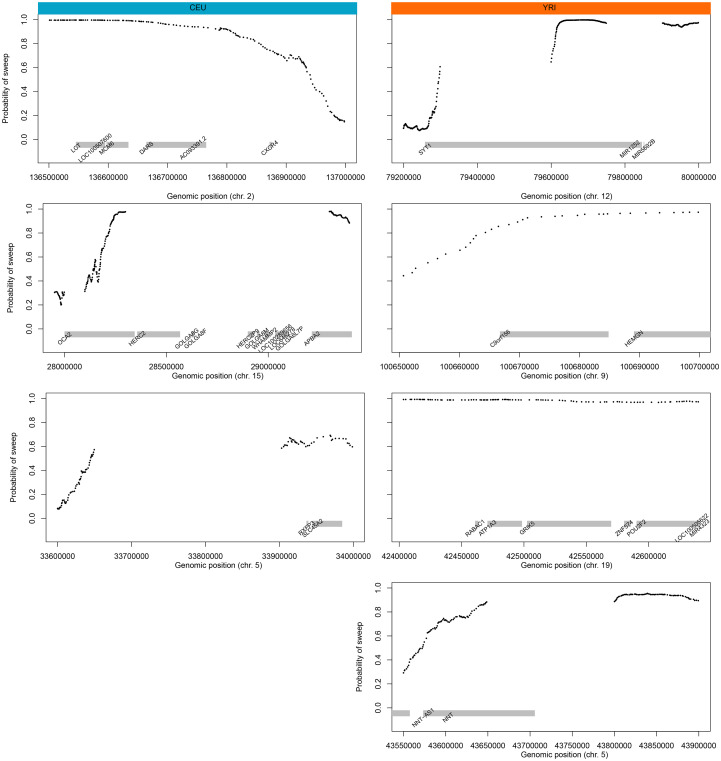
*SURFDAWave* classifier’s application to empirical data for CEU (left column) and YRI (right column) populations. Probability of sweep across the genomic region of labeled chromosome containing the genes of interest. *SURFDAWave* is trained to differentiate between selective sweeps and neutrality with simulations conducted under demographic specifications of the CEU or YRI demographic history. The black dots show the predicted probability of sweep and the gray bars show the positions of the labeled genes. Gaps between black dots are the result of filtering low quality genomic regions (see *Application of empirical data*), such that no SNPs exist in these regions and can therefore not be classified (see S33 Fig as an example of how we classify a SNP spanned by our feature vector).

Using variant calls in the CEU and YRI populations from the 1000 Genomes dataset [32], *SURFDAwave* recapitulated many of the classical sweep candidates observed by other studies, and moreover classified the vast majority of the CEU and YRI genomes as neutral (S14 and S15 Tables), with a greater percentage of the YRI genome being classified as neutral than the CEU genome. This is the result of a combination of factors, including our classifiers reduced ability to distinguish sweeps from neutrality in populations with complex demographic histories, such as the CEU population (see *Classification of selective sweeps*). To make our method more conservative, we applied a probability threshold for selective sweeps. If the probability of a selective sweep is less than or equal to 0.7, then we consider this region to be neutral. S27 Fig shows that the *SURFDAWave* classifier predicts probability distributions close to actual probability distributions, which validates our use of a probability threshold. In addition, we believe our use of balanced training data with an equal number of simulations for each class contributes to the calibrated classifiers. Among the genes classified as selective sweeps in the CEU population, we found *LCT*, *OCA2*, and *SLC45A2*, which were previously hypothesized as targets of selection [33, 34, 35, 36] (Fig 8). In the YRI population we classify the genes *SYT1*, *HEMGN*, *GRIK5*, and *NNT* as under positive selection, recapitulating the work of refs. [37], [38], and [39] (Fig 8). In addition, we also compute the proportion of shared sweeps between these populations by calculating the proportion of non-overlapping 10 kb segments that were classified as sweeps in YRI, that are also classified as sweeps in CEU, as well as the opposite [protocol as in 9]. We find that 21% of sweeps classified as such in CEU are also classified as sweep in YRI. Similarly, we find 19% percent of sweep classifications in YRI are shared by CEU.

As we have already trained models to jointly predict the selection strength, the time at which the mutation became beneficial, and the frequency of the adaptive mutation before becoming beneficial, we next use all of the human genome regions classified as sweeps to learn about the underlying parameters shaping variation at these candidates. We first examined *OCA2*, a gene that is involved in eye coloration [40, 41, 42], and predicted that the time at which a mutation on this gene became beneficial was 1,802 generations ago, and that the beneficial mutation had a selection strength of *s* = 0.06 and an initial frequency of *f* = 0.02. This prediction is made on the set of statistics classified as sweep with the highest probability in the region containing the gene *OCA2* with 0.978 probability. Using a generation time of 29 years for humans, implies the mutation became beneficial about 52,258 years ago, a time during which modern humans were relatively new to Europe [43]. *SLC45A2*, another gene involved in pigmentation [44], harbors a test window with a sweep probability of 0.694 and the predicted selection strength, initial frequency, and selection time are *s* = 0.04, *f* = 0.02, and 2,000 generations ago, respectively. In the YRI population we predict that a mutation on *HEMGN*, a gene that regulates the development of blood cells [45], first became beneficial 1,960 generations ago and has a selection coefficient of *s* = 0.03 and frequency at which it became beneficial of *f* = 0.016. We predict that the selective sweep occurring on the region around *SYT1*, mutations on which are associated with neurodevelopmental disorders [46], began 2,260 generations ago with a selection coefficient of *s* = 0.04 and an initial frequency of *f* = 0.02.

In the list of 444 genes in YRI classified as sweep with probability greater than or equal to 0.7, we examine the range of predictions for each parameter and the genes predicted to have selection parameters at the fringes of each range. For each gene, we only include the prediction for the feature vector where the predicted probability of classification as sweep is the highest within that gene. We find that the gene with the minimum selection coefficient within this list is *HCG23*, with an inferred coefficient of *s* = 0.018. We inferred that a sweep initiated on this gene 1,259 generations ago when the initial frequency of the beneficial mutation was *f* = 0.017. The highest probability of this gene being classified as a sweep is 0.986. We also predict that the gene with the highest initial frequency also had the most recent sweep initiation time. This gene, *STPG2* (Sperm-tail PG rich repeat containing protein 2), is highly expressed in the testis [47], and we predict that this had a mutation reach a frequency of *f* = 0.039 about 666 generations ago, at which point it was predicted to become beneficial.

In a similar examination of the CEU population, we find 2,265 genes are classified as sweep with a probability greater than 0.7. The oldest selection time we predict (2,922 generations or 84,738 years, with a generation time of 29 years) occurred on *VPS35*, a gene on which mutations are associated with Parkinson’s Disease [48]. We infer that strong selection (*s* = 0.05) began on this gene when a selected mutation reached a frequency of *f* = 0.016. *HLA-DRB1* plays an important role in the immune system and has been previously predicted to be under balancing selection [49]. We find that this gene has the highest inferred selection coefficient of *s* = 0.14 and lowest inferred initial frequency of *f* = 0.004 out of our set of genes for the CEU. This may be indicative of a mutation around this region becoming immediately beneficial after it occurred, which we predict was about 1,718 generations ago.

We compare the distributions of selection parameters for the sets of likely selected genes discussed above. In S28 Fig, we can see that while some genes are predicted to have more recent times of selection, most are predicted to have a time of selection greater than 2000 generations ago in both populations. Among the more recent sweeps, we also find a greater range of predicted initial frequencies than those genes that were predicted to have an earlier selection start time. Overall, the distributions for predicted parameters in both populations overlap extensively for all selection parameters (Fig 7). We also observe that *SURFDAWave*’s prediction of the initial frequency (*f*) is not dependent on the probability of a sweep (S29 Fig), but as the probability of sweep increases *SURFDAWave* is are more likely to predict stronger selection coefficients (*s*) and slightly more recent selection start times.

Finally, we apply the classification and prediction models to locate adaptive introgression and learn the adaptive introgression parameters. We find that regardless of the types of statistics used, we classify the majority of the genome as neutral, and classify more of the genome as sweep than as adaptive introgression (S16–S19 Tables). Importantly, we find that we are able to recapitulate signals of previously-identified regions of adaptive introgression in the CEU population with *SURFDAWave*, such as *BNC2* [50, 51] and *APOL4* [4] (S30 Fig). *BNC2* is another gene thought to play a role in human skin color determination [52], whereas the gene *APOL4* is significantly up-regulated in people diagnosed with schizophrenia [53]. By applying the *SURFDAWave* prediction models to the summary statistic computed at these genes, we estimate that the beneficial mutation in *APOL4* reached an initial frequency of *f* = 0.05 and had a selection strength of *s* = 0.01, with the donor and recipient populations splitting 19,760 generations, or about 573,000 years, ago (using a generation time of 29 years). We also estimate that the selection strength on the *BNC2* gene is stronger and harder than the signature on *APOL4*, with *s* = 0.04 and *f* = 0.01. Moreover, the predicted donor and recipient split time of 20,180 generations (585,220 years) ago from variation at *BNC2* is similar to the estimate from *APOL4*.

## Discussion

In this article, we demonstrated that *SURFDAWave* is able to locate selective sweeps, and also predict selection parameters responsible for shaping those sweeps. Moreover, we showed that *SURFDAWave* is capable of differentiating between sweeps and neutrality, and is also able to accurately predict the time at which the selected mutation became beneficial, the frequency a mutation reached before becoming beneficial, and the selection coefficient. In addition, using image-based feature vectors increased our ability to differentiate among neutrality, adaptive introgression, and sweeps. We were able to recapitulate earlier findings by predicting genes as adaptive that were previously hypothesized to be under positive selection.

Our results show that capturing the spatial distribution of selective sweeps is informative for identifying and differentiating between different types of adaptive regions and learning about the evolutionary parameters that shape them. Differentiating between the loss of diversity resulting in adaptive introgression compared to selective sweeps requires a method to learn the wave-like pattern formed by each, the most informative portion of which will be the difference between the crest and trough regions. Moreover, our *SURFDAWave* approach is not restricted to application on adaptive introgression and selective sweep scenarios, and can be implemented for probing genomic variation of other evolutionary processes that leave a spatial or temporal signature in genomic data. Such examples include the identification of genomic targets of balancing selection [*e.g*., 54, 55, 56, 57, 58, 59], complex forms of adaptation such as staggered selective sweeps [60] that have yet to be interrogated for in genomic data, and non-adaptive processes such as recombination rate estimation [*e.g*., 8, 7, 61].

There are a number of potential applications of our methodological framework. For one, it is possible to naturally extend *SURFDAWave* to incorporate genomic data from ancient samples, and several recent studies have employed ancient DNA to directly examine temporal allele frequency fluctuations to identify positively-selected loci [*e.g*., 62, 63, 14, 64, 65, 66]. *SURFDAWave*’s framework would allow examination of changes in the spatial distribution of genetic diversity over time by incorporating information from ancient genomes of a single population at various time points throughout history, and summarizing patterns of variation using two-dimensional wavelet bases. However, a specific limitation of the implementation of *SURFDAWave* as we describe it here is that, for each dimension, its application is restricted to using feature vectors of length *p* in which log_2_(*p*) is a non-negative integer. We acknowledge that this constraint may make it difficult for *SURFDAWave* to be widely applied, especially when incorporating information from ancient DNA. Though we choose to use wavelets in our implementation, other basis functions that do not have such limitations on numbers of features, such as B-spline and polynomial basis functions [11], can be used instead. However, unlike wavelets, these bases do not form orthonormal basis functions, and using them results in more complicated functional regression models.

Along with *SURFDAWave*’s flexibility in terms of classification problems, we also demonstrated that this framework can be adapted to predict different selection parameters. Our results suggest that *SURFDAWave* can predict split time of the donor and recipient populations (S26 Fig). It is possible, however, that introgression patterns in species in which donor and recipient populations have greater divergence times would leave a more prominent footprint (*i.e*., a larger difference between the crest and trough positions), and allow better predictions of their divergence time to be made (Fig 4).

We observe several interesting patterns in our results that may point to potential limitations of *SURFDAWave*. In Fig 7 we see that our prediction of initial frequencies for both the CEU and YRI fall within the range 0.01 to 0.03. Because we are limiting our analysis to sweeps classified with a probability greater than 0.7, we believe this range of initial frequencies is likely most detectable as a sweep. In addition, though there is evidence that hard sweeps are rare in human populations [67], it is difficult with *SURFDAWave* to predict an initial frequency resulting in a hard sweep from a *de novo* mutation because such sweeps are the result of an initial frequency of 1/(2*N*). This frequency is at the boundary of the distribution of our training data. Moreover, the definition of hard sweep may also differ among situations and between research groups. For example, a single beneficial mutation increasing in frequency does so along with a genetic background, and in populations with low diversity with similar genetic backgrounds it may be possible to observe hard sweeps of a single genomic background at high frequency even if the beneficial allele was selected when it was at a frequency greater than 1/(2*N*). Furthermore, the difference in genomic footprints between sweeps resulting from initial frequency 1/(2*N*) and those from frequency *x*/(2*N*) for small *x* ∈ {2, 3, …}, may be difficult to observe due to the hardening of soft sweeps phenomenon [68, 31]. For these reasons, we believe that sweeps lie on a continuum of softness, and predicting the initial frequency of a sweep provides value beyond discrete classification of a sweep as hard or soft. Other evolutionary processes such as gene conversion, may also influence linkage disequilibrium patterns and potentially affect our parameter inference [69]. However, gene conversion tracts are usually short, and because *SURFDAWav*e examines a long physical genomic region we believe these inferences should be minimally affected [69].

Another potential limitation of *SURFDAWave* is that it does not make use of donor genome information in the case of classification for adaptive introgression. Though genome sequences exist for Neanderthal and Denisova, there are many cases in which such data does not, and may never exist [70, 71]. One such example is introgression in African populations. Environmental conditions in the continent could mean a reference genome for the donor population may never be possible [72]. For this reason we designed *SURFDAWave* to be flexible and allow applications for which donor reference data does not exist. However, recent methods have been developed to identify introgressed regions without the requirement of a reference genome [73, 74], making it possible to narrow the locations of adaptive introgression to introgressed regions identified by other methods. Because genome sequence information for African donor archaic populations does not exist [75], we cannot infer parameters such as divergence times with modern humans. However, this information does exist in the case of Neanderthal introgression with non-African populations, and incorporating it will reduce uncertainty in simulation parameters used to train models leading to improved classification and predictions. Estimation of parameters such as divergence time between donor and target populations, time of admixture, admixture fraction from the donor, and population size of the donor can be improved if donor reference genome sequence data exists. In addition, recent methods estimating some of these parameters without reference data for donor populations introgressing into Africans can also be used to make simulations for these cases more realistic and narrow down the parameter range for which simulated replicates are drawn [76]. Estimating these parameters using *SURFDAWave* trained across a range may also provide information about potential donor populations given archaeological and anthropological knowledge about populations given their geographical ranges.

In addition, *SURFDAWave* is currently designed to detect and analyze putative selected regions using information from a single population. However, incorporating multiple populations would likely provide greater power to not only detect selection, but predict selection parameters as well [77, 78]. Including other populations allows the use of statistics such as XP-EHH that can identify selected loci by looking at population differentiation [79]. In addition, likelihood methods modeling differentiation between populations find that including an additional population allows better localization of the beneficial mutation as well as yields higher detection power [80]. Though we have demonstrated the utility of employing wavelets in a statistical learning framework to detect selected loci and predict selection parameters, *SURFDAWave* along with other machine learning approaches [*e.g*., 19, 81, 82, 6, 9] could be made more powerful by employing summary statistics that examine diversity within and differentiation among multiple populations jointly [*e.g*., 77]. Specifically, the application to distinguishing scenarios of adaptive introgression and non-introgression sweeps may benefit substantially by using information from other populations such as with the S* statistic [83, 84, 85, 51] and other multi-population measures [86, 87, 88].

Both sweeps and adaptive introgression result in a decrease of haplotypic diversity (and increase in haplotype similarity) surrounding the beneficial mutation. In soft sweeps this decrease is less dramatic than in hard sweeps, making the spatial distribution of diversity in soft sweeps potentially appear more like that of adaptive introgression. For this reason, it is imperative to utilize summary statistics that capture the sometimes subtle differences between these two evolutionary mechanisms. Specifically adaptive introgression leads to a decrease in mean pairwise sequence difference below the neutral baseline nearby the selected locus, followed by increase above the neutral baseline (or rest position) at moderate distances (“adaptive ridges”) forming the crest of the wave, and then a relaxation to the neutral baseline levels far from the site under selection [4, and as demonstrated in Fig 4]. In contrast, hard sweeps do not display this increase in nucleotide diversity at moderate distances from the selected locus, and soft sweeps do not substantially alter the site frequency spectrum [89] and therefore the mean pairwise sequence difference, which is a summary of this spectrum. Moreover, ref. [4] shows that their method for detecting adaptive introgression from distortions in the site frequency spectrum has the ability to uncover soft adaptive introgression sweeps from multiple introgressed haplotypes, demonstrating that there is even a difference in the spatial signature of nucleotide diversity for soft sweeps and soft introgression sweeps. Indeed, the authors note that both hard and soft introgression sweeps leave more similar genomic footprints to each other than do non-introgression hard and soft sweeps, with both modes of introgression sweeps displaying the crests and troughs of nucleotide diversity characteristic of adaptive introgression. As we notice a substantial increase in differentiation between sweeps and adaptive introgression when including the mean, variance, skewness, and kurtosis of the squared correlation coefficient (*r*^2^) of pairwise windows (Fig 4), we believe these statics might also be capturing some of these signatures, such as the “adaptive ridges” observed in Fig 4. Other statistics, such as ones that assess sequence differences between the top two most-frequent haplotype may aid in distinguishing between soft sweeps and adaptive introgression, for which there may be similar haplotype distributions, but with likely greater haplotype divergence between the most frequent haplotypes under adaptive introgression [86, 90].

We show how incorporating different types of features, specifically two-dimensional statistics, such as the *r*^2^ measured in pairwise windows mentioned above, improves the classification ability of *SURFDAWave* (Fig 4). Several recent innovative approaches have explored the use of image-based or two-dimensional features to predict population-genetic processes. For example, ref. [7] uses the derived or ancestral states from population simulation data directly rather than extracting information from these simulations through the use of summary statistics, and convert this information to images. This raw information can also be converted into wavelet data prior using it as a feature in classification or prediction models. Along with the flexibility that *SURFDAWave* provides in terms of feature input (*e.g*., one- or two-dimensional statistics), other potential enhancements may increase its prediction and classification accuracy. In our application we assume a linear model. However, it is possible that a linear model is not an accurate representation, and instead employing a more flexible model would enhance our predictions if the actual relationship is non-linear. Therefore, using non-linear model such as a neural network with at least one hidden layer [91, 92] in place of simple linear and logistic regression models may be able to improve the performance of *SURFDAWave*. An implementation of *SURFDAWave* along with results for genome wide scans for sweeps discussed in this article can be downloaded from http://degiorgiogroup.fau.edu/surfdawave.html.

## Materials and methods

### Wavelet estimation of summary statistic spatial distribution

Consider a sample of *n* training examples, in which *m* summary statistics are computed at *p* positions along a genomic region. Let **x**_*i*,*s*_ = [*x*_*i*,*s*,1_, *x*_*i*,*s*,2_, …, *x*_*i*,*s*,*p*_]^*T*^ denote the vector of values for summary statistic *s*, *s* = 1, 2, …, *m*, for training example *i*, *i* = 1, 2, …, *n* calculated at each of the *p* positions in a genomic region, where *x*_*i*,*s*,*j*_ is the value of the summary statistic at position *t*_*j*_, *j* = 1, 2, …, *p*. For convenience, define the vector $x_{i}={\left[x_{i,1}^{T},x_{i,2}^{T},\ldots ,x_{i,m}^{T}\right]}^{T}$ containing the values of each of the *m* summary statistics calculated at the *p* positions.

Each vector of summary of summary statistics **x**_*i*,*s*_ is the result of some unknown function *f*_*i*,*s*_(*t*) defined on genomic position *t*. The relationship between the function and the summary statistic data points can be represented as

$$\begin{array}{c} x_{i,s,j}=f_{i,s}\left(t_{j}\right)+\epsilon_{i,s,j}, \end{array}$$

where *f*_*i*,*s*_(*t*_*j*_) is the function *f*_*i*,*s*_(*t*) evaluated at position *t*_*j*_ of summary statistics *s* in observation *i*, and where *ϵ*_*i*,*s*,*j*_ is an error term associated with observation *i* that is normally distributed with mean zero and standard deviation one. As in ref. [11], we can approximate this function *f*_*i*,*s*_(*t*) as a linear combination of a set of *B* orthonormal basis functions {*φ*_1_(*t*), *φ*_2_(*t*), …, *φ*_*B*_(*t*)} as

$$\begin{array}{c} f_{i,s}\left(t\right)\approx \sum_{b=1}^{B}c_{i,s,b}\varphi_{b}\left(t\right), \end{array}$$

where *c*_*i*,*s*,*b*_, *b* = 1, 2, …, *B*, denotes the coefficient of the *b*th basis function *φ*_*b*_(*t*) associated with summary statistic *s* of observation *i*. Note by definition of the *B* basis functions being orthonormal, we have

$$\begin{array}{c} \int {\left[\varphi_{b}\left(t\right)\right]}^{2}dt=1 \end{array}$$

for *b* = 1, 2, …, *B* and

$$\begin{array}{c} \int \varphi_{a}\left(t\right)\varphi_{b}\left(t\right)dt=0 \end{array}$$

for *a* ≠ *b* [93]. Orthonormal basis functions commonly used in functional data analysis include wavelets [94] and the Fourier functions [11]. The number *B* of basis functions is a parameter, and is chosen through cross validation. Basis functions are independent functions that can be combined to approximate more complex functions.

Here we choose to use wavelets as our basis function in part because of their ability to capture information at different resolutions or “detail levels”. Each of these detail levels are captured through combinations of pairs of wavelets termed “mother” and “father” wavelet functions, the breakdown of which is illustrated with an example in Fig 1. The father wavelet function is often referred to as the scaling function, while the mother wavelet function is often called the wavelet function. Each of these wavelet functions captures a different aspect of the data, the father captures “low-frequency” signals, while the mother captures more detailed or “high-frequency” trends [95]. For the purpose of simplicity we discuss the use of Haar wavelets for illustration, however the process differs for other wavelets. We provide a mathematical treatment for Haar wavelets and reference for the mathematical form of Daubechies’ least-asymmetric wavelets below. For Haar wavelets, a feature vector with *p* = 2^*J*^ features undergoes discrete wavelet transformation through subsequent pairwise addition (for father wavelet coefficients) and subtraction (for mother wavelet coefficients). The process of discrete wavelet transform begins at the most detailed level (level *J* − 1) and proceeds until the coarsest detail level (level zero). For each round of transformation, the number of coefficients is half the number in the previous level. This process continues until the number of coefficients is one. These coefficients can then be used as inputs for the wavelet basis functions. The Haar wavelet functions are

$$\begin{array}{c} \psi \left(t\right)=\{\begin{array}{cc} 1 & 0\leq t<1/2\\ -1 & 1/2\leq t<1\\ 0 & \text{otherwise}, \end{array} \end{array}$$

for the mother wavelet function and

$$\begin{array}{c} \phi \left(t\right)=\{\begin{array}{cc} 1 & 0\leq t<1\\ 0 & \text{otherwise}, \end{array} \end{array}$$

for the father wavelet function. For other wavelet types, these functions will differ. For each detail level (or scale) *j* and location *k*, *k* ∈ {0, 1, …, 2^*j*^ − 1} where location is the wavelet number per level, we can respectively define the mother and father wavelet basis functions as

$$\begin{array}{c} \psi_{j,k}\left(t\right)=2^{-j/2}\psi \left(2^{-j}t-k\right), \end{array}$$

and

$$\begin{array}{c} \phi_{j,k}\left(t\right)=2^{-j/2}\phi \left(2^{-j}t-k\right). \end{array}$$

The functions for Daubechies’ least-asymmetric wavelets are more complex and can be examined in ref. [96]. Here we approximate the function *f*_*i*,*s*_(*t*) using wavelets at a detail level of *j*_0_ [97] as

$$\begin{array}{c} f_{i,s,j_{0}}\left(t\right)=\sum_{k=0}^{2^{j_{0}}-1}c_{i,s,j_{0},k}\phi_{j_{0},k}\left(t\right)+\sum_{j=j_{0}}^{J-1}\sum_{k=0}^{2^{j}-1}d_{i,s,j,k}\psi_{j,k}\left(t\right), \end{array}$$

where *J* = log_2_(*p*) is the number of detail levels, *ϕ*_*j*,*k*_(*t*) and *ψ*_*j*,*k*_(*t*) are the the father and mother wavelet basis functions at scale *j* and location *k*, respectively, and *c*_*i*,*s*,*j*,*k*_ and *d*_*i*,*s*,*j*,*k*_ are the coefficients for the father and mother wavelets at scale *j* and location *k* for summary statistic *s* in observation *i*. Note that the father and mother wavelet bases form an orthonormal basis [93]. Moreover, regardless of the chosen detail level *j*_0_, the number of distinct wavelet coefficients and bases used to compute $f_{i,s,j_{0}}$ is 2^*J*^, as

$$\begin{array}{cc} \sum_{k=0}^{2^{j_{0}}-1}1+\sum_{j=j_{0}}^{J-1}\sum_{k=0}^{2^{j}-1}1 & =2^{j_{0}}+\sum_{j=j_{0}}^{J-1}2^{j}\\ & =2^{j_{0}}+\sum_{j=0}^{J-1}2^{j}-\sum_{j=0}^{j_{0}-1}2^{j}\\ & =2^{j_{0}}+\frac{1-2^{J}}{1-2}-\frac{1-2^{j_{0}}}{1-2}\\ & =2^{J}, \end{array}$$

where we used the identity for geometric series [98]

$$\begin{array}{c} \sum_{j=0}^{n-1}ar^{j}=a\frac{1-r^{n}}{1-r} \end{array}$$

for real constants *a* and *r*, *r* ≠ 1.

### Penalized functional multinomial regression to classify genomic regions

After approximating functions ${\hat{f}}_{i,s,j_{0}}\left(t\right)$ of each summary statistic *s* in observation *i* at detail level *j*_0_, we then use these functions (*i.e*., their associated coefficients) as the independent variables to model multinomial regression. Denote the vector of length *p* = 2^*J*^ containing estimated father and mother basis coefficients for summary statistics *s* in observation *i* at detail level *j*_0_ as

$$\begin{array}{c} \xi_{i,s,j_{0}}={\left[{\hat{c}}_{i,s,j_{0},1},\ldots ,{\hat{c}}_{i,s,j_{0},2^{j_{0}}-1},{\hat{d}}_{i,s,j_{0},0},\ldots ,{\hat{d}}_{i,s,J-1,2^{J-1}-1}\right]}^{T} \end{array}$$

Furthermore, define the concatenated vector of length *m* × *p* of such coefficients across all *m* summary statistics for observation *i* by

$$\begin{array}{c} \xi_{i,j_{0}}={\left[\xi_{i,1,j_{0}}^{T},\xi_{i,2,j_{0}}^{T},\ldots ,\xi_{i,m,j_{0}}^{T}\right]}^{T}. \end{array}$$

As in ref. [99], we model

$$\begin{array}{c} P\left[y_{i}=k\;|\;\xi_{i,j_{0}}\right]=\frac{\eta_{k}\left(\xi_{i,j_{0}}\right)}{\sum_{\ell =1}^{K}\eta_{\ell }\left(\xi_{i,j_{0}}\right)}, \end{array}$$

where

$$\begin{array}{cc} \eta_{\ell }\left(\xi_{i,j_{0}}\right) & =\alpha_{\ell }+\sum_{s=1}^{m}\int_{t_{1}}^{t_{p}}\beta_{\ell ,s}\left(t\right){\hat{f}}_{i,s,j_{0}}\left(t\right)dt\\ & =\alpha_{\ell }+\sum_{s=1}^{m}[\sum_{k=0}^{2^{j_{0}}-1}{\hat{c}}_{i,s,j_{0},k}\int_{t_{1}}^{t_{p}}\beta_{\ell ,s}\left(t\right)\phi_{j_{0},k}\left(t\right)dt+\sum_{j=j_{0}}^{J-1}\sum_{k=0}^{2^{j}-1}{\hat{d}}_{i,s,j,k}\int_{t_{1}}^{t_{p}}\beta_{\ell ,s}\left(t\right)\psi_{j,k}\left(t\right)dt] \end{array}$$

for *ℓ* = 1, 2, …, *K*. This is similar to other multinomial regression models, with the the caveat that we replaced the summation with an integration across the interval [*t*_1_, *t*_*p*_] for position *t*. Here *i* is the index for the observation number, *y*_*i*_ is the categorical response variable with values *y*_*i*_ = *ℓ* for class *ℓ*, for *ℓ* = 1, 2, …, *K*, *α*_*ℓ*_ is the intercept parameter for class *ℓ*, and *β*_*ℓ*,*s*_(*t*) is the function for summary statistic *s* of class *ℓ*.

To learn the functions *β*_*ℓ*,*s*_(*t*), we can note that we may also approximate them with the same set of basis functions as we did for approximating *f*_*i*,*s*_(*t*). That is, we can approximate the function *β*_*ℓ*,*s*_(*t*) using wavelets at a detail level of *j*_0_ as

$$\begin{array}{c} \beta_{\ell ,s,j_{0}}\left(t\right)=\sum_{k=0}^{2^{j_{0}}-1}c_{\ell ,s,j_{0},k}^{⋆}\phi_{j_{0},k}\left(t\right)+\sum_{j=j_{0}}^{J-1}\sum_{k=0}^{2^{j}-1}d_{\ell ,s,j,k}^{⋆}\psi_{j,k}\left(t\right), \end{array}$$

where $c_{\ell ,s,j,k}^{⋆}$ and $d_{\ell ,s,j,k}^{⋆}$ are the coefficients for the father and mother wavelets at scale *j* and location *k* for summary statistic *s* in class *ℓ*. Denote the vector of length *p* = 2^*J*^ containing father and mother basis coefficients for summary statistic *s* for class *ℓ* at detail level *j*_0_ as

$$\begin{array}{c} \zeta_{\ell ,s,j_{0}}={\left[c_{\ell ,s,j_{0},1}^{⋆},\ldots ,c_{\ell ,s,j_{0},2^{j_{0}}-1}^{⋆},d_{\ell ,s,j_{0},0}^{⋆},\ldots ,d_{\ell ,s,J-1,2^{J-1}-1}^{⋆}\right]}^{T}, \end{array}$$

and further define the concatenated vector of length *m* × *p* of such coefficients across all *m* summary statistics for class *ℓ* by

$$\begin{array}{c} \zeta_{\ell ,j_{0}}={\left[\zeta_{\ell ,1,j_{0}}^{T},\zeta_{\ell ,2,j_{0}}^{T},\ldots ,\zeta_{\ell ,m,j_{0}}^{T}\right]}^{T}. \end{array}$$

Plugging in this approximation, and using the orthnormality of the set of basis functions, we obtain

$$\begin{array}{cc} \eta_{\ell }\left(\xi_{i,j_{0}}\right) & =\alpha_{\ell }+\sum_{s=1}^{m}[\sum_{k=0}^{2^{j_{0}}-1}{\hat{c}}_{i,s,j_{0},k}c_{\ell ,s,j_{0},k}^{⋆}+\sum_{j=j_{0}}^{J-1}\sum_{k=0}^{2^{j}-1}{\hat{d}}_{i,s,j,k}d_{\ell ,s,j,k}^{⋆}]\\ & =\alpha_{\ell }+\xi_{i,j_{0}}^{T}\zeta_{\ell ,j_{0}} \end{array}$$

which yields

$$\begin{array}{c} P\left[y_{i}=k\;|\;\xi_{i,j_{0}}\right]=\frac{\text{exp}[\alpha_{k}+\xi_{i,j_{0}}^{T}\zeta_{k,j_{0}}]}{\sum_{\ell =1}^{K}\text{exp}\left[\alpha_{\ell }+\xi_{i,j_{0}}^{T}\zeta_{\ell ,j_{0}}\right]}. \end{array}$$

Let ***α*** = [*α*_1_, *α*_2_, …, *α*_*K*_]^*T*^ denote the vector of intercept terms for each of the *K* classes and define the matrix $Z_{j_{0}}$ containing *m* × *p* rows and *K* columns by

$$\begin{array}{c} Z_{j_{0}}=\left[\zeta_{1,j_{0}},\zeta_{2,j_{0}},\ldots ,\zeta_{K,j_{0}}\right]. \end{array}$$

The log likelihood of observing the set of model parameters $\left\{\alpha ,Z_{j_{0}}\right\}$ given the collection of data points ${\left\{y_{i},\xi_{i,j_{0}}\right\}}_{i=1}^{n}$ is

$$\begin{array}{cc} \text{log}L(\alpha ,Z_{j_{0}}\;;\;{\left\{y_{i},\xi_{i,j_{0}}\right\}}_{i=1}^{n}) & =\frac{1}{n}\sum_{i=1}^{n}\sum_{k=1}^{K}\text{log}P\left[y_{i}\;|\;\xi_{i,j_{0}}\right]1_{\left\{y_{i}=k\right\}}\\ & =\frac{1}{n}\sum_{i=1}^{n}[\sum_{k=1}^{K}\left(\alpha_{k}+\xi_{i,j_{0}}^{T}\zeta_{k,j_{0}}\right)1_{\left\{y_{i}=k\right\}}-\text{log}(\sum_{\ell =1}^{K}\text{exp}\left\{\alpha_{\ell }+\xi_{i,j_{0}}^{T}\zeta_{\ell ,j_{0}}\right\})], \end{array}$$

where **1**_{*y*_*i*_=*k*}_ is an indicator random variable that takes the values one if *y*_*i*_ = *k* and zero otherwise.

From this likelihood function, we wish to estimate the intercept terms ***α*** and the coefficients $Z_{j_{0}}$. Define $\hat{\alpha }$ as an estimate of ***α*** and ${\hat{Z}}_{j_{0}}$ an estimate of $Z_{j_{0}}$. Moreover, as our model is over-parameterized, we need to maximize a penalized log likelihood function. Denoting ∥⋅∥_1_ and ∥⋅∥_2_ as the *ℓ*_1_ and *ℓ*_2_ norms, respectively, define

$$\begin{array}{cc} {\text{PEN}}_{\gamma }\left(Z_{j_{0}}\right) & =\sum_{\ell =1}^{K}(\gamma ∥\zeta_{\ell ,j_{0}}{∥}_{1}+\left(1-\gamma \right){∥\zeta_{\ell ,j_{0}}∥}_{2}^{2})\\ & =\sum_{\ell =1}^{K}\sum_{s=1}^{m}[\sum_{k=0}^{2^{j_{0}}-1}(\gamma |c_{\ell ,s,j_{0},k}^{⋆}|+\left(1-\gamma \right){\left(c_{\ell ,s,j_{0},k}^{⋆}\right)}^{2})+\sum_{j=j_{0}}^{J-1}\sum_{k=0}^{2^{j}-1}(\gamma |d_{\ell ,s,j,k}^{⋆}|+\left(1-\gamma \right){\left(d_{\ell ,s,j,k}^{⋆}\right)}^{2})] \end{array}$$

to be the elastic-net penalty [23] controlled by parameter *γ* ∈ [0, 1] on the coefficients for the basis functions of the regression coefficient functions, and let λ denote a tuning parameter associated with this penalty. A value of *γ* = 0 leads to the standard ridge regression penalty, and *γ* = 1 leads to the lasso penalty. We can therefore estimate the coefficient functions as

(α^,Z^j0,λ^,γ^)=argmaxα,Zj0,λ,γ[logL(α,Zj0;{yi,ξi,j0}i=1n)-λPENγ(Zj0)].

To perform this estimation, we first learn the underlying functions *f*_*i*,*s*_(*t*) based on orthonormal wavelet basis functions at detail level *j*_0_, yielding the estimated set of coefficients ${\left\{{\hat{\xi }}_{i,j_{0}}\right\}}_{i=1}^{n}$ and hence estimated functions

$$\begin{array}{c} {\hat{f}}_{i,s,j_{0}}\left(t\right)=\sum_{k=0}^{2^{j_{0}}-1}{\hat{c}}_{i,s,j_{0},k}\phi_{j_{0},k}\left(t\right)+\sum_{j=j_{0}}^{J-1}\sum_{k=0}^{2^{j}-1}{\hat{d}}_{i,s,j,k}\psi_{j,k}\left(t\right). \end{array}$$

These basis function coefficients are then employed as input covariates to the penalized regression model, for which ten-fold cross validation is used to estimate the tuning parameter λ, the tuning parameter *γ* controlling the elastic-net penalty, and associated parameters ***α*** and $Z_{j_{0}}$. This process is repeated for different detail levels *j*_0_ = 0, 1, …, *J* − 1 to estimate the *j*_0_ that minimizes the ten-fold cross validation error, and the best fitting values of regression model parameters $\hat{\alpha }$ and ${\hat{Z}}_{j_{0}}$ are estimated. These estimates lead to a classifier for future input data, as well as learned functions

$$\begin{array}{c} {\hat{\beta }}_{k,s,j_{0}}\left(t\right)=\sum_{k=0}^{2^{j_{0}}-1}{\hat{c}}_{i,s,j_{0},k}^{⋆}\phi_{j_{0},k}\left(t\right)+\sum_{j=j_{0}}^{J-1}\sum_{k=0}^{2^{j}-1}{\hat{d}}_{i,s,j,k}^{⋆}\psi_{j,k}\left(t\right) \end{array}$$

for summary statistic *s*, *s* = 1, 2, …, *m*, in class *k*, *k* = 1, 2, …, *K*. After parameter inference, the most likely class $\hat{k}$ is estimated as

k^=argmaxk∈{1,2,…,K}exp[α^k+ξ^i,j0Tζ^k,j0]∑ℓ=1Kexp[α^ℓ+ξ^i,j0Tζ^ℓ,j0].

In addition, the probability of each class *k* can be determined by removing the *arg max* portion of the equation as

$$\begin{array}{c} \hat{P}\left(k\right)=\frac{\text{exp}[{\hat{\alpha }}_{k}+{\hat{\xi }}_{i,j_{0}}^{T}{\hat{\zeta }}_{k,j_{0}}]}{\sum_{\ell =1}^{K}\text{exp}[{\hat{\alpha }}_{\ell }+{\hat{\xi }}_{i,j_{0}}^{T}{\hat{\zeta }}_{\ell ,j_{0}}]}, \end{array}$$

which will allow us to use this probability to determine the weight of the classification and use probability thresholds to increase confidence in our results.

### Penalized functional linear regression to infer evolutionary parameters

Once identifying the most likely class $\hat{k}$, we then estimate the underlying evolutionary parameters ***σ*** = [*σ*_1_, *σ*_2_, …, *σ*_*q*_]^*T*^ that gave rise to patterns within the genomic region provided that it was estimated to be non-neutral, where *σ*_1_, *σ*_2_, …, *σ*_*q*_ represent the *q* evolutionary parameters we are estimating for class $\hat{k}$.

Consider again the approximated functions ${\hat{f}}_{i,s,j_{0}}\left(t\right)$ of each summary statistic *s* in observation *i* at detail level *j*_0_. We will use these functions (and as in the preceding section, their associated coefficients) as the independent variables to model multivariate linear regression as

$$\begin{array}{cc} \sigma_{i,\ell } & =\alpha_{\ell }+\sum_{s=1}^{m}\int_{t_{1}}^{t_{p}}\beta_{\ell ,s}\left(t\right){\hat{f}}_{i,s,j_{0}}\left(t\right)dt+\epsilon_{i,\ell }\\ & =\alpha_{\ell }+\sum_{s=1}^{m}[\sum_{k=0}^{2^{j_{0}}-1}{\hat{c}}_{i,s,j_{0},k}\int_{t_{1}}^{t_{p}}\beta_{\ell ,s}\left(t\right)\phi_{j_{0},k}\left(t\right)dt+\sum_{j=j_{0}}^{J-1}\sum_{k=0}^{2^{j}-1}{\hat{d}}_{i,s,j,k}\int_{t_{1}}^{t_{p}}\beta_{\ell ,s}\left(t\right)\psi_{j,k}\left(t\right)dt]+\epsilon_{i,\ell } \end{array}$$

for *ℓ* = 1, 2, …, *q*. Here *i* is the index for the observation number, *σ*_*i*,*ℓ*_ is the response value for evolutionary parameter *σ*_*ℓ*_ of observation *i*, *α*_*ℓ*_ is the intercept for evolutionary parameter *σ*_*ℓ*_, *β*_*ℓ*,*s*_(*t*) is the function for summary statistic *s* of evolutionary parameter *σ*_*ℓ*_, and *ϵ*_*i*,*ℓ*_ is the error associated with observation *i* of evolutionary parameter *σ*_*ℓ*_. Moreover, define the vector of length *q* containing the evolutionary parameters that generated observation *i* by

$$\begin{array}{c} \sigma_{i}={\left[\sigma_{i,1},\sigma_{i,2},\ldots ,\sigma_{i,q}\right]}^{T}. \end{array}$$

As in the preceding section, to learn the functions *β*_*ℓ*,*s*_(*t*) we can approximate them using wavelets at a detail level of *j*_0_ as

$$\begin{array}{c} \beta_{\ell ,s,j_{0}}\left(t\right)=\sum_{k=0}^{2^{j_{0}}-1}c_{\ell ,s,j_{0},k}^{⋆}\phi_{j_{0},k}\left(t\right)+\sum_{j=j_{0}}^{J-1}\sum_{k=0}^{2^{j}-1}d_{\ell ,s,j,k}^{⋆}\psi_{j,k}\left(t\right), \end{array}$$

where $c_{\ell ,s,j,k}^{⋆}$ and $d_{\ell ,s,j,k}^{⋆}$ are the coefficients for the father and mother wavelets at scale *j* and location *k* for summary statistic *s* of evolutionary parameter *σ*_*ℓ*_. Denote the vector of length *p* = 2^*J*^ containing father and mother basis coefficients for summary statistics *s* for evolutionary parameter *σ*_*ℓ*_ at detail level *j*_0_ as

$$\begin{array}{c} \zeta_{\ell ,s,j_{0}}={\left[c_{\ell ,s,j_{0},1}^{⋆},\ldots ,c_{\ell ,s,j_{0},2^{j_{0}}-1}^{⋆},d_{\ell ,s,j_{0},0}^{⋆},\ldots ,d_{\ell ,s,J-1,d^{J-1}-1}^{⋆}\right]}^{T}, \end{array}$$

and further define the concatenated vector of length *m* × *p* of such coefficients across all *m* summary statistics for evolutionary parameter *σ*_*ℓ*_ by

$$\begin{array}{c} \zeta_{\ell ,j_{0}}={\left[\zeta_{\ell ,1,j_{0}}^{T},\zeta_{\ell ,2,j_{0}}^{T},\ldots ,\zeta_{\ell ,m,j_{0}}^{T}\right]}^{T}. \end{array}$$

Plugging in this approximation, and using the orthonormality of the set of basis functions, we obtain

$$\begin{array}{cc} \sigma_{i,\ell } & =\alpha_{\ell }+\sum_{s=1}^{m}[\sum_{k=0}^{2^{j_{0}}-1}{\hat{c}}_{i,s,j_{0},k}c_{\ell ,s,j_{0},k}^{⋆}+\sum_{j=j_{0}}^{J-1}\sum_{k=0}^{2^{j}-1}{\hat{d}}_{i,s,j,k}d_{\ell ,s,j,k}^{⋆}]\\ & =\alpha_{\ell }+\xi_{i,j_{0}}^{T}\zeta_{\ell ,j_{0}}+\epsilon_{i,\ell }. \end{array}$$

Let ***α*** = [*α*_1_, *α*_2_, …, *α*_*q*_]^*T*^ denote the vector of intercept terms for each of the *q* evolutionary parameters and define the matrix $Z_{j_{0}}$ containing *m* × *p* rows and *q* columns by

$$\begin{array}{c} Z_{j_{0}}=\left[\zeta_{1,j_{0}},\zeta_{2,j_{0}},\ldots ,\zeta_{q,j_{0}}\right]. \end{array}$$

The loss function of the collection of data points ${\left\{\sigma_{i},\xi_{i,j_{0}}\right\}}_{i=1}^{n}$ given the set of model parameters $\left\{\alpha ,Z_{j_{0}}\right\}$ is

$$\begin{array}{c} L_{\alpha ,Z_{j_{0}}}\left({\left\{\sigma_{i},\xi_{i,j_{0}}\right\}}_{i=1}^{n}\right)=\sum_{\ell =1}^{q}\sum_{i=1}^{n}{\left(\sigma_{i,\ell }-\alpha_{\ell }-\xi_{i,j_{0}}^{T}\zeta_{\ell ,j_{0}}\right)}^{2}. \end{array}$$

From this loss function, we wish to estimate the intercept terms ***α*** and the coefficients $Z_{j_{0}}$. Define $\hat{\alpha }$ as an estimate of *α* and ${\hat{Z}}_{j_{0}}$ as an estimate of $Z_{j_{0}}$. Similarly to the previous section, define

$$\begin{array}{cc} {\text{PEN}}_{\gamma }\left(Z_{j_{0}}\right) & =\sum_{\ell =1}^{q}(\gamma ∥\zeta_{\ell ,j_{0}}{∥}_{1}+\left(1-\gamma \right){∥\zeta_{\ell ,j_{0}}∥}_{2}^{2})\\ & =\sum_{\ell =1}^{q}\sum_{s=1}^{m}[\sum_{k=0}^{2^{j_{0}}-1}(\gamma |c_{\ell ,s,j_{0},k}^{⋆}|+\left(1-\gamma \right){\left(c_{\ell ,s,j_{0},k}^{⋆}\right)}^{2})+\sum_{j=j_{0}}^{J-1}\sum_{k=0}^{2^{j}-1}(\gamma |d_{\ell ,s,j,k}^{⋆}|+\left(1-\gamma \right){\left(d_{\ell ,s,j,k}^{⋆}\right)}^{2})] \end{array}$$

to be the elastic-net penalty [23] controlled by parameter *γ* ∈ [0, 1] on the coefficients for the basis functions of the regression coefficient functions, and let λ denote a tuning parameter associated with this penalty. We can therefore estimate the coefficient functions as

(α^,Z^j0,λ^,γ^)=argminα,Zj0,λ,γ[Lα,Zj0({σi,ξi,j0}i=1n)+λPENγ(Zj0)].

As in preceding section, we perform this estimation, we first learn the underlying functions *f*_*i*,*s*_(*t*) based on orthonormal wavelet basis functions at detail level *j*_0_, yielding the estimated set of coefficients ${\left\{{\hat{\xi }}_{i,j_{0}}\right\}}_{i=1}^{n}$ and hence estimated functions ${\hat{f}}_{i,s,j_{0}}\left(t\right)$. These basis function coefficients are then input as covariates to the penalized regression model, for which ten-fold cross validation is used to estimate the tuning parameter λ, the tuning parameter *γ* controlling the elastic-net penalty, and associated parameters ***α*** and $Z_{j_{0}}$. This process is repeated for different detail levels *j*_0_ = 0, 1, …, *J* − 1 to estimate the *j*_0_ that minimizes the ten-fold cross validation error, and the best fitting values of regression model parameters $\hat{\alpha }$ and ${\hat{Z}}_{j_{0}}$ are estimated. These estimates lead to an estimator for the *q* underlying evolutionary parameters for future input data, as well as learned functions ${\hat{\beta }}_{\ell ,s,j_{0}}\left(t\right)$ for summary statistic *s*, *s* = 1, 2, …, *m* of evolutionary parameter *σ*_*ℓ*_, *ℓ* = 1, 2, …, *q*. After parameter inference, evolutionary parameter *σ*_*ℓ*_ is estimated as

$$\begin{array}{c} {\hat{\sigma }}_{\ell }={\hat{\alpha }}_{\ell }+{\hat{\xi }}_{i,j_{0}}^{T}{\hat{\zeta }}_{\ell ,j_{0}}. \end{array}$$

### Training the models

For the ten-fold cross validation procedure, we split our training data into ten balanced subsets and supply values of the elastic net parameter (*γ*) we are interested in exploring, with those values being *γ* ∈ {0.0, 0.1, 0.2, …, 1.0}. For each pair of *γ* parameter and detail (scale) level *j*_0_ (*j*_0_ ∈ {0, 1, …, *J* − 1}) combinations, we train a model with 90% of the data and validate with the remaining 10%, iterating through each fold, while keeping count of the percentage of correct classifications for each combination. Conditional on *γ*, the glmnet [100] software that we employ to train elastic net models performs an automatic search across the space of regularization tuning parameter (λ) to identify the optimal value. When all level (*j*_0_) and *γ* combinations have been tested (each associated with an optimal regularization parameter λ) we finally choose the model with the highest percentage of correct classifications, and train a final model using the entire training dataset for these parameters.

### Calculating summary statistics

Informative summary statistics are likely the most important aspect of developing prediction models. In this manuscript we discuss the use of several sets of summary statistics. For our initial comparison, we utilize a similar set of summary statistics as discussed in ref. [9] including the mean pairwise sequence difference ($\hat{\pi }$), *H*_1_, *H*_12_, and *H*_2_/*H*_1_. As shown in ref. [9] *r*^2^ was not informative for classification rates, and for this reason, we omit *r*^2^ as applied in [9] from our model. Moreover, ref. [9] found that haplotype-based statistics were often more informative than site frequency-based statistics, and for this reason we include the frequencies of the first, second, third, fourth, and fifth most common haplotypes. We also removed all sites with minor allele count less than three because this dramatically reduced the differences between simulated and empirical site frequency spectra (S31 Fig). To keep our performance evaluation consistent with the empirical assessment, we also removed these sites for tests with simulations of a constant-size demographic history, although models trained with these simulations were not applied to empirical data.

We calculated each of these *m* = 9 summary statistics in *p* genomic windows across the region of interest, where each window consists of 10 SNPs and overlaps with its neighbors for five SNPs, as shown in S33 Fig. Using SNP-based windows, rather than windows based on physical length has made our method more conservative in classification problems [101, 9]. Because wavelet transformation requires that the number of observations *p* be a power of two, we investigated *p* = 128, leading to 645 SNPs overall used for classification of a genomic region. The classified SNP is the SNP that falls in center of the overlap of windows *p*/2 and *p*/2 + 1, and is taken as the putative location of the site under selection. A schematic illustrating how summary statistics are calculated in *SURFDAWave* is given in S33 Fig.

We also explore the use of other statistics that may better differentiate more complex types of selection, such as the set of mean values of *r*^2^ between each window pair. Each one-dimensional statistic (*i.e*., $\hat{\pi }$, *H*_1_, *H*_12_, *H*_2_/*H*_1_, and the frequencies of first to fifth most common haplotypes) is computed using *p* = 128 overlapping windows, of which 64 are non-overlapping. Because the *r*^2^ statistic calculated between each pair of windows is more time consuming to compute than the one-dimensional statistics, we only compute the *r*^2^ statistic between the *p* = 64 non-overlapping 10-SNP windows, for a total of 64 × (64 + 1)/2 = 2, 080 pairwise *r*^2^ computations across the set of 64 non-overlapping windows. In addition to the mean of *r*^2^, we also use the set of values for the variance, skewness, and kurtosis of *r*^2^ computed at each window pair, with an additional 2,080 pairwise computations for each of these statistics.

### Simulations to test method performance

We designed *SURFDAWave* to learn about adaptation in human populations, so for this reason we focus our simulations on human based parameters. All simulation results use the software SLiM [21]. We use demographic estimates from ref. [20] to model the bottlenecks and expansions experienced by human populations. In addition we also simulate a constant-size demographic history with an effective population size of *N* = 10^4^ [102] diploid individuals. For all demographic histories we use a mutation rate of 1.25 × 10^−8^ per site per generation [22] and recombination rate drawn from an exponential distribution with mean 3 × 10^−9^ per site per generation [20] and truncated at three times the mean [27] to simulate genomic regions of length two Mb. In addition, we include for comparison two varying recombination rate scenarios, one in which we use a recombination rate drawn from an exponential distribution with mean 10^−8^ per site per generation, and truncated at three times the mean [27], and another in which we draw recombination rate from a human recombination map [103]. Specifically, we randomly draw two Mb regions from a CEU-based recombination map and use the rate and location in those regions to simulate. For selection simulations we let a mutation occur in a generation drawn uniformly at random between 1,020 and 3,000 generations ago, and set this mutation as beneficial with selection strength *s* ∈ [0.005, 0.5] per generation (drawn uniformly at random on a log scale) once it reached frequency *f* ∈ [1/(2*N*), 0.1] (drawn uniformly at random on a log scale). This results in our selection simulations containing both hard and soft sweeps. Some combinations of selection parameters are difficult to achieve and for this reason may be under-represented in our simulations (compared to our input parameters) (S34 Fig).

To test the performance of *SURFDAWave* on more complex selection scenarios we simulate adaptive introgression. To do this, we simulate a single population that splits into two populations (a recipient and a donor) at a time randomly selected between 13,000 and 32,000 generations ago. This range captures the predicted split times among human, Neanderthal, and Denisovan populations [104]. After allowing the two populations to evolve in isolation, we then simulate a neutral mutation in the center of the two Mb chromosome to occur between 1,020 and 3,000 generations ago in the simulated donor population. Following this, the donor population admixes into the recipient population in which the donor replace between 1 to 10% of the recipient population. After admixture, we treat the simulation as a regular sweep setting and follow the protocol described for sweep simulations by allowing the neutral mutation to attain a certain frequency *f* ∈ [1/(2*N*), 0.1] before converting it to beneficial.

We also simulated background selection following the protocol described in ref. [27], in which purifying selection is simulated by setting a negative selection coefficient if mutations fall within simulated coding regions. The distribution of coding regions is drawn from both the phastCons [105] and GENCODE [106] databases. Uniformly choosing a random starting point as a SNP in the human genome, we simulate 10^3^, two Mb chromosomes with 75% of mutations falling within coding regions to have a selection coefficient drawn from a gamma distribution with mean −0.0294 with the remaining 25% as neutral, which models the distribution of fitness effects consistent with the human genome [107]. As in ref. [9], we simulated missing data by removing thirty percent of the simulated SNPs in blocks, with each of ten non-overlapping blocks containing 3% of the total data. This process simulates the effects of filters that remove regions of low mappability or alignability [25]. To test the accuracy of our prediction models we also simulate 1000 sweeps with background selection.

In order to test the performance of *SURFDAWave* on species other than humans we simulate both sweeps and neutrality using *Drosophila* demographic parameters as adapted from ref. [29] in both refs. [31] and [30]. Demographic parameters such as population split times and effective population sizes are drawn from posterior distributions of their estimates [Table S1 of ref. 30]. As in ref. [31], we used the coalescent-simulator *ms* [108] to generate neutral variation (burn-in) for its speed, and used the output from these simulations to seed the haplotypic variation within SLiM [21], and employed a recombination rate of 5 × 10^−9^ per site per generation and mutation rate of 10^−9^ per site per generation as in ref. [30]. For selection settings, we simulate a mutation to occur again in a generation drawn from between 1,020 and 3,000 generations ago and once it attains frequency *f* ∈ [1/(2*N*), 0.1] we set its selection coefficient to *s* ∈ [0.005, 0.5] per generation. These selection parameters are the same as the ones used in human simulations as we wanted to analyze the effects of species demographic history.

Because we are unsure of how much training data is required to adequately fit models with our noisy data, we conducted an experiment to see how different numbers of training data points per class affect classification rates (S32 Fig). We include in our training data either 1000, 3000, 5000, or 7000 feature vectors for each class (adaptive introgression, neutrality, or sweep). As we increase the number of training data points per class we see an increase in the number of simulations classified correctly for both sweep and adaptive introgression classes. However, we also observe a slight decrease in the number of neutral simulations classified correctly between 5000 and 7000 simulations per class. Although the overall percent correct is greatest when using 7000 per class as expected, we use 5000 simulations per class both because of the higher neutral classification accuracy and because using 5000 simulations takes less time than using 7000.

### Comparison to *Trendsetter*, diploS/HIC, and evolBoosting

For all classification problems analyzed in this manuscript we provided comparison to other recently-developed methods for classifying selective sweeps [19, 6, 9]. For both evolBoosting and diploS/HIC we apply these methods using their default settings, in terms of window length, window size, and statistics used by the classifier. However, we modify diploS/HIC to be used as a binary (or three class for adaptive introgression settings) classifier without requiring “linked-sweep” classes. This is important, as summary statistics chosen for diploS/HIC may have been optimized for a five-class setting, and the overall performance of diploS/HIC may diverge from what we present here if the default separate “hard sweep”, “soft sweep”, “linked-hard sweep”, and “linked-soft sweep” classes were included. In addition we use the summary statistics $\hat{\pi }$, *H*_1_, *H*_12_, *H*_2_/*H*_1_, and frequency of the first, second, third, fourth, and fifth most common haplotypes calculated at 128 windows as input data for training both in *Trendsetter* and *SURFDAWave*, making this implementation slightly different than that published in ref. [9]. We believe that this slight alteration of *Trendsetter* is much less important than the reduction of diploS/HIC to a binary classifier. In addition to classification results, we also provide a run time comparison.

To compare the runtime, we used the time command in bash on our workstation running the Centos 7 operating system with two 2.10 GHz processors, each with six cores that are able to run two threads (for a total of 24 threads). For comparison purposes we used an identical set of training simulations conducted under constant demographic history with 5000 simulations for each of the two classes (sweep and neutral). We report the “real” or wall clock time required for training each of the classification methods we used in our comparisons (S20 Table), once the feature vectors were already computed. We find that *Trendsetter* is the slowest method, owing to the fact that we used the *d* = 2 linear trend penalty in our analysis. *SURFDAWave* is the second slowest taking approximately 1400 seconds to run. Both diploS/HIC and evolBoosting have much faster training times. Comparatively, however, both of these methods require much longer to calculate feature vectors from data relative to both *SURFDAWave* and *Trendsetter*.

### Application to empirical data

To locate regions of selection in human genomes, we conducted scans using phased haplotype data from the central European (CEU) and sub-Saharan African Yoruban (YRI) populations in the 1000 Genomes Project dataset [32]. Because some genomic regions are difficult to sequence, map, or align, and result in low quality data that is prone to errors, we split the genomes into 100 kb non-overlapping segments, and removed those with mean CRG100 score less than 0.9 [109]. Though this does result in some statistics being calculated in windows spanning large genomic regions, we find that because we are using SNP-based windows *SURFDAWave* is more likely to be conservative and classify these windows as neutral (Fig 6). Moreover, *SURFDAWave* classifies the window centered on the SNP in the middle of windows *p*/2 and *p*/2 + 1 (*e.g*., see S33 Fig), and as a result, no filtered regions will be classified as no SNPs reside in these filtered regions. As described in *Calculating summary statsitics*, we also removed all sites with minor allele frequency less than three. We then split the remaining data for each chromosome into windows of 10 SNPs where each window overlaps its neighbor for five SNPs, and computed summary statistics discussed in section *Calculating summary statistics* for each window. As we are investigating *p* = 128, each set of statistics for 128 windows comprises a feature vector. When scanning the genome, we shift one window at a time, so that the putative site of selection (the middle SNP falling in the overlap of windows *p*/2 and *p*/2 + 1) will shift by five SNPs each iteration. These feature vectors are used as input to both the *SURFDAWave* classifier and predictor. As we value the correct classification of neutral genomic regions, we use 5000 simulated replicates of each class to train classifiers, because we notice a decrease in the number of correctly classified neutral regions when we use more (S32 Fig).

## Supporting information

S1 Table Root mean squared error (RMSE) and mean absolute error (MAE) values when predicting selection coefficient (*s*), initial frequency (*f*), and time of selection (*T*_sel_) for YRI and CEU populations. The values show RMSE and MAE measured between standardized log-scaled predicted and actual parameters in simulated data.
(PDF)

S2 Table Root mean squared error (RMSE) and mean absolute error (MAE) values when predicting selection coefficient (*s*), initial frequency (*f*), and time of selection (*T*_sel_) for YRI and CEU populations. The values show RMSE and MAE measured between log-scaled predicted and actual parameters after unstandardizing.
(PDF)

S3 Table Root mean squared error (RMSE) and mean absolute error (MAE) values when predicting selection coefficient (*s*), initial frequency (*f*), and time of selection (*T*_sel_) for YRI and CEU populations tested on simulations of missing data. The values show RMSE and MAE measured between standardized log-scaled predicted and actual parameters in simulated data.
(PDF)

S4 Table Root mean squared error (RMSE) and mean absolute error (MAE) values when predicting selection coefficient (*s*), initial frequency (*f*), and time of selection (*T*_sel_) for YRI and CEU populations tested on simulations of missing data. The values show RMSE and MAE measured between log-scaled predicted and actual parameters after unstandardizing.
(PDF)

S5 Table Root mean squared error (RMSE) and mean absolute error (MAE) values when predicting selection coefficient (*s*), initial frequency (*f*), and time of selection (*T*_sel_) for YRI and CEU populations when tested with models trained with simulations of the opposite demography. The values show RMSE and MAE measured between standardized log-scaled predicted and actual parameters in simulated data.
(PDF)

S6 Table Root mean squared error (RMSE) and mean absolute error (MAE) values when predicting selection coefficient (*s*), initial frequency (*f*), and time of selection (*T*_sel_) for YRI and CEU populations when tested with models trained with simulations of the opposite demography. The values show RMSE and MAE measured between log-scaled predicted and actual parameters after unstandardizing.
(PDF)

S7 Table Root mean squared error (RMSE) and mean absolute error (MAE) values when predicting selection coefficient (*s*), initial frequency (*f*), and time of selection (*T*_sel_) for CEU and YRI populations when trained with simulations conducted under both YRI and CEU demographic histories and tested with the specified (CEU or YRI) demographic history. The values show RMSE and MAE measured between standardized log-scaled predicted and actual parameters.
(PDF)

S8 Table Root mean squared error (RMSE) and mean absolute error (MAE) values when predicting selection coefficient (*s*), initial frequency (*f*), and time of selection (*T*_sel_) for YRI and CEU populations when tested with simulations of selective sweeps plus background selection. The values show RMSE and MAE measured between standardized log-scaled predicted and actual parameters in simulated data.
(PDF)

S9 Table Root mean squared error (RMSE) and mean absolute error (MAE) values when predicting selection coefficient (*s*), initial frequency (*f*), and time of selection (*T*_sel_) for YRI and CEU populations when tested with simulations of selective sweeps plus background selection. The values show RMSE and MAE measured between log-scaled predicted and actual parameters after unstandardizing.
(PDF)

S10 Table Root mean squared error (RMSE) and mean absolute error (MAE) values when predicting selection coefficient (*s*), initial frequency (*f*), and time of selection (*T*_sel_) for CEU populations when trained and tested with simulations sampling *n* = 20, 50, or 200 haploid genomes. The values show RMSE and MAE measured between standardized log-scaled predicted and actual parameters.
(PDF)

S11 Table Root mean squared error (RMSE) and mean absolute error (MAE) values when predicting selection coefficient (*s*), initial frequency (*f*), and time of selection (*T*_sel_) for CEU populations when trained and tested with simulations using recombination rate drawn from an exponential distribution with mean 10^−8^ truncated at three times the mean per site per generation or rate drawn from an empirical human recombination map. The values show RMSE and MAE measured between standardized log-scaled predicted and actual parameters.
(PDF)

S12 Table Root mean squared error (RMSE) and mean absolute error (MAE) values when predicting selection coefficient (*s*), initial frequency (*f*), and time of selection (*T*_sel_) for YRI and CEU populations when tested with simulations of selective sweeps with *f* ∈ [0.1, 0.2]. The values show RMSE and MAE measured between standardized log-scaled predicted and actual parameters.
(PDF)

S13 Table Root mean squared error (RMSE) and mean absolute error (MAE) values when predicting selection coefficient (*s*), initial frequency (*f*), and time of donor-recipient split (*T*_split_) under adaptive introgression scenarios for YRI and CEU populations. The values show RMSE and MAE measured between standardized log-scaled predicted and actual parameters in simulated data.
(PDF)

S14 Table Classification of CEU data with classifier trained to differentiate sweeps and neutrality, *γ* = 1, Level 1 chosen through cross validation (see *Training the models*), Daubechies’ least asymmetic wavelets. (PDF)

S15 Table Classification of YRI data with classifier trained to differentiate sweeps and neutrality, *γ* = 1, Level 1 chosen through cross validation (see *Training the models*), Daubechies’ least asymmetic wavelets. (PDF)

S16 Table Classification of CEU data with classifier trained to differentiate adaptive introgression, sweeps, and neutrality, *γ* = 1, Level 1 chosen through cross validation (see *Training the models*), Daubechies’ least asymmetic wavelets. (PDF)

S17 Table Classification of CEU data with classifier trained to differentiate adaptive introgression, sweeps, and neutrality, *γ* = 1, Level 1 chosen through cross validation (see *Training the models*), Daubechies’ least asymmetic wavelets, including two-dimensional statistics. (PDF)

S18 Table Classification of YRI data with classifier trained to differentiate adaptive introgression, sweeps, and neutrality, *γ* = 1, Level 1 chosen through cross validation (see *Training the models*), Daubechies’ least asymmetic wavelets. (PDF)

S19 Table Classification of YRI data with classifier trained to differentiate adaptive introgression, sweeps, and neutrality, *γ* = 1, Level 1 chosen through cross validation (see *Training the models*), Daubechies’ least asymmetic wavelets, including two-dimensinoal statistics. (PDF)

S20 Table Runtime comparison when training *SURFDAWave* (Daubechies’ least-asymmetric wavelets), *Trendsetter* (linear trend filtering), diploS/HIC, and evolBoosting with 5000 simulations each when differentiating between sweeps and neutrality. All estimates assume that feature vectors have already been computed for each method.
(PDF)

S1 FigReconstructed wavelets from regression coefficients (*β*s) in sweep versus neutrality scenarios for summary statistics $\hat{\pi }$, *H*_1_, *H*_12_, *H*_2_/*H*_1_, and frequencies of first to fifth most common haplotypes for *SURFDAWave* when *γ* = 1.*SURFDAWave* was trained on simulations of scenarios simulated under demographic specifications for European CEU demographic history. Note that the wavelet reconstructions for all summary statistics are plotted on the same scale, thereby making the distributions of some summaries difficult to decipher as their magnitudes are relatively small. *SURFDAWave* results shown are using Daubechies’ least-asymmetric wavelets to estimate spatial distributions of summary statistics. Level 1 chosen through cross validation.(PDF)

S2 Fig Spatial distribution of regression coefficients (*β*s) in sweep scenarios for summary statistics *H*_1_, *H*_12_, *H*_2_/*H*_1_, and frequencies of first to sixth most common haplotypes for *Trendsetter* with a linear *d* = 2 trend penalty. *Trendsetter* was trained on simulations of constant demographic history.
(PDF)

S3 Fig *SURFDAWave* classifier performance on simulated data. (Left column) Power to differentiate between sweep and neutrality by comparing the probability of a sweep under sweep simulations with the same probability in simulations of neutrality when using varying *γ* penalties, wavelet types, and demographic histories. (Top row confusion matrices) Confusion matrices comparing classification rates of *SURFDAWave* when trained and tested with the CEU demographic history when using Daubechies’ least-Asymmetric wavelets to estimate spatial distributions of summary statistics when using either *γ* = 1, *γ* = 0, or *γ* chosen through cross validation (see *Training the models*). (Middle row confusion matrices) Confusion matrices comparing classification rates of *SURFDAWave* when trained and tested with the CEU demographic history when using Haar wavelets to estimate spatial distributions of summary statistics when using either *γ* = 1, *γ* = 0, or *γ* chosen through cross validation. (Bottom) Confusion matrix showing classification rates of *SURFDAWave* when trained and tested with constant demographic history when using Daubechies’ least-Asymmetric wavelets.
(PDF)

S4 FigReconstructed wavelets from regression coefficients (*β*s) in sweep versus neutrality scenarios for summary statistics $\hat{\pi }$, *H*_1_, *H*_12_, *H*_2_/*H*_1_, and frequencies of first to fifth most common haplotypes for *SURFDAWave* when *γ* = 1.*SURFDAWave* was trained on simulations of scenarios simulated under demographic specifications for European CEU demographic history. Note that the wavelet reconstructions for all summary statistics are plotted on the same scale, thereby making the distributions of some summaries difficult to decipher as their magnitudes are relatively small. *SURFDAWave* results shown are using Haar wavelets to estimate spatial distributions of summary statistics. Level 2 chosen through cross validation.(PDF)

S5 FigReconstructed wavelets from regression coefficients (*β*s) in sweep versus neutrality scenarios for summary statistics $\hat{\pi }$, *H*_1_, *H*_12_, *H*_2_/*H*_1_, and frequencies of first to fifth most common haplotypes for *SURFDAWave* when *γ* = 0.*SURFDAWave* was trained on simulations of scenarios simulated under demographic specifications for European CEU demographic history. Note that the wavelet reconstructions for all summary statistics are plotted on the same scale, thereby making the distributions of some summaries difficult to decipher as their magnitudes are relatively small. *SURFDAWave* results shown are using Daubechies’ least-asymmetric wavelets to estimate spatial distributions of summary statistics. Level 4 chosen through cross validation.(PDF)

S6 FigReconstructed wavelets from regression coefficients (*β*s) in sweep versus neutrality scenarios for summary statistics $\hat{\pi }$, *H*_1_, *H*_12_, *H*_2_/*H*_1_, and frequencies of first to fifth most common haplotypes for *SURFDAWave* when *γ* = 0.7.*SURFDAWave* was trained on simulations of scenarios simulated under demographic specifications for European CEU demographic history. Note that the wavelet reconstructions for all summary statistics are plotted on the same scale, thereby making the distributions of some summaries difficult to decipher as their magnitudes are relatively small. *SURFDAWave* results shown are using Daubechies’ least-asymmetric wavelets to estimate spatial distributions of summary statistics. Level 4 and *γ* = 0.7 chosen through cross validation.(PDF)

S7 Fig *SURFDAWave* performance on simulated data trained and tested with simulations conducted with YRI demographic history to differentiate between sweeps and neutrality. *SURFDAWave* parameters using Daubechies’ least-Asymmetric wavelets to estimate spatial distributions of summary statistics and using *γ* = 1 or *γ* = 0. (Left) Power to differentiate between sweep and neutrality by comparing the probability of a sweep under sweep simulations with the same probability in simulations of neutrality when using varying *γ* penalties in *SURFDAWave*. (Right confusion matrices) Classification rates using *SURFDAWave* when using *γ* = 1 and *γ* = 0.
(PDF)

S8 FigReconstructed wavelets from regression coefficients (*β*s) in sweep versus neutrality scenarios for summary statistics $\hat{\pi }$, *H*_1_, *H*_12_, *H*_2_/*H*_1_, and frequencies of first to fifth most common haplotypes for *SURFDAWave* when *γ* = 0.*SURFDAWave* was trained on simulations of scenarios simulated under demographic specifications for sub-Saharan African YRI demographic history. Note that the wavelet reconstructions for all summary statistics are plotted on the same scale, thereby making the distributions of some summaries difficult to decipher as their magnitudes are relatively small. *SURFDAWave* results shown are using Daubechies’ least-asymmetric wavelets to estimate spatial distributions of summary statistics. Level 4 chosen through cross validation.(PDF)

S9 FigReconstructed wavelets from regression coefficients (*β*s) in sweep versus neutrality scenarios for summary statistics $\hat{\pi }$, *H*_1_, *H*_12_, *H*_2_/*H*_1_, and frequencies of first to fifth most common haplotypes for *SURFDAWave* when *γ* = 0.5.*SURFDAWave* was trained on simulations of scenarios simulated under demographic specifications for sub-Saharan African YRI demographic history. Note that the wavelet reconstructions for all summary statistics are plotted on the same scale, thereby making the distributions of some summaries difficult to decipher as their magnitudes are relatively small. *SURFDAWave* results shown are using Daubechies’ least-asymmetric wavelets to estimate spatial distributions of summary statistics. Level 1 and *γ* chosen through cross validation.(PDF)

S10 FigReconstructed wavelets from regression coefficients (*β*s) in sweep versus neutrality scenarios for summary statistics $\hat{\pi }$, *H*_1_, *H*_12_, *H*_2_/*H*_1_, and frequencies of first to fifth most common haplotypes for *SURFDAWave* when *γ* = 1.*SURFDAWave* was trained on simulations of scenarios simulated under demographic specifications for sub-Saharan African YRI demographic history. Note that the wavelet reconstructions for all summary statistics are plotted on the same scale, thereby making the distributions of some summaries difficult to decipher as their magnitudes are relatively small. *SURFDAWave* results shown are using Daubechies’ least-asymmetric wavelets to estimate spatial distributions of summmry statistics. Level 1 chosen through cross validation.(PDF)

S11 Fig Reconstructed wavelets from regression coefficients (*β*s) in sweep vs. neutrality scenarios for summary statistics *H*_1_ and *H*_12_ showing difference between discrete wavelet transform at level 0 and level 5. Using Daubechies’ least-Asymmetric wavelets and *γ* = 1.
(PDF)

S12 FigReconstructed wavelets from regression coefficients (*β*s) when differentiating among adaptive introgression, sweeps, and neutrality scenarios for summary statistics mean, variance, skewness, and kurtosis of pairwise *r*^2^ for *SURFDAWave* when *γ* = 1, when trained with statistics $\hat{\pi }$, *H*_1_, *H*_12_, *H*_2_/*H*_1_, and frequencies of first to fifth most common haplotypes (S14 Fig).*SURFDAWave* was trained on simulations of scenarios simulated under demographic specifications for European CEU demographic history. Note that the wavelet reconstructions for all summary statistics are plotted on the same scale, thereby making the distributions of some summaries difficult to decipher as their magnitudes are relatively small. *SURFDAWave* results shown are using Daubechies’ least-asymmetric wavelets to estimate spatial distributions of summary statistics. Level 1 chosen through cross validation.(PDF)

S13 FigReconstructed wavelets from regression coefficients (*β*s) when differentiating among adaptive introgression, sweeps, and neutrality scenarios for summary statistics $\hat{\pi }$, *H*_1_, *H*_12_, *H*_2_/*H*_1_, and frequencies of first to fifth most common haplotypes for *SURFDAWave* when *γ* = 1.*SURFDAWave* was trained on simulations of scenarios simulated under demographic specifications for European CEU demographic history. Note that the wavelet reconstructions for all summary statistics are plotted on the same scale, thereby making the distributions of some summaries difficult to decipher as their magnitudes are relatively small. *SURFDAWave* results shown are using Daubechies’ least-asymmetric wavelets to estimate spatial distributions of summary statistics. Level 1 chosen through cross validation.(PDF)

S14 FigReconstructed wavelets from regression coefficients (*β*s) when differentiating among adaptive introgression, sweeps, and neutrality scenarios for summary statistics $\hat{\pi }$, *H*_1_, *H*_12_, *H*_2_/*H*_1_, and frequencies of first to fifth most common haplotypes for *SURFDAWave* when *γ* = 1, when trained with additional statistics mean, variance, skewness, and kurtosis of pairwise *r*^2^ (S12 Fig).*SURFDAWave* was trained on simulations of scenarios simulated under demographic specifications for European CEU demographic history. Note that the wavelet reconstructions for all summary statistics are plotted on the same scale, thereby making the distributions of some summaries difficult to decipher as their magnitudes are relatively small. *SURFDAWave* results shown are using Daubechies’ least-asymmetric wavelets to estimate spatial distributions of summary statistics. Level 1 chosen through cross validation.(PDF)

S15 FigReconstructed wavelets from regression coefficients (*β*s) when differentiating among adaptive introgression, sweeps, and neutrality scenarios for summary statistics $\hat{\pi }$, *H*_1_, *H*_12_, *H*_2_/*H*_1_, and frequencies of first to fifth most common haplotypes for *SURFDAWave* when *γ* = 1.*SURFDAWave* was trained on simulations of scenarios simulated under demographic specifications for sub-Saharan African YRI demographic history. Note that the wavelet reconstructions for all summary statistics are plotted on the same scale, thereby making the distributions of some summaries difficult to decipher as their magnitudes are relatively small. *SURFDAWave* results shown are using Daubechies’ least-asymmetric wavelets to estimate spatial distributions of summary statistics. Level 1 chosen through cross validation.(PDF)

S16 FigReconstructed wavelets from regression coefficients (*β*s) when differentiating among adaptive introgression, sweeps, and neutrality scenarios for summary statistics $\hat{\pi }$, *H*_1_, *H*_12_, *H*_2_/*H*_1_, and frequencies of first to fifth most common haplotypes for *SURFDAWave* when *γ* = 1, when trained with additional statistics mean, variance, skewness, and kurtosis of pairwise *r*^2^ (S17 Fig).*SURFDAWave* was trained on simulations of scenarios simulated under demographic specifications for sub-Saharan African YRI demographic history. Note that the wavelet reconstructions for all summary statistics are plotted on the same scale, thereby making the distributions of some summaries difficult to decipher as their magnitudes are relatively small. *SURFDAWave* results shown are using Daubechies’ least-asymmetric wavelets to estimate spatial distributions of summary statistics. Level 1 chosen through cross validation.(PDF)

S17 FigReconstructed wavelets from regression coefficients (*β*s) when differentiating among adaptive introgression, sweeps, and neutrality scenarios for summary statistics mean, variance, skewness, and kurtosis of pairwise *r*^2^ for *SURFDAWave* when *γ* = 1, when trained with statistics $\hat{\pi }$, *H*_1_, *H*_12_, *H*_2_/*H*_1_, and frequencies of first to fifth most common haplotypes (S16 Fig).*SURFDAWave* was trained on simulations of scenarios simulated under demographic specifications for sub-Saharan African YRI demographic history. Note that the wavelet reconstructions for all summary statistics are plotted on the same scale, thereby making the distributions of some summaries difficult to decipher as their magnitudes are relatively small. *SURFDAWave* results shown are using Daubechies’ least-asymmetric wavelets to estimate spatial distributions of summary statistics. Level 1 chosen through cross validation.(PDF)

S18 FigConfusion matrices showing classification results for demographic mis-specification compared to when classifiers are trained with multiple demographic histories rates in *SURFDAWave*, *Trendsetter*, diploS/HIC, and evolBoosting.Summary statistics $\hat{\pi }$, *H*_1_, *H*_12_, *H*_2_/*H*_1_, and frequency of the first, second, third, fourth, and fifth most common haplotypes used by both *Trendsetter* and *SURFDAWave*. *SURFDAWave* results shown are using Daubechies’ least-asymmetric wavelets to estimate spatial distributions of summary statistics when level and *γ* are chosen through cross validation (see *Training the models*). Training data consist of a balanced dataset of simulations conducted under demographic specifications for European (CEU) and African (YRI) human populations when training for multiple demographic histories. (Left) Classification rates of simulations conducted under CEU European demographic specifications when the model is trained with simulations conducted under YRI African demographic specifications. (Middle right) Classification rates of simulations conducted under YRI African demographic specifications when the model is trained with simulations conducted under CEU European demographic specifications. (Middle right) Classification rates of simulations conducted under CEU European demographic specifications. (Right) Classification rates of simulations conducted under YRI African demographic specifications.(PDF)

S19 FigConfusion matrices showing the effect sample size has on classification rates. We train and test *SURFDAWave*, *Trendsetter*, diploS/HIC, and evolBoosting classifiers to differentiate sweeps and neutrality using sample sizes of *n* = 20, 50, and 200 haploid genomes.*SURFDAWave* results shown are using Daubechies’ least-asymmetric wavelets to estimate spatial distributions of summary statistics and *γ* and levels are chosen through cross validation (see *Training the models*). Summary statistics $\hat{\pi }$, *H*_1_, *H*_12_, *H*_2_/*H*_1_, and frequency of the first, second, third, fourth, and fifth most common haplotypes used by both *Trendsetter* and *SURFDAWave*.(PDF)

S20 FigConfusion matrices comparing classification rates when *SURFDAWave*, *Trendsetter*, diploS/HIC, and evolBoosting are trained and tested using simulations conducted under *Drosophila* population parameters to differentiate between sweeps and neutrality.*SURFDAWave* results shown are using Daubechies’ least-asymmetric wavelets to estimate spatial distributions of summary statistics and *γ* and levels are chosen through cross validation (see *Training the models*). Summary statistics $\hat{\pi }$, *H*_1_, *H*_12_, *H*_2_/*H*_1_, and frequency of the first, second, third, fourth, and fifth most common haplotypes used by both *Trendsetter* and *SURFDAWave*.(PDF)

S21 FigConfusion matrices comparing classification rates of *SURFDAWave*, *Trendsetter*, diploS/HIC, and evolBoosting when applied to simulations with a recombination rate drawn from an exponential distribution with mean 10^−8^ per site per generations, truncated at three times the mean (top row) and recombination rate drawn from a human empirical recombination map (bottom row) to differentiate between sweeps and neutrality.All simulations were conducted under European (CEU) demographic history specifications. *SURFDAWave* results shown are using Daubechies’ least-asymmetric wavelets to estimate spatial distributions of summary statistics and *γ* and levels are chosen through cross validation (see *Training the models*). Summary statistics $\hat{\pi }$, *H*_1_, *H*_12_, *H*_2_/*H*_1_, and frequency of the first, second, third, fourth, and fifth most common haplotypes used by both *Trendsetter* and *SURFDAWave*.(PDF)

S22 Fig Difference between standardized predicted and actual selection parameters with *SURFDAWave* for the CEU and YRI demographic models. (Left box plot) Difference in prediction and truth of log scaled time at which mutation became beneficial. (Middle box plot) Difference in prediction and truth of log scaled frequency reached by mutation prior to it becoming beneficial (*f*). (Right box plot) Difference in prediction and truth of log scaled selection coefficient (*s*).
(PDF)

S23 FigReconstructed wavelets from regression coefficients (*β*s) in predicting time at which mutation became beneficial, frequency reached by mutation before becoming beneficial, and selection strength for summary statistics $\hat{\pi }$, *H*_1_, *H*_12_, *H*_2_/*H*_1_, and frequencies of first to fifth most common haplotypes for *SURFDAWave* when *γ* = 0.6.*SURFDAWave* was trained on simulations of scenarios simulated under demographic specifications for European CEU demographic history. Note that the wavelet reconstructions for all summary statistics are plotted on the same scale, thereby making the distributions of some summaries difficult to decipher as their magnitudes are relatively small. *SURFDAWave* results shown are using Daubechies’ least-asymmetric wavelets to estimate spatial distributions of summary statistics. Level 1 and *γ* = 0.6 chosen through cross validation.(PDF)

S24 FigReconstructed wavelets from regression coefficients (*β*s) in predicting time at which mutation became beneficial, frequency reached by mutation before becoming beneficial, and selection strength for summary statistics $\hat{\pi }$, *H*_1_, *H*_12_, *H*_2_/*H*_1_, and frequencies of first to fifth most common haplotypes for *SURFDAWave* when *γ* = 0.7.*SURFDAWave* was trained on simulations of scenarios simulated under demographic specifications for sub-Sarahan African YRI demographic history. Note that the wavelet reconstructions for all summary statistics are plotted on the same scale, thereby making the distributions of some summaries difficult to decipher as their magnitudes are relatively small. *SURFDAWave* results shown are using Daubechies’ least-asymmetric wavelets to estimate spatial distributions of summary statistics. Level 0 and *γ* = 0.7 chosen through cross validation.(PDF)

S25 Fig Difference between standardized predicted and actual selection parameters with *SURFDAWave* under several confounding scenarios. Difference in prediction and truth of time at which mutation became beneficial, difference in prediction and truth of log scaled frequency reached by mutation prior to it becoming beneficial (*f*), and difference in prediction and truth of log scaled selection coefficient (*s*) shown as set of three box plots. (Top row) Parameter prediction when training and testing sample sizes are *n* = 20, 50 or 200 shown for the CEU demographic history. (Row two) Parameter prediction when recombination rate is drawn from an exponential distribution with mean 10^−8^ per site per generation, truncated at three times the mean or when recombination is drawn from a human empirical recombination map using CEU demographic history. (Row three) Parameter prediction when testing range for initial frequency is *f* ∈ [0.1, 0.2], which falls outside of training range for CEU and YRI demographic histories. (Bottom row) Parameter prediction when training data is a balanced dataset containing simulations from both CEU and YRI demographic histories and is tested under the specified demographic history.
(PDF)

S26 Fig Difference between standardized predicted and actual selection parameters with *SURFDAWave* for the CEU and YRI demographic models. (Left box plot) Difference in prediction and truth of log scaled time at which donor and recipient populations split. (Middle box plot) Difference in prediction and truth of log scaled frequency reached by mutation prior to it becoming beneficial (*f*). (Right box plot) Difference in prediction and truth of log scaled selection coefficient (*s*).
(PDF)

S27 Fig Reliability diagrams showing how close our predicted probabilities are to actual probabilities. For each classifier we predict the probability of sweep for test 1000 simulations. We divide the predicted probabilities into 950 overlapping windows each of length 0.5, with the first window beginning ranging from 0 to 0.05 and the second from 0.001 to 0.051 and so on with the last window ranging from 0.95 to 1.0. Using these ranges as thresholds, we calculate the mean probability of all predicted probabilities within this range (Mean Prediction) along with the fraction of these cases that are classified as sweep (Observed Fraction).
(PDF)

S28 Fig Predicted selection parameters for all genes in YRI and CEU with probability of being classified as sweep greater than 0.7 (Left) Scatter plot of predicted initial frequency mutation reached before becoming beneficial (Initial frequency) versus generations before present at which selection began (Time of selection). (Middle) Scatter plot of Selection coefficient versus Time of selection. (Right) Scatter plot of Initial frequency versus selection coefficient.
(PDF)

S29 Fig Predicted selection parameters for all genes in YRI (orange) and CEU (blue) with probability of being classified as sweep greater than 0.5 divided into bins of probability of sweep (Left) Predicted number of generations before present at which selection began (Time of selection) as a function of the probability of sweep. (Middle) Frequency reached by mutation before becoming beneficial (*f*) as a function of probability of sweep. (Right) Selection coefficient (*s*) as a function of probability of sweep.
(PDF)

S30 Fig *SURFDAWave* classifier’s application to empirical data for CEU to detect adaptive introgression. Probability of adaptive introgression across the genomic region of labeled chromosome containing the genes of interest. *SURFDAWave* is trained to differentiate among selective sweeps, adaptive introgression, and neutrality with simulations conducted under demographic specifications of the CEU demographic history. The black dots show the predicted probability of adaptive introgression and the gray bars show the positions of the labeled genes. Gaps between black dots are the result of filtering low quality genomic regions (see *Application of empirical data*), such that no SNPs exist in these regions and can therefore not be classified (see S33 Fig as an example of how we classify a SNP spanned by our feature vector).
(PDF)

S31 Fig Sum of squared differences between the empirical CEU and the simulated neutral Terhorst normalized minor allele frequency spectra conditional on removing all minor allele classes with k or fewer minor alleles. (PDF)

S32 Fig Confusion matrices comparing classification rates of *SURFDAWave* differentiating among adaptive introgression, sweeps, and neutrality when simulated under a constant-size demographic model with non-adaptive introgression with 1000, 3000, 5000, or 7000 training samples per class. *SURFDAWave* results shown are using Daubechies’ least-asymmetric wavelets to estimate spatial distributions of summary statistics. Level and *gamma* chosen through cross validation.
(PDF)

S33 Fig Schematic illustrating windows for which summary statistics are calculated in our implementation of *SURFDAWave*. Each bold black line underlines one of the eight 10-SNP long windows. Here we show a sample of six haplotypes (rows) across a string of SNPs (columns) for which we calculate summary statistics in *p* = 8 windows. Summary statistics are calculated for each 10-SNP window, with windows overlapping with each neighbor for five SNPs. The central SNP is taken to be the putative selected site and is located in the overlap of windows four and five. Here we have underlined the alternating windows used to calculate the two-dimensional statistics in red.
(PDF)

S34 Fig Distribution of selection parameters for simulations of sweeps conducted with demographic history parameters of CEU (top row) and YRI (bottom row). (Left column) Distribution of time at which tracked mutation becomes beneficial (reaches initial frequency) in simulations of selective sweeps. (Middle column) Distribution of log-scaled initial frequency (input parameter) reached by mutation before becoming beneficial in simulations of selective sweeps. (Right column) Distribution of log-scaled selection coefficient in simulations of selective sweep.
(PDF)

## References

[1] Riley MA. Positive selection for colicin diversity in bacteria. Molecular Biology and Evolution. 1993;10:1048–1059. 8412648 10.1093/oxfordjournals.molbev.a040054

[2] Suo C, Xu H, Khor CC, Ong RT, Sim X, Chen J, et al Natural positive selection and north-south genetic diversity in East Asia. European Journal of Human Genetics. 2012;20:102–110. 10.1038/ejhg.2011.139 21792231 PMC3234507

[3] Maynard Smith J, Haigh J. The hitch-hiking effect of a favourable gene. Genetical Research. 1974;23:23–35. 10.1017/S0016672300014634 4407212

[4] Setter D, Mousset S, Cheng X, Nielsen R, DeGiorgio M, Hermisson J. VolcanoFinder: genomic scans for adaptive introgression. bioRxiv. 2019. 10.1371/journal.pgen.1008867 PMC7326285 32555579

[5] Schrider DR, Kern AD. S/HIC: robust identification of soft and hard sweeps using machine learning. PLoS Genetics. 2016;12:1–31. 10.1371/journal.pgen.1005928 PMC4792382 26977894

[6] Kern AD, Schrider DR. diploS/HIC: An Updated Approach to Classifying Selective Sweeps. G3: Genes, Genomes, Genetics. 2018 10.1534/g3.118.200262 PMC5982824 29626082

[7] Flagel L, Brandvain Y, Schrider DR. The Unreasonable Effectiveness of Convolutional Neural Networks in Population Genetic Inference. Molecular Biology and Evolution. 2019;36 10.1093/molbev/msy224 30517664 PMC6367976

[8] Chan J, Perrone V, Spence JP, Jenkins PA, Mathieson S, Song YS. A Likelihood-free Inference Framework for Population Genetic Data Using Exchangeable Neural Networks. In: Proceedings of the 32Nd International Conference on Neural Information Processing Systems; 2018. p. 8603–8614. PMC7687905 33244210

[9] Mughal MR, DeGiorgio M. Localizing and classifying selective sweeps with trend filtered regression. Molecular Biology and Evolution. 2019;36:2 10.1093/molbev/msy205 PMC6409434 30398642

[10] Cremona MA, Reimherr M, Chiaromonte F, Xu H, Makova KD, Madrigal P. Functional data analysis for computational biology. Bioinformatics. 2019 10.1093/bioinformatics/btz045 30668667 PMC6736445

[11] Ramsay JO, Silverman BW. Functional Data Analysis. 2nd ed New York, NY: Springer; 2005.

[12] Wang JL, Chiou JM, Müller HG. Functional Data Analysis. Annual Review of Statistics and Its Application. 2016;3:257–295. 10.1146/annurev-statistics-041715-033624

[13] Malaspinas AS, Malaspinas O, Evans SN, Slatkin M. Estimating Allele Age and Selection Coefficient from Time-Serial Data. Genetics. 2012;192(2):599–607. 10.1534/genetics.112.140939 22851647 PMC3454883

[14] Mathieson I, Lazaridis I, Rohland N, Mallick S, Patterson N, Roodenberg SA, et al Genome-wide patterns of selection in 230 ancient Eurasians. Nature. 2015;528:499–503. 10.1038/nature16152 26595274 PMC4918750

[15] Tyler J, Pe’er I. Inference of Population Structure from Time-Series Genotype Data. The American Journal of Human Genetics. 2019;105:317–333. 10.1016/j.ajhg.2019.06.002 31256878 PMC6698887

[16] Prentice HC, Lonn M, Rosquint G, Ihse M, Kindstron M. Gene diversity in a fragmented population of Briza media: grassland continuity in a landscape context. Journal of Ecology. 2006;94:87–97. 10.1111/j.1365-2745.2005.01054.x

[17] Yang J, Qian ZQ, Liu ZL, Li S, Sun GL, Zhao GF. Genetic diversity and geographical differentiation of Dipteronia Oliv. (Aceraceae) endemic to China as revealed by AFLP analysis. Biochemical Systematics and Ecology. 2007;35:593–599. 10.1016/j.bse.2007.03.022

[18] Morente-Lopez J, Garcia C, Lara-Romero C, Garcia-Fernandez A, Draper D, Iriondo JM. Geography and Environment Shape Landscape Genetics of Mediterranean Alpine Species Silene ciliata Poiret. Frontiers in plant science. 2018;9:1698–1698. 10.3389/fpls.2018.01698 30538712 PMC6277476

[19] Lin K, Li H, Schlötterer C, Futschik A. Distinguishing Positive Selection From Neutral Evolution: Boosting the Performance of Summary Statistics. Genetics. 2011;187:229–244. 10.1534/genetics.110.122614 21041556 PMC3018323

[20] Terhorst J, Kamm JA, Song YS. Robust and scalable inference of population history from hundreds of unphased whole-genomes. Nature Genetics. 2017;49:303–309. 10.1038/ng.3748 28024154 PMC5470542

[21] Haller BC, Messer PW. SLiM 3: Forward Genetic Simulations Beyond the Wright–Fisher Model. Molecular Biology and Evolution. 2019;36:632–637. 10.1093/molbev/msy228 30517680 PMC6389312

[22] Scally A, Durbin R. Revising the human mutation rate: implications for understanding human evolution. Nature Reviews Genetics. 2012;13:745 10.1038/nrg3295 22965354

[23] Zou H, Hastie T. Regularization and variable selection via the elastic net. Journal of the Royal Statistical Society: Series B (Statistical Methodology). 2005;67:301–320. 10.1111/j.1467-9868.2005.00503.x

[24] Hill WG, Robertson AR. Linkage disequilibrium in finite populations. Theoretical and Applied Genetics. 1968;38:226–231. 10.1007/BF01245622 24442307

[25] Mallick S, Gnerre S, Reich D. The difficulty of avoiding false positives in genome scans for natural selection. Genome Research. 2009;19:922–933. 10.1101/gr.086512.108 19411606 PMC2675981

[26] Charlesworth B. Stabilizing Selection, Purifying Selection, and Mutational Bias in Finite Populations. Genetics. 2013;194:955–971. 10.1534/genetics.113.151555 23709636 PMC3730922

[27] Schrider DR, Kern AD. Soft Sweeps Are the Dominant Mode of Adaptation in the Human Genome. Molecular Biology and Evolution. 2017;34:1863–1877. 10.1093/molbev/msx154 28482049 PMC5850737

[28] de Manuel M, Kuhlwilm M, Frandsen P, Sousa VC, Desai T, Prado-Martinez J, et al Chimpanzee genomic diversity reveals ancient admixture with bonobos. Science (New York, NY). 2016;354:477–481. 10.1126/science.aag2602 PMC5546212 27789843

[29] Duchen P, Živković D, Hutter S, Stephan W, Laurent S. Demographic Inference Reveals African and European Admixture in the North American Drosophila melanogaster Population. Genetics. 2013;193:291–301. 10.1534/genetics.112.145912 23150605 PMC3527251

[30] Harris RB, Sackman A, Jensen JD. On the unfounded enthusiasm for soft selective sweeps II: Examining recent evidence from humans, flies, and viruses. PLOS Genetics. 2018;14:1–21. 10.1371/journal.pgen.1007859 PMC6336318 30592709

[31] Harris AM, DeGiorgio M. A likelihood approach for uncovering selective sweep signatures from haplotype data. Molecular Biology and Evolution. 2020 10.1093/molbev/msaa115 PMC7530616 32392293

[32] The 1000 Genomes Project Consortium. A global reference for human genetic variation. Nature. 2015;526:68–74. 10.1038/nature15393 26432245 PMC4750478

[33] Voight BF, Kudaravalli S, Wen X, Pritchard JK. A Map of Recent Positive Selection in the Human Genome. PLOS Biology. 2006;4:e72 10.1371/journal.pbio.0040072 16494531 PMC1382018

[34] Bersaglieri T, Sabeti PC, Patterson N, Vanderploeg T, Schaffner SF, Drake JA, et al Genetic Signatures of Strong Recent Positive Selection at the Lactase Gene. The American Journal of Human Genetics. 2004;74:1111–1120. 10.1086/421051 15114531 PMC1182075

[35] Wilde S, Timpson A, Kirsanow K, Kaiser E, Kayser M, Unterländer M, et al Direct evidence for positive selection of skin, hair, and eye pigmentation in Europeans during the last 5,000 y. Proceedings of the National Academy of Sciences of the United States of America. 2014;111:4832–4837. 10.1073/pnas.1316513111 24616518 PMC3977302

[36] Sulem P, Gudbjartsson DF, Stacey SN, Helgason A, Rafnar T, Magnusson KP, et al Genetic determinants of hair, eye and skin pigmentation in Europeans. Nature Genetics. 2007;39:1443 EP–. 10.1038/ng.2007.13 17952075

[37] Harris AM, Garud NR, DeGiorgio M. Detection and Classification of Hard and Soft Sweeps from Unphased Genotypes by Multilocus Genotype Identity. Genetics. 2018;210:1429–1452. 10.1534/genetics.118.301502 30315068 PMC6283157

[38] Fagny M, Patin E, Enard D, Barreiro LB, Quintana-Murci L, Laval G. Exploring the Occurrence of Classic Selective Sweeps in Humans Using Whole-Genome Sequencing Data Sets. Molecular Biology and Evolution. 2014;31:1850–1868. 10.1093/molbev/msu118 24694833

[39] Pickrell JK, Coop G, Novembre J, Kudaravalli S, Li JZ, Absher D, et al Signals of recent positive selection in a worldwide sample of human populations. Genome Research. 2009;19:826–837. 10.1101/gr.087577.108 19307593 PMC2675971

[40] Brilliant HM. The Mouse p (pink-eyed dilution) and Human P Genes, Oculocutaneous Albinism Type 2 (OCA2), and Melanosomal pH. Pigment Cell Research. 2001;14:86–93. 10.1034/j.1600-0749.2001.140203.x 11310796

[41] Zhu G, Evans DM, Duffy DL, Montgomery GW, Medland SE, Gillespie NA, et al A Genome Scan for Eye Color in 502 Twin Families: Most Variation is due to a QTL on Chromosome 15q. Twin Research. 2004;7:197–210. 10.1375/136905204323016186 15169604

[42] Eiberg H, Troelsen J, Nielsen M, Mikkelsen A, Mengel-From J, Kjaer KW, et al Blue eye color in humans may be caused by a perfectly associated founder mutation in a regulatory element located within the HERC2 gene inhibiting OCA2 expression. Human Genetics. 2008;123:177–187. 10.1007/s00439-007-0460-x 18172690

[43] Hublin JJ. The earliest modern human colonization of Europe. Proceedings of the National Academy of Sciences. 2012;109:13471–13472. 10.1073/pnas.1211082109 PMC3427117 22864912

[44] Cook AL, Chen W, Thurber AE, Smit DJ, Smith AG, Bladen TG, et al Analysis of Cultured Human Melanocytes Based on Polymorphisms within the SLC45A2/MATP, SLC24A5/NCKX5, and OCA2/P Loci. Journal of Investigative Dermatology. 2009;129:392–405. 10.1038/jid.2008.211 18650849

[45] Li CY, Zhan YQ, Xu CW, Xu WX, Wang SY, Lv J, et al EDAG regulates the proliferation and differentiation of hematopoietic cells and resists cell apoptosis through the activation of nuclear factor-kB. Cell Death & Differentiation. 2004;11:1299–1308. 10.1038/sj.cdd.4401490 15332117

[46] Baker K, Gordon SL, Melland H, Bumbak F, Scott DJ, Jiang TJ, et al SYT1-associated neurodevelopmental disorder: a case series. Brain. 2018;141:2576–2591. 10.1093/brain/awy209 30107533 PMC6113648

[47] Uhlén M, Fagerberg L, Hallström BM, Lindskog C, Oksvold P, Mardinoglu A, et al Tissue-based map of the human proteome. Science. 2015;347 25613900 10.1126/science.1260419

[48] Vilariño-Güell C, Wider C, Ross O, Dachsel J, Kachergus J, Lincoln S, et al VPS35 Mutations in Parkinson Disease. The American Journal of Human Genetics. 2011;89:162–167. 10.1016/j.ajhg.2011.06.001 21763482 PMC3135796

[49] Bronson PG, Mack SJ, Erlich HA, Slatkin M. A sequence-based approach demonstrates that balancing selection in classical human leukocyte antigen (HLA) loci is asymmetric. Human Molecular Genetics. 2012;22:252–261. 10.1093/hmg/dds424 23065702 PMC3526157

[50] Sankararaman S, Mallick S, Dannemann M, Prüfer K, Kelso J, Pääbo S, et al The genomic landscape of Neanderthal ancestry in present-day humans. Nature. 2014;507:354–357. 10.1038/nature12961 24476815 PMC4072735

[51] Racimo F, Sankararaman S, Nielsen R, Huerta-Sánchez E. Evidence for archaic adaptive introgression in humans. Nature Reviews Genetics. 2015;16:359 EP–. 10.1038/nrg3936 25963373 PMC4478293

[52] Visser M, Palstra RJ, Kayser M. Human skin color is influenced by an intergenic DNA polymorphism regulating transcription of the nearby BNC2 pigmentation gene. Human Molecular Genetics. 2014;23:5750–5762. 10.1093/hmg/ddu289 24916375

[53] Monajemi H, Fontijn RD, Pannekoek H, Horrevoets AJG. The Apolipoprotein L Gene Cluster Has Emerged Recently in Evolution and Is Expressed in Human Vascular Tissue. Genomics. 2002;79:539–546. 10.1006/geno.2002.6729 11944986

[54] DeGiorgio M, Lohmueller KE, Nielsen R. A Model-Based Approach for Identifying Signatures of Ancient Balancing Selection in Genetic Data. PLoS Genetics. 2014;10:1–20. 10.1371/journal.pgen.1004561 PMC4140648 25144706

[55] Siewert KM, Voight BF. Detecting Long-Term Balancing Selection Using Allele Frequency Correlation. Molecular Biology and Evolution. 2017;34:2996–3005. 10.1093/molbev/msx209 28981714 PMC5850717

[56] Bitarello BD, de Filippo C, Teixeira JC, Schmidt JM, Kleinert P, Meyer D, et al Signatures of Long-Term Balancing Selection in Human Genomes. Genome Biology and Evolution. 2018;10:939–955. 10.1093/gbe/evy054 29608730 PMC5952967

[57] Cheng X, DeGiorgio M. Detection of Shared Balancing Selection in the Absence of Trans-Species Polymorphism. Molecular Biology and Evolution. 2018;36:177–199. 10.1093/molbev/msy202 PMC6530816 30380122

[58] Siewert KM, Voight BF. BetaScan2: Standardized statistics to detect balancing selection utilizing substitution data. bioRxiv. 2018. 10.1093/gbe/evaa013 PMC7058154 32011695

[59] Cheng X, DeGiorgio M. Robust and window-insensitive mixture model approaches for localizing balancing selection. bioRxiv. 2019.

[60] Assaf ZJ, Petrov DA, Blundell JR. Obstruction of adaptation in diploids by recessive, strongly deleterious alleles. Proceedings of the National Academy of Sciences. 2015;112:E2658–E2666. 10.1073/pnas.1424949112 PMC4443376 25941393

[61] Adrion JR, Galloway JG, Kern AD. Predicting the Landscape of Recombination Using Deep Learning. Molecular Biology and Evolution. 2020 10.1093/molbev/msaa038 32077950 PMC7253213

[62] Bollback JP, York TL, Nielsen R. Estimation of 2Nes From Temporal Allele Frequency Data. Genetics. 2008;179:497–502. 10.1534/genetics.107.085019 18493066 PMC2390626

[63] Ludwig A, Pruvost M, Reissmann M, Benecke N, Brockmann GA, Castaños P, et al Coat Color Variation at the Beginning of Horse Domestication. Science. 2009;324:485–485. 10.1126/science.1172750 19390039 PMC5102060

[64] Fehren-Schmitz L, Georges L. Ancient DNA reveals selection acting on genes associated with hypoxia response in pre-Columbian Peruvian Highlanders in the last 8500 years. Scientific Reports. 2016;6:23485–. 10.1038/srep23485 26996763 PMC4800713

[65] Schraiber JG, Evans SN, Slatkin M. Bayesian Inference of Natural Selection from Allele Frequency Time Series. Genetics. 2016;203:493–511. 10.1534/genetics.116.187278 27010022 PMC4858794

[66] Loog L, Thomas MG, Barnett R, Allen R, Sykes N, Paxinos PD, et al Inferring Allele Frequency Trajectories from Ancient DNA Indicates That Selection on a Chicken Gene Coincided with Changes in Medieval Husbandry Practices. Molecular Biology and Evolution. 2017;34:1981–1990. 10.1093/molbev/msx142 28444234 PMC5850110

[67] Hernandez RD, Kelley JL, Elyashiv E, Melton SC, Auton A, McVean G, et al Classic Selective Sweeps Were Rare in Recent Human Evolution. Science. 2011;331:920–924. 10.1126/science.1198878 21330547 PMC3669691

[68] Wilson BA, Petrov DA, Messer PW. Soft Selective Sweeps in Complex Demographic Scenarios. Genetics. 2014;198:669–684. 10.1534/genetics.114.165571 25060100 PMC4266194

[69] Chen JM, Cooper DN, Chuzhanova N, Férec C, Patrinos GP. Gene conversion: mechanisms, evolution and human disease. Nature Reviews Genetics. 2007;8:762–775. 10.1038/nrg2193 17846636

[70] Meyer M, Kircher M, Gansauge MT, Li H, Racimo F, Mallick S, et al A High-Coverage Genome Sequence from an Archaic Denisovan Individual. Science. 2012;338:222–226. 10.1126/science.1224344 22936568 PMC3617501

[71] Prüfer K, Racimo F, Patterson N, Jay F, Sankararaman S, Sawyer S, et al The complete genome sequence of a Neanderthal from the Altai Mountains. Nature. 2014;505:43–49. 10.1038/nature12886 24352235 PMC4031459

[72] Bollongino R, Tresset A, Vigne J. Environment and excavation: Pre-lab impacts on ancient DNA analyses. Comptes Rendus Palevol. 2008;7:91–98. 10.1016/j.crpv.2008.02.002

[73] Skov L, Hui R, Shchur V, Hobolth A, Scally A, Schierup MH, et al Detecting archaic introgression using an unadmixed outgroup. PLOS Genetics. 2018;14:1–15. 10.1371/journal.pgen.1007641 PMC6161914 30226838

[74] Hubisz MJ, Williams AL, Siepel A. Mapping gene flow between ancient hominins through demography-aware inference of the ancestral recombination graph. bioRxiv. 2019. 10.1371/journal.pgen.1008895 PMC7410169 32760067

[75] Wall JD, Ratan A, Stawiski E, Wall JD, Stawiski E, Ratan A, et al Identification of African-Specific Admixture between Modern and Archaic Humans. The American Journal of Human Genetics. 2019;105:1254–1261. 10.1016/j.ajhg.2019.11.005 31809748 PMC6904834

[76] Durvasula A, Sankararaman S. Recovering signals of ghost archaic introgression in African populations. Science Advances. 2020;6:1–9. 10.1126/sciadv.aax5097 PMC7015685 32095519

[77] Schrider DR, Ayroles J, Matute DR, Kern AD. Supervised machine learning reveals introgressed loci in the genomes of Drosophila simulans and D. sechellia. PLOS Genetics. 2018 4;14:1–29. 10.1371/journal.pgen.1007341 PMC5933812 29684059

[78] Sugden LA, Atkinson EG, Fischer AP, Rong S, Henn BM, Ramachandran S. Localization of adaptive variants in human genomes using averaged one-dependence estimation. Nature communications. 2018 2;9:703–703. 10.1038/s41467-018-03100-7 29459739 PMC5818606

[79] Sabeti PC, Varilly P, Fry B, Lohmueller J, Hostetter E, Cotsapas C, et al Genome-wide detection and characterization of positive selection in human populations. Nature. 2007;449:913 EP–. 10.1038/nature06250 17943131 PMC2687721

[80] Chen H, Patterson N, Reich D. Population differentiation as a test for selective sweeps. Genome Research. 2010;20:393–402. 10.1101/gr.100545.109 20086244 PMC2840981

[81] Sheehan S, Song YS. Deep Learning for Population Genetic Inference. PLoS Computational Biology. 2016;12:1–28. 10.1371/journal.pcbi.1004845 PMC4809617 27018908

[82] Schrider DR, Kern AD. Discoal: flexible coalescent simulations with selection. Bioinformatics. 2016;32:3839–3841. 10.1093/bioinformatics/btw556 27559153 PMC5167068

[83] Plagnol V, Wall JD. Possible Ancestral Structure in Human Populations. PLOS Genetics. 2006;2:1–8. 10.1371/journal.pgen.0020105 PMC1523253 16895447

[84] Wall JD, Lohmueller KE, Plagnol V. Detecting ancient admixture and estimating demographic parameters in multiple human populations. Molecular biology and evolution. 2009;26:1823–1827. 10.1093/molbev/msp096 19420049 PMC2734152

[85] Vernot B, Akey JM. Resurrecting Surviving Neandertal Lineages from Modern Human Genomes. Science. 2014;343:1017–1021. 10.1126/science.1245938 24476670

[86] Huerta-Sánchez E, Jin X, Asan Bianba Z, Peter BM, Vinckenbosch N, et al Altitude adaptation in Tibetans caused by introgression of Denisovan-like DNA. Nature. 2014;512:194–197. 10.1038/nature13408 25043035 PMC4134395

[87] Racimo F, Gokhman D, Fumagalli M, Ko A, Hansen T, Moltke I, et al Archaic Adaptive Introgression in TBX15/WARS2. Molecular Biology and Evolution. 2016;34:509–524. 10.1093/molbev/msw283 PMC5430617 28007980

[88] Racimo F, Marnetto D, Huerta-Sánchez E. Signatures of Archaic Adaptive Introgression in Present-Day Human Populations. Molecular Biology and Evolution. 2016;34(2):296–317. 10.1093/molbev/msw216 PMC5400396 27756828

[89] Pennings PS, Hermisson J. Soft Sweeps III: The Signature of Positive Selection from Recurrent Mutation. PLOS Genetics. 2006;2:1–15. 10.1371/journal.pgen.0020186 PMC1698945 17173482

[90] Rees JS, Castellano S, Andrés AM. The Genomics of Human Local Adaptation. Trends in Genetics. 2020;36:415–428. 10.1016/j.tig.2020.03.006 32396835

[91] Cybenko G. Approximation by superpositions of a sigmoidal function. Math Control Signal Systems. 1989;2:303–314. 10.1007/BF02551274

[92] Gao W, Makkuva AV, Oh S, Viswanath P. Learning One-hidden-layer Neural Networks under General Input Distributions. In: Proceedings of Machine Learning Research. vol. 89 of Proceedings of Machine Learning Research; 2019. p. 1950–1959.

[93] Daubechies I. Orthonormal wavelets of compactly supported wavelets. Communications on Pure and Applied Mathematics. 1988;41:909–996. 10.1002/cpa.3160410705

[94] Nason GP. Wavelet Methods in Statistics with R. 1st ed New York, NY: Springer; 2008.

[95] Crowley P. An intuitive guide to wavelets for economists. Helsinki, Finland: Bank of Finland research discussion papers; 2005.

[96] Daubechies I. Orthonormal bases of compactly supported wavelets. ommunications on pure and applied math. 1988;11:909–996. 10.1002/cpa.3160410705

[97] Zhao Y, Ogden RT, Reiss PT. Wavelet-based LASSO in functional linear regression. Journal of computational and graphical statistics. 2012;21:600–617. 10.1080/10618600.2012.679241 23794794 PMC3685865

[98] Hazewinkel M. Geometric progression, Encyclopedia of Mathematics. Kluwer Academic Publishers; 2001.

[99] Mousavi SM, Sørensen H. Multinomial functional regression with wavelets and LASSO penalization. Econometrics and Statistics. 2017;1:150–166. 10.1016/j.ecosta.2016.09.005

[100] Friedman J, Hastie T, Tibshirani R. Regularization Paths for Generalized Linear Models via Coordinate Descent. Journal of Statistical Software. 2010;33:1–22. 10.18637/jss.v033.i01 20808728 PMC2929880

[101] Nielsen R, Williamson S, Kim Y, Hubisz MJ, Clark AG, Bustamante C. Genomic scans for selective sweeps using SNP data. Genome research. 2005;15:1566–1575. 10.1101/gr.4252305 16251466 PMC1310644

[102] Takahata N. Allelic genealogy and human evolution. Molecular Biology and Evolution. 1993;10:2–22. 8450756 10.1093/oxfordjournals.molbev.a039995

[103] The International HapMap Consortium. A second generation human haplotype map of over 3.1 million SNPs. Nature. 2007;449(7164):851–861. 10.1038/nature06258 17943122 PMC2689609

[104] Kuhlwilm M, Gronau I, Hubisz MJ, de Filippo C, Prado-Martinez J, Kircher M, et al Ancient gene flow from early modern humans into Eastern Neanderthals. Nature. 2016;530:429 EP–. 10.1038/nature16544 26886800 PMC4933530

[105] Siepel A, Bejerano G, Pedersen JS, Hinrichs AS, Hou M, Rosenbloom K, et al Evolutionarily conserved elements in vertebrate, insect, worm, and yeast genomes. Genome Res. 2005 8;15:1034–1050. 10.1101/gr.3715005 16024819 PMC1182216

[106] Harrow J, Frankish A, Gonzalez JM, Tapanari E, Diekhans M, Kokocinski F, et al GENCODE: the reference human genome annotation for The ENCODE Project. Genome Res. 2012 9;22:1760–1774. 10.1101/gr.135350.111 22955987 PMC3431492

[107] Boyko AR, Williamson SH, Indap AR, Degenhardt JD, Hernandez RD, Lohmueller KE, et al Assessing the Evolutionary Impact of Amino Acid Mutations in the Human Genome. PLoS Genetics. 2008;4:1–13. 10.1371/journal.pgen.1000083 PMC2377339 18516229

[108] Hudson R. Generating samples under a Wright-Fisher neutral model of genetic variation. Bioinformatics. 2002;18:337–338. 10.1093/bioinformatics/18.2.337 11847089

[109] Derrien T, Estellé J, Marco Sola S, Knowles DG, Raineri E, Guigó R, et al Fast Computation and Applications of Genome Mappability. PLoS ONE. 2012;7:1–16. 10.1371/journal.pone.0030377 PMC3261895 22276185

