# Supplementary material for: Learning the properties of adaptive regions with functional data analysis
Source: PLoS Genet. 2020 Aug 27;16(8):e1008896. doi: 10.1371/journal.pgen.1008896 (PMC7480868; doi:10.1371/journal.pgen.1008896)
Supplement: S2 Table — The values show RMSE and MAE measured between log-scaled predicted and actual parameters after unstandardizing. (PDF) [file pgen.1008896.s002.pdf]

Table S2: Root mean squared error (RMSE) and mean absolute error (MAE) values when predicting selection coefficient (s), initial frequency (f), and time of selection (T_{sel}) for YRI and CEU populations. The values show RMSE and MAE measured between log-scaled predicted and actual parameters after unstandardizing.

Population	RMSE(s)	RMSE(f)	RMSE(T_{sel})	MAE(s)	MAE(f)	MAE(T_{sel})
CEU	0.49	0.43	20.34	0.40	0.35	18.02
YRI	0.47	0.45	24.49	0.41	0.38	20.13
