# Supplementary material for: Learning the properties of adaptive regions with functional data analysis
Source: PLoS Genet. 2020 Aug 27;16(8):e1008896. doi: 10.1371/journal.pgen.1008896 (PMC7480868; doi:10.1371/journal.pgen.1008896)
Supplement: S6 Table — The values show RMSE and MAE measured between log-scaled predicted and actual parameters after unstandardizing. (PDF) [file pgen.1008896.s006.pdf]

Table S6: Root mean squared error (RMSE) and mean absolute error (MAE) values when predicting selection coefficient (s), initial frequency (f), and time of selection (T_{sel}) for YRI and CEU populations when tested with models trained with simulations of the opposite demography. The values show RMSE and MAE measured between log-scaled predicted and actual parameters after unstandardizing.

Population	RMSE(s)	RMSE(f)	RMSE(T_{sel})	MAE(s)	MAE(f)	MAE(T_{sel})
CEU	0.50	0.51	26.80	0.44	0.50	20.11
YRI	0.51	0.53	34.86	0.43	0.45	28.49
