# Supplementary material for: Learning the properties of adaptive regions with functional data analysis
Source: PLoS Genet. 2020 Aug 27;16(8):e1008896. doi: 10.1371/journal.pgen.1008896 (PMC7480868; doi:10.1371/journal.pgen.1008896)
Supplement: S7 Table — The values show RMSE and MAE measured between standardized log-scaled predicted and actual parameters. (PDF) [file pgen.1008896.s007.pdf]

Table S7: Root mean squared error (RMSE) and mean absolute error (MAE) values when predicting selection coefficient (s), initial frequency (f), and time of selection (T_{sel}) for CEU and YRI populations when trained with simulations conducted under both YRI and CEU demographic histories and tested with the specified (CEU or YRI) demographic history. The values show RMSE and MAE measured between standardized log-scaled predicted and actual parameters.

Population	RMSE(s)	RMSE(f)	RMSE(T_{sel})	MAE(s)	MAE(f)	MAE(T_{sel})
CEU	0.92	1.00	0.93	0.85	0.86	0.72
YRI	0.95	1.13	1.04	0.86	0.99	0.94
