# Supplementary material for: Learning the properties of adaptive regions with functional data analysis
Source: PLoS Genet. 2020 Aug 27;16(8):e1008896. doi: 10.1371/journal.pgen.1008896 (PMC7480868; doi:10.1371/journal.pgen.1008896)
Supplement: S10 Table — The values show RMSE and MAE measured between standardized log-scaled predicted and actual parameters. (PDF) [file pgen.1008896.s010.pdf]

Table S10: Root mean squared error (RMSE) and mean absolute error (MAE) values when predicting selection coefficient (s), initial frequency (f), and time of selection (T_{sel}) for CEU populations when trained and tested with simulations sampling $n = 20$, 50, or 200 haploid genomes. The values show RMSE and MAE measured between standardized log-scaled predicted and actual parameters.

Number of haploids (n)	RMSE(s)	RMSE(f)	RMSE(T_{sel})	MAE(s)	MAE(f)	MAE(T_{sel})
20	1.00	1.04	0.74	0.82	0.89	0.63
50	0.93	1.01	0.69	0.80	0.83	0.55
200	0.89	0.94	0.67	0.71	0.80	0.54
