# Supplementary material for: Learning the properties of adaptive regions with functional data analysis
Source: PLoS Genet. 2020 Aug 27;16(8):e1008896. doi: 10.1371/journal.pgen.1008896 (PMC7480868; doi:10.1371/journal.pgen.1008896)
Supplement: S11 Table — The values show RMSE and MAE measured between standardized log-scaled predicted and actual parameters. (PDF) [file pgen.1008896.s011.pdf]

Table S11: Root mean squared error (RMSE) and mean absolute error (MAE) values when predicting selection coefficient (s), initial frequency (f), and time of selection (T_{sel}) for CEU populations when trained and tested with simulations using recombination rate drawn from an exponential distribution with mean 10^{-8} truncated at three times the mean per site per generation or rate drawn from an empirical human recombination map. The values show RMSE and MAE measured between standardized log-scaled predicted and actual parameters.

Recombination rate	RMSE(s)	RMSE(f)	RMSE(T_{sel})	MAE(s)	MAE(f)	MAE(T_{sel})
10^{-8}	0.88	1.02	0.69	0.68	0.82	0.51
Human map	0.96	1.11	0.84	0.83	0.93	0.68
