# Supplementary material for: Learning the properties of adaptive regions with functional data analysis
Source: PLoS Genet. 2020 Aug 27;16(8):e1008896. doi: 10.1371/journal.pgen.1008896 (PMC7480868; doi:10.1371/journal.pgen.1008896)
Supplement: S12 Table — The values show RMSE and MAE measured between standardized log-scaled predicted and actual parameters. (PDF) [file pgen.1008896.s012.pdf]

Table S12: Root mean squared error (RMSE) and mean absolute error (MAE) values when predicting selection coefficient (s), initial frequency (f), and time of selection (T_{sel}) for YRI and CEU populations when tested with simulations of selective sweeps with $f \in [0.1, 0.2]$. The values show RMSE and MAE measured between standardized log-scaled predicted and actual parameters.

Population	RMSE(s)	RMSE(f)	RMSE(T_{sel})	MAE(s)	MAE(f)	MAE(T_{sel})
CEU	1.02	1.43	0.81	0.98	1.31	0.79
YRI	1.19	1.32	0.86	1.05	1.24	0.95
