# Supplementary material for: Learning the properties of adaptive regions with functional data analysis
Source: PLoS Genet. 2020 Aug 27;16(8):e1008896. doi: 10.1371/journal.pgen.1008896 (PMC7480868; doi:10.1371/journal.pgen.1008896)
Supplement: S13 Table — The values show RMSE and MAE measured between standardized log-scaled predicted and actual parameters in simulated data. (PDF) [file pgen.1008896.s013.pdf]

Table S13: Root mean squared error (RMSE) and mean absolute error (MAE) values when predicting selection coefficient (s), initial frequency (f), and time of donor-recipient split (T_{split}) under adaptive introgression scenarios for YRI and CEU populations. The values show RMSE and MAE measured between standardized log-scaled predicted and actual parameters in simulated data.

Population	RMSE(s)	RMSE(f)	RMSE(T_{split})	MAE(s)	MAE(f)	MAE(T_{sel})
CEU	0.93	1.00	0.98	0.91	0.96	0.94
YRI	0.97	0.91	0.99	0.93	0.88	0.95
