# Supplementary material for: Learning the properties of adaptive regions with functional data analysis
Source: PLoS Genet. 2020 Aug 27;16(8):e1008896. doi: 10.1371/journal.pgen.1008896 (PMC7480868; doi:10.1371/journal.pgen.1008896)
Supplement: S14 Table — (PDF) [file pgen.1008896.s014.pdf]

Table S14: Classification of CEU data with classifier trained to differentiate sweeps and neutrality, $\gamma = 1$, Level 1 chosen through cross validation (see *Training the models*), Daubechies' least asymmetic wavelets

| Chromosome | Neutral | Sweep | $\mathbb{P}$[Sweep] > 0.7 |
|---|---|---|---|
| 1 | 88.0 | 12.0 | 6.9 |
| 2 | 85.8 | 14.2 | 7.8 |
| 3 | 88.8 | 11.2 | 6.3 |
| 4 | 86.6 | 13.4 | 8.5 |
| 5 | 90.2 | 9.8 | 5.1 |
| 6 | 89.8 | 10.2 | 5.3 |
| 7 | 87.5 | 12.5 | 7.0 |
| 8 | 85.7 | 14.3 | 7.7 |
| 9 | 83.9 | 16.1 | 8.9 |
| 10 | 87.2 | 12.8 | 6.9 |
| 11 | 88.4 | 11.6 | 7.2 |
| 12 | 86.2 | 13.8 | 7.9 |
| 13 | 91.6 | 8.4 | 4.2 |
| 14 | 86.4 | 13.6 | 7.7 |
| 15 | 79.4 | 20.6 | 11.8 |
| 16 | 80.6 | 19.4 | 11.0 |
| 17 | 86.4 | 13.6 | 7.5 |
| 18 | 85.1 | 14.9 | 8.7 |
| 19 | 79.4 | 20.6 | 10.7 |
| 20 | 82.8 | 17.2 | 10.0 |
| 21 | 89.4 | 10.6 | 6.2 |
| 22 | 84.4 | 15.6 | 6.5 |
