# Supplementary material for: Learning the properties of adaptive regions with functional data analysis
Source: PLoS Genet. 2020 Aug 27;16(8):e1008896. doi: 10.1371/journal.pgen.1008896 (PMC7480868; doi:10.1371/journal.pgen.1008896)
Supplement: S15 Table — (PDF) [file pgen.1008896.s015.pdf]

Table S15: Classification of YRI data with classifier trained to differentiate sweeps and neutrality, $\gamma = 1$, Level 1 chosen through cross validation (see *Training the models*), Daubechies' least asymmetic wavelets

| Chromosome | Neutral | Sweep | $\mathbb{P}[\text{Sweep}] > 0.7$ |
|---|---|---|---|
| 1 | 97.1 | 2.9 | 2.0 |
| 2 | 97.8 | 2.2 | 1.4 |
| 3 | 98.2 | 1.8 | 1.1 |
| 4 | 96.6 | 3.4 | 2.0 |
| 5 | 97.2 | 2.8 | 1.7 |
| 6 | 95.2 | 4.8 | 3.4 |
| 7 | 96.1 | 3.9 | 2.5 |
| 8 | 97.5 | 2.5 | 1.7 |
| 9 | 97.2 | 2.8 | 1.9 |
| 10 | 96.9 | 3.1 | 1.9 |
| 11 | 96.7 | 3.3 | 1.8 |
| 12 | 97.4 | 2.6 | 1.9 |
| 13 | 98.2 | 1.8 | 0.9 |
| 14 | 97.2 | 2.8 | 1.3 |
| 15 | 97.9 | 2.1 | 1.3 |
| 16 | 95.8 | 4.2 | 3.0 |
| 17 | 97.7 | 2.3 | 1.5 |
| 18 | 98.2 | 1.8 | 1.1 |
| 19 | 94.3 | 5.7 | 3.8 |
| 20 | 97.5 | 2.5 | 2.0 |
| 21 | 97.6 | 2.4 | 1.2 |
| 22 | 98.5 | 1.5 | 1.1 |
