# Supplementary material for: Learning the properties of adaptive regions with functional data analysis
Source: PLoS Genet. 2020 Aug 27;16(8):e1008896. doi: 10.1371/journal.pgen.1008896 (PMC7480868; doi:10.1371/journal.pgen.1008896)
Supplement: S16 Table — (PDF) [file pgen.1008896.s016.pdf]

Table S16: Classification of CEU data with classifier trained to differentiate adaptive introgression, sweeps, and neutrality, $\gamma = 1$, Level 1 chosen through cross validation (see *Training the models*), Daubechies' least asymmetic wavelets

Chromosome	Neutral	Introgression sweep	Sweep	$\mathbb{P}$[Introgression sweep] > 0.6	$\mathbb{P}$[Sweep] > 0.6
1	87.4	4.0	8.6	0.3	1.6
2	85.7	4.7	9.6	0.4	0.4
3	88.4	3.9	7.8	0.3	0.5
4	85.2	6.3	8.5	0.9	1.1
5	90.2	2.7	7.1	0.1	0.5
6	88.9	3.8	7.4	0.2	0.6
7	87.8	3.1	9.2	0.1	0.6
8	84.4	5.6	10.0	0.2	1.2
9	85.2	3.5	11.2	0.2	0.7
10	86.7	5.1	8.2	0.5	0.7
11	88.5	3.3	8.1	0.1	0.9
12	86.8	3.3	9.9	0.3	0.4
13	91.4	3.3	5.3	0.0	0.3
14	86.9	3.2	9.9	0.0	0.5
15	79.4	5.7	14.9	0.3	1.6
16	81.6	5.0	13.4	0.2	2.1
17	87.0	5.2	7.8	0.2	0.3
18	85.3	5.8	8.9	0.1	0.9
19	82.5	4.0	13.5	0.0	0.9
20	83.6	4.5	11.9	0.0	0.6
21	88.3	6.2	5.6	0.2	0.1
22	86.1	2.9	11.0	0.0	0.1
