# Supplementary material for: Learning the properties of adaptive regions with functional data analysis
Source: PLoS Genet. 2020 Aug 27;16(8):e1008896. doi: 10.1371/journal.pgen.1008896 (PMC7480868; doi:10.1371/journal.pgen.1008896)
Supplement: S17 Table — (PDF) [file pgen.1008896.s017.pdf]

Table S17: Classification of CEU data with classifier trained to differentiate adaptive introgression, sweeps, and neutrality, $\gamma = 1$, Level 1 chosen through cross validation (see *Training the models*), Daubechies' least asymmetic wavelets, including two-dimensional statistics

| Chromosome | Neutral | Introgression sweep | Sweep | $\mathbb{P}$[Introgression sweep] $> 0.6$ | $\mathbb{P}$[Sweep] $> 0.6$ |
|---|---|---|---|---|---|
| 1 | 90.5 | 3.5 | 6.1 | 0.5 | 2.3 |
| 2 | 90.3 | 4.7 | 5.1 | 1.0 | 1.4 |
| 3 | 91.4 | 4.3 | 4.3 | 0.9 | 1.1 |
| 4 | 87.4 | 6.0 | 6.6 | 1.5 | 2.1 |
| 5 | 93.2 | 3.4 | 3.4 | 0.7 | 0.7 |
| 6 | 92.8 | 2.9 | 4.3 | 0.6 | 1.3 |
| 7 | 92.8 | 2.7 | 4.4 | 0.4 | 0.9 |
| 8 | 88.0 | 4.9 | 7.0 | 0.8 | 1.7 |
| 9 | 90.9 | 3.8 | 5.3 | 0.7 | 1.1 |
| 10 | 92.0 | 3.9 | 4.2 | 1.1 | 0.8 |
| 11 | 92.2 | 3.8 | 4.0 | 0.6 | 1.3 |
| 12 | 92.2 | 2.7 | 5.1 | 0.6 | 1.1 |
| 13 | 93.7 | 2.9 | 3.3 | 0.3 | 0.3 |
| 14 | 91.3 | 3.7 | 5.0 | 0.7 | 1.9 |
| 15 | 89.4 | 4.7 | 5.9 | 0.8 | 1.4 |
| 16 | 93.0 | 3.1 | 3.9 | 0.3 | 0.7 |
| 17 | 93.4 | 3.0 | 3.6 | 0.4 | 0.8 |
| 18 | 90.1 | 5.5 | 4.4 | 1.4 | 0.8 |
| 19 | 89.9 | 3.2 | 6.9 | 0.3 | 1.2 |
| 20 | 90.3 | 4.6 | 5.1 | 0.3 | 0.9 |
| 21 | 90.9 | 4.4 | 4.7 | 0.7 | 0.8 |
| 22 | 94.4 | 3.8 | 1.8 | 0.3 | 0.0 |
