# Supplementary material for: Learning the properties of adaptive regions with functional data analysis
Source: PLoS Genet. 2020 Aug 27;16(8):e1008896. doi: 10.1371/journal.pgen.1008896 (PMC7480868; doi:10.1371/journal.pgen.1008896)
Supplement: S18 Table — (PDF) [file pgen.1008896.s018.pdf]

Table S18: Classification of YRI data with classifier trained to differentiate adaptive introgression, sweeps, and neutrality, $\gamma = 1$, Level 1 chosen through cross validation (see *Training the models*), Daubechies' least asymmetic wavelets

Chromosome	Neutral	Introgression sweep	Sweep	$\mathbb{P}$[Introgression sweep] > 0.6	$\mathbb{P}$[Sweep] > 0.6
1	96.9	0.3	2.8	0.1	0.7
2	96.9	0.1	2.9	0.0	0.8
3	97.7	0.3	2.0	0.0	0.6
4	96.3	0.5	3.2	0.2	0.6
5	96.6	0.2	3.2	0.0	1.0
6	95.5	1.8	2.8	0.8	0.5
7	96.1	0.3	3.6	0.0	1.0
8	97.3	0.1	2.7	0.0	0.8
9	96.8	0.3	2.9	0.1	0.5
10	97.0	0.7	2.3	0.0	0.3
11	96.5	0.4	3.1	0.0	0.9
12	97.3	0.2	2.4	0.0	0.7
13	98.2	0.3	1.6	0.0	0.3
14	96.9	0.1	3.0	0.0	0.9
15	97.5	0.0	2.5	0.0	0.9
16	95.4	0.7	3.9	0.2	0.9
17	97.7	0.7	1.6	0.2	0.3
18	97.9	0.6	1.5	0.0	0.4
19	94.8	0.8	4.4	0.0	1.2
20	97.5	0.6	1.9	0.0	0.5
21	98.5	0.4	1.2	0.0	0.0
22	98.3	0.6	1.1	0.0	0.2
