# Supplementary material for: Learning the properties of adaptive regions with functional data analysis
Source: PLoS Genet. 2020 Aug 27;16(8):e1008896. doi: 10.1371/journal.pgen.1008896 (PMC7480868; doi:10.1371/journal.pgen.1008896)
Supplement: S19 Table — (PDF) [file pgen.1008896.s019.pdf]

Table S19: Classification of YRI data with classifier trained to differentiate adaptive introgression, sweeps, and neutrality, $\gamma = 1$, Level 1 chosen through cross validation (see *Training the models*), Daubechies' least asymmetic wavelets, including two-dimensinoal statistics

Chromosome	Neutral	Introgression sweep	Sweep	$\mathbb{P}$[Introgression sweep] > 0.6	$\mathbb{P}$[Sweep] > 0.6
1	95.8	0.5	3.7	0.0	0.8
2	96.9	0.3	2.8	0.0	0.3
3	97.4	0.4	2.2	0.1	0.8
4	96.2	0.9	2.9	0.1	0.4
5	96.7	0.3	2.9	0.0	0.7
6	95.6	2.3	2.1	1.1	0.4
7	96.0	0.9	3.0	0.1	0.5
8	96.4	0.8	2.8	0.1	0.7
9	97.3	0.5	2.2	0.0	0.6
10	97.7	0.9	1.4	0.0	0.4
11	96.7	0.8	2.5	0.1	0.3
12	96.1	0.7	3.2	0.0	0.7
13	98.1	0.7	1.3	0.2	0.3
14	97.8	0.3	1.9	0.0	0.6
15	97.0	0.7	2.3	0.0	0.8
16	96.3	1.1	2.5	0.2	0.5
17	97.8	0.8	1.4	0.2	0.1
18	97.8	0.4	1.7	0.0	0.4
19	94.7	2.0	3.3	0.2	0.7
20	97.2	1.1	1.7	0.0	0.4
21	97.9	0.9	1.2	0.1	0.1
22	98.7	1.0	0.4	0.3	0.0
