# Supplementary material for: Learning the properties of adaptive regions with functional data analysis
Source: PLoS Genet. 2020 Aug 27;16(8):e1008896. doi: 10.1371/journal.pgen.1008896 (PMC7480868; doi:10.1371/journal.pgen.1008896)
Supplement: S20 Table — All estimates assume that feature vectors have already been computed for each method. (PDF) [file pgen.1008896.s020.pdf]

Table S20: Runtime comparison when training *SURFDAWave* (Daubechies' least-asymmetric wavelets), *Trendsetter* (linear trend filtering), diploS/HIC, and evolBoosting with 5000 simulations each when differentiating between sweeps and neutrality. All estimates assume that feature vectors have already been computed for each method.

Classification method	Time
SURFDAWave	1434.72 seconds
Trendsetter	10328.74 seconds
diploS/HIC	187.19 seconds
evolBoosting	14.99 seconds
