# Supplementary material for: Learning the properties of adaptive regions with functional data analysis
Source: PLoS Genet. 2020 Aug 27;16(8):e1008896. doi: 10.1371/journal.pgen.1008896 (PMC7480868; doi:10.1371/journal.pgen.1008896)
Supplement: S2 Fig — Trendsetter was trained on simulations of constant demographic history. (PDF) [file pgen.1008896.s022.pdf]

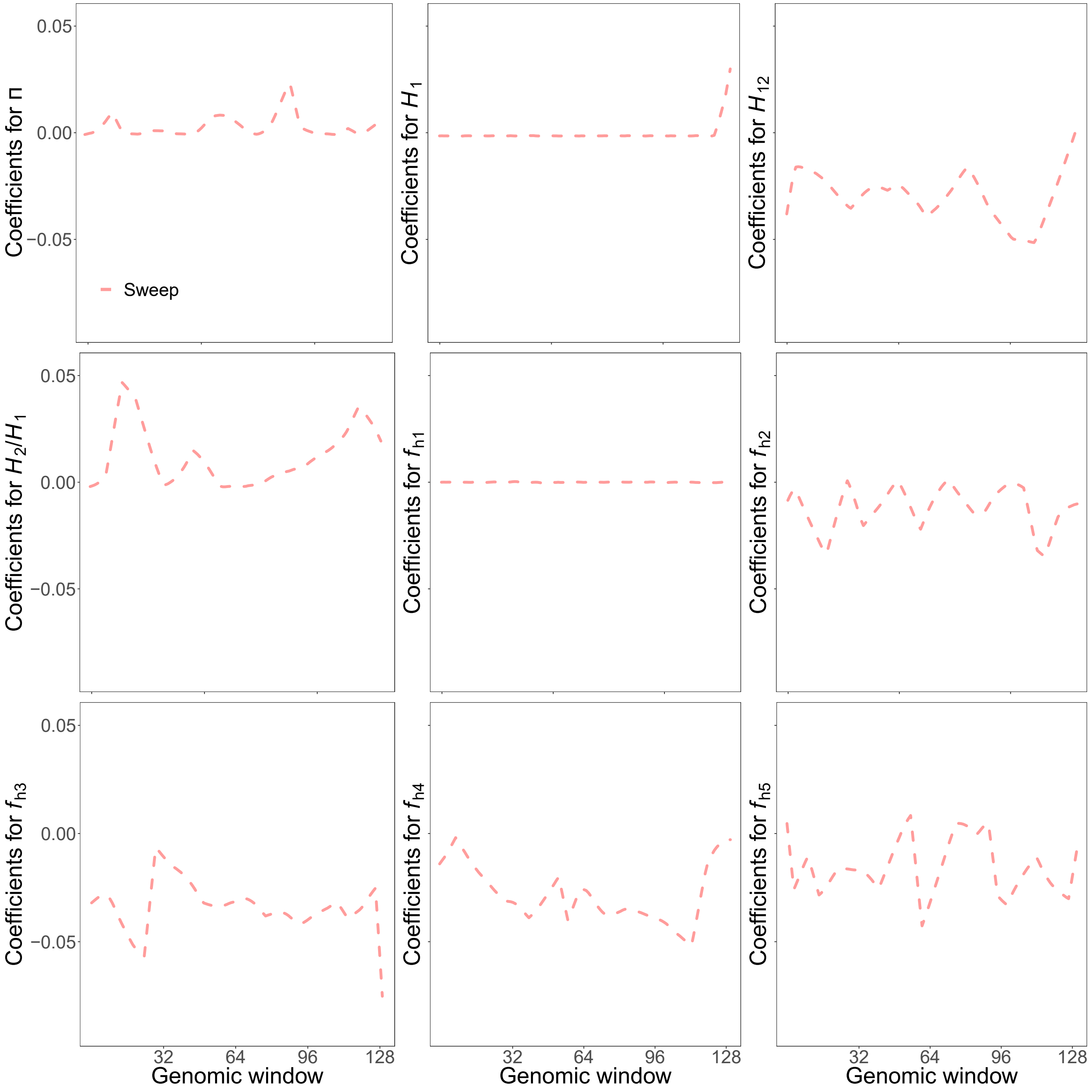

Figure S2: Spatial distribution of regression coefficients (βs) in sweep scenarios for summary statistics H_1, H_{12}, H_2/H_1, and frequencies of first to sixth most common haplotypes for *Trendsetter* with a linear $d = 2$ trend penalty. *Trendsetter* was trained on simulations of constant demographic history.
