# Supplementary material for: Learning the properties of adaptive regions with functional data analysis
Source: PLoS Genet. 2020 Aug 27;16(8):e1008896. doi: 10.1371/journal.pgen.1008896 (PMC7480868; doi:10.1371/journal.pgen.1008896)
Supplement: S3 Fig — (Left column) Power to differentiate between sweep and neutrality by comparing the probability of a sweep under sweep simulations with the same probability in simulations of neutrality when using varying γ penalties, wavelet types, and demographic histories. (Top row confusion matrices) Confusion matrices comparing classification rates of SURFDAWave when trained and tested with the CEU demographic history when using Daubechies’ least-Asymmetric wavelets to estimate spatial distributions of summary statistics when using either γ = 1, γ = 0, or γ chosen through cross validation (see Training the models). (Middle row confusion matrices) Confusion matrices comparing classification rates of SURFDAWave when trained and tested with the CEU demographic history when using Haar wavelets to estimate spatial distributions of summary statistics when using either γ = 1, γ = 0, or γ chosen through cross validation. (Bottom) Confusion matrix showing classification rates of SURFDAWave when trained and tested with constant demographic history when using Daubechies’ least-Asymmetric wavelets. (PDF) [file pgen.1008896.s023.pdf]

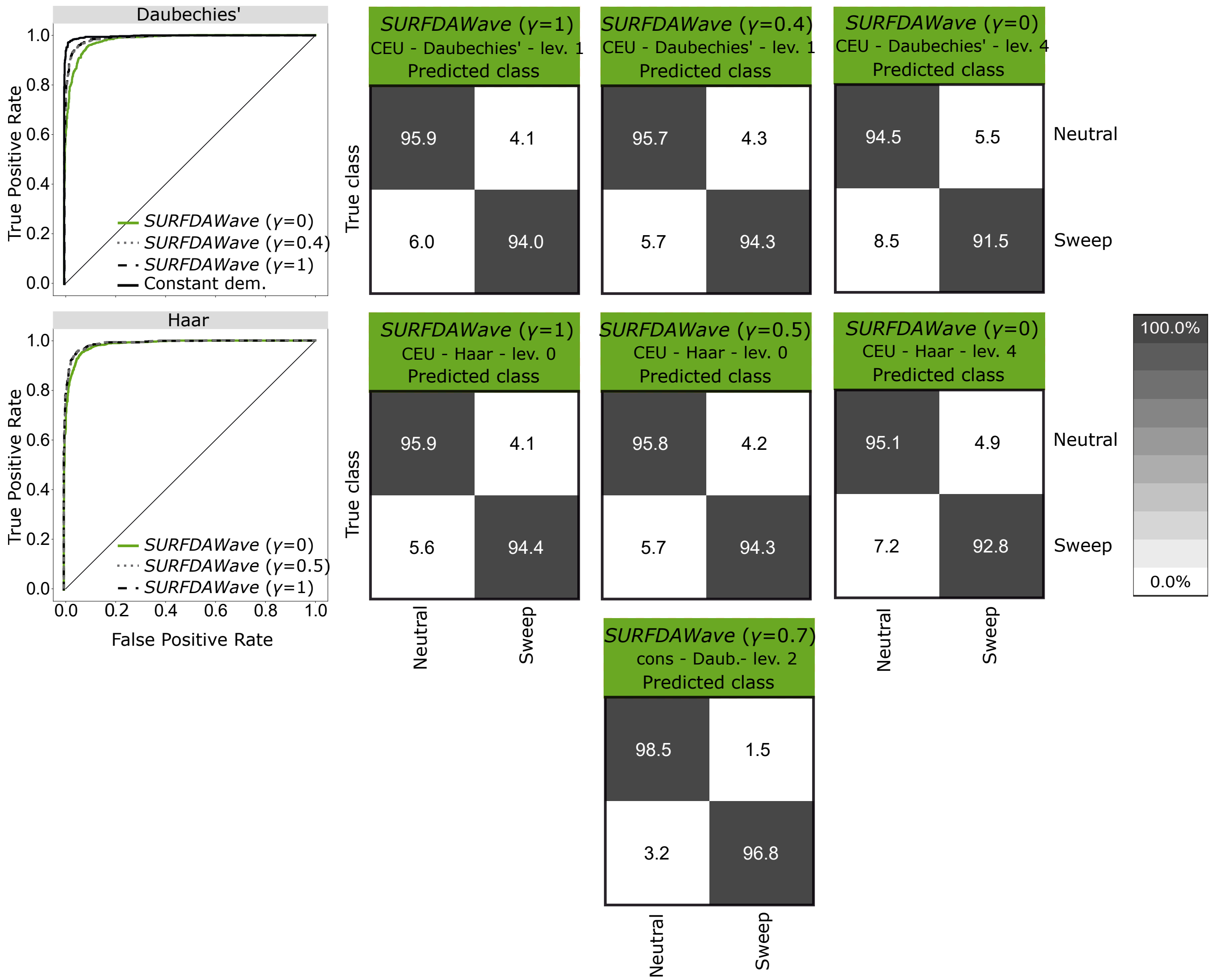

Figure S3: *SURFDAWave* classifier performance on simulated data. (Left column) Power to differentiate between sweep and neutrality by comparing the probability of a sweep under sweep simulations with the same probability in simulations of neutrality when using varying γ penalties, wavelet types, and demographic histories. (Top row confusion matrices) Confusion matrices comparing classification rates of *SURFDAWave* when trained and tested with the CEU demographic history when using Daubechies' least-Asymmetric wavelets to estimate spatial distributions of summary statistics when using either $\gamma = 1$, $\gamma = 0$, or γ chosen through cross validation (see *Training the models*). (Middle row confusion matrices) Confusion matrices comparing classification rates of *SURFDAWave* when trained and tested with the CEU demographic history when using Haar wavelets to estimate spatial distributions of summary statistics when using either $\gamma = 1$, $\gamma = 0$, or γ chosen through cross validation. (Bottom) Confusion matrix showing classification rates of *SURFDAWave* when trained and tested with constant demographic history when using Daubechies' least-Asymmetric wavelets.
