# Supplementary material for: Learning the properties of adaptive regions with functional data analysis
Source: PLoS Genet. 2020 Aug 27;16(8):e1008896. doi: 10.1371/journal.pgen.1008896 (PMC7480868; doi:10.1371/journal.pgen.1008896)
Supplement: S7 Fig — SURFDAWave parameters using Daubechies’ least-Asymmetric wavelets to estimate spatial distributions of summary statistics and using γ = 1 or γ = 0. (Left) Power to differentiate between sweep and neutrality by comparing the probability of a sweep under sweep simulations with the same probability in simulations of neutrality when using varying γ penalties in SURFDAWave. (Right confusion matrices) Classification rates using SURFDAWave when using γ = 1 and γ = 0. (PDF) [file pgen.1008896.s027.pdf]

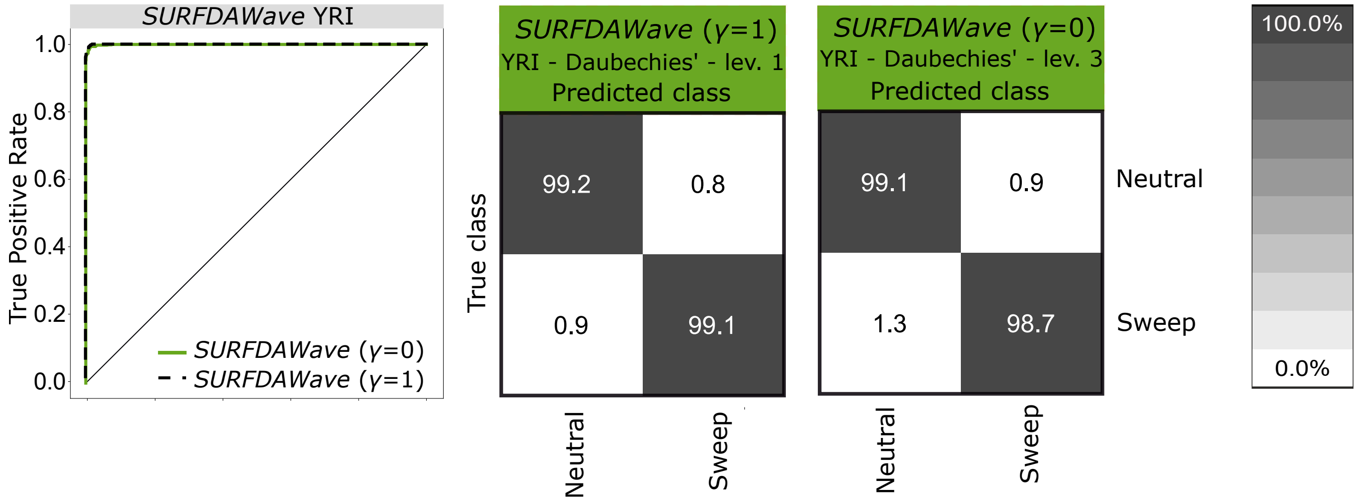

Figure S7: *SURFDAWave* performance on simulated data trained and tested with simulations conducted with YRI demographic history to differentiate between sweeps and neutrality. *SURFDAWave* parameters using Daubechies' least-Asymmetric wavelets to estimate spatial distributions of summary statistics and using $\gamma = 1$ or $\gamma = 0$. (Left) Power to differentiate between sweep and neutrality by comparing the probability of a sweep under sweep simulations with the same probability in simulations of neutrality when using varying γ penalties in *SURFDAWave*. (Right confusion matrices) Classification rates using *SURFDAWave* when using $\gamma = 1$ and $\gamma = 0$.
