# Supplementary material for: Learning the properties of adaptive regions with functional data analysis
Source: PLoS Genet. 2020 Aug 27;16(8):e1008896. doi: 10.1371/journal.pgen.1008896 (PMC7480868; doi:10.1371/journal.pgen.1008896)
Supplement: S9 Fig — SURFDAWave was trained on simulations of scenarios simulated under demographic specifications for sub-Saharan African YRI demographic history. Note that the wavelet reconstructions for all summary statistics are plotted on the same scale, thereby making the distributions of some summaries difficult to decipher as their magnitudes are relatively small. SURFDAWave results shown are using Daubechies’ least-asymmetric wavelets to estimate spatial distributions of summary statistics. Level 1 and γ chosen through cross validation. (PDF) [file pgen.1008896.s029.pdf]

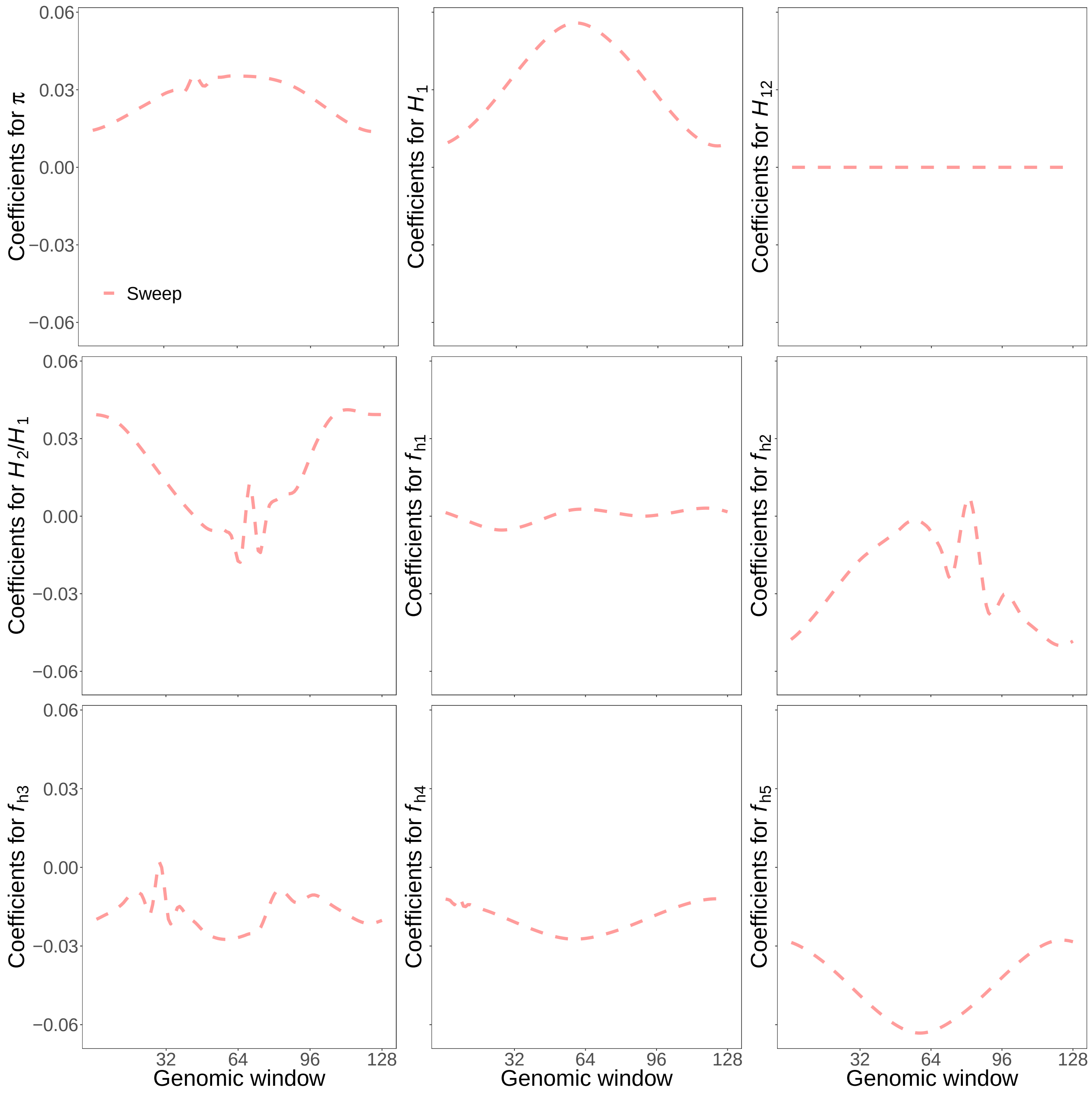

Figure S9: Reconstructed wavelets from regression coefficients (βs) in sweep versus neutrality scenarios for summary statistics $\widehat{\pi}$, H_1, H_{12}, H_2/H_1, and frequencies of first to fifth most common haplotypes for *SURFDAWave* when $\gamma = 0.5$. *SURFDAWave* was trained on simulations of scenarios simulated under demographic specifications for sub-Saharan African YRI demographic history. Note that the wavelet reconstructions for all summary statistics are plotted on the same scale, thereby making the distributions of some summaries difficult to decipher as their magnitudes are relatively small. *SURFDAWave* results shown are using Daubechies' least-asymmetric wavelets to estimate spatial distributions of summary statistics. Level 1 and γ chosen through cross validation.
