# Supplementary material for: Learning the properties of adaptive regions with functional data analysis
Source: PLoS Genet. 2020 Aug 27;16(8):e1008896. doi: 10.1371/journal.pgen.1008896 (PMC7480868; doi:10.1371/journal.pgen.1008896)
Supplement: S11 Fig — Using Daubechies’ least-Asymmetric wavelets and γ = 1. (PDF) [file pgen.1008896.s031.pdf]

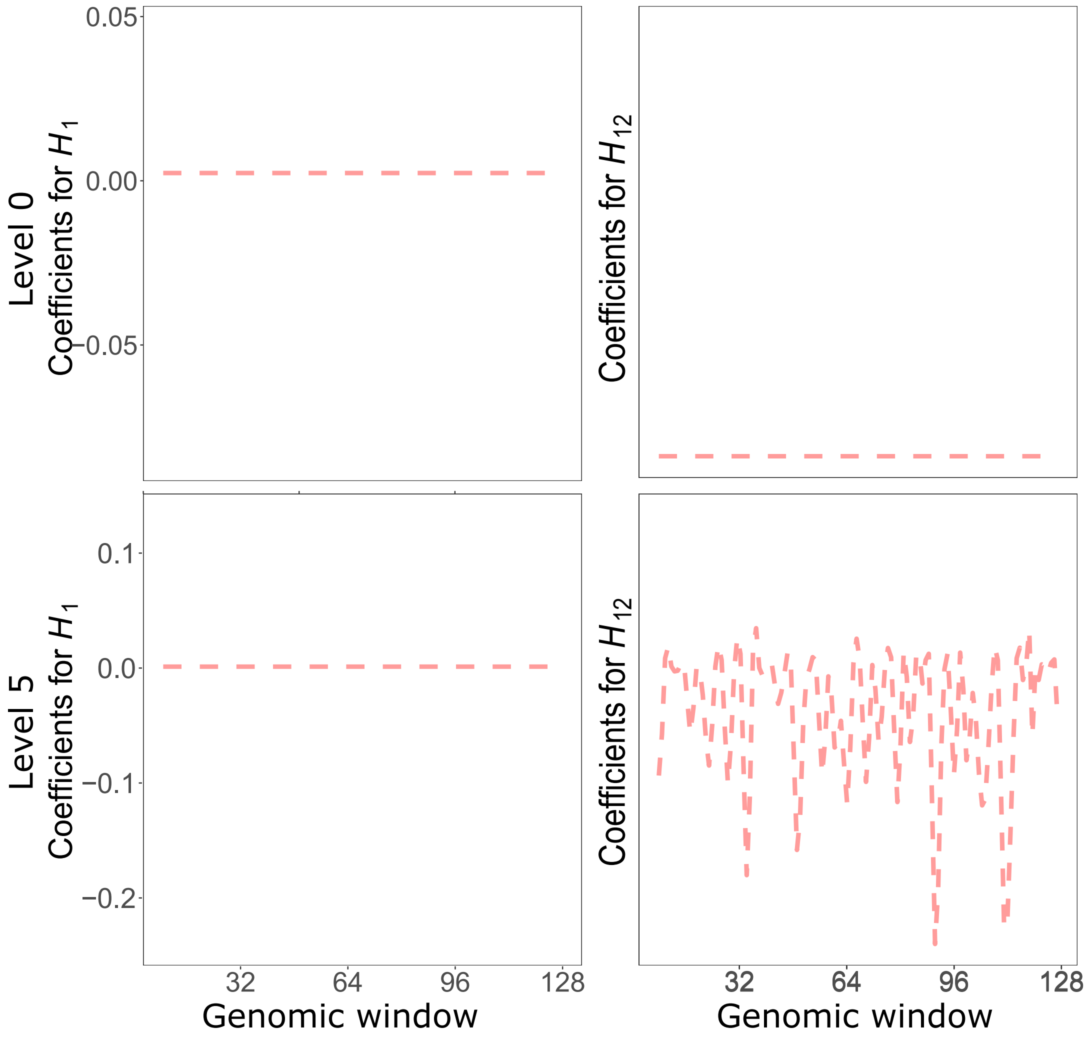

Figure S11: Reconstructed wavelets from regression coefficients (βs) in sweep vs. neutrality scenarios for summary statistics H_1 and H_{12} showing difference between discrete wavelet transform at level 0 and level 5. Using Daubechies' least-Asymmetric wavelets and $\gamma = 1$.
