# Supplementary material for: Learning the properties of adaptive regions with functional data analysis
Source: PLoS Genet. 2020 Aug 27;16(8):e1008896. doi: 10.1371/journal.pgen.1008896 (PMC7480868; doi:10.1371/journal.pgen.1008896)
Supplement: S18 Fig — Summary statistics π^, H1, H12, H2/H1, and frequency of the first, second, third, fourth, and fifth most common haplotypes used by both Trendsetter and SURFDAWave. SURFDAWave results shown are using Daubechies’ least-asymmetric wavelets to estimate spatial distributions of summary statistics when level and γ are chosen through cross validation (see Training the models). Training data consist of a balanced dataset of simulations conducted under demographic specifications for European (CEU) and African (YRI) human populations when training for multiple demographic histories. (Left) Classification rates of simulations conducted under CEU European demographic specifications when the model is trained with simulations conducted under YRI African demographic specifications. (Middle right) Classification rates of simulations conducted under YRI African demographic specifications when the model is trained with simulations conducted under CEU European demographic specifications. (Middle right) Classification rates of simulations conducted under CEU European demographic specifications. (Right) Classification rates of simulations conducted under YRI African demographic specifications. (PDF) [file pgen.1008896.s038.pdf]

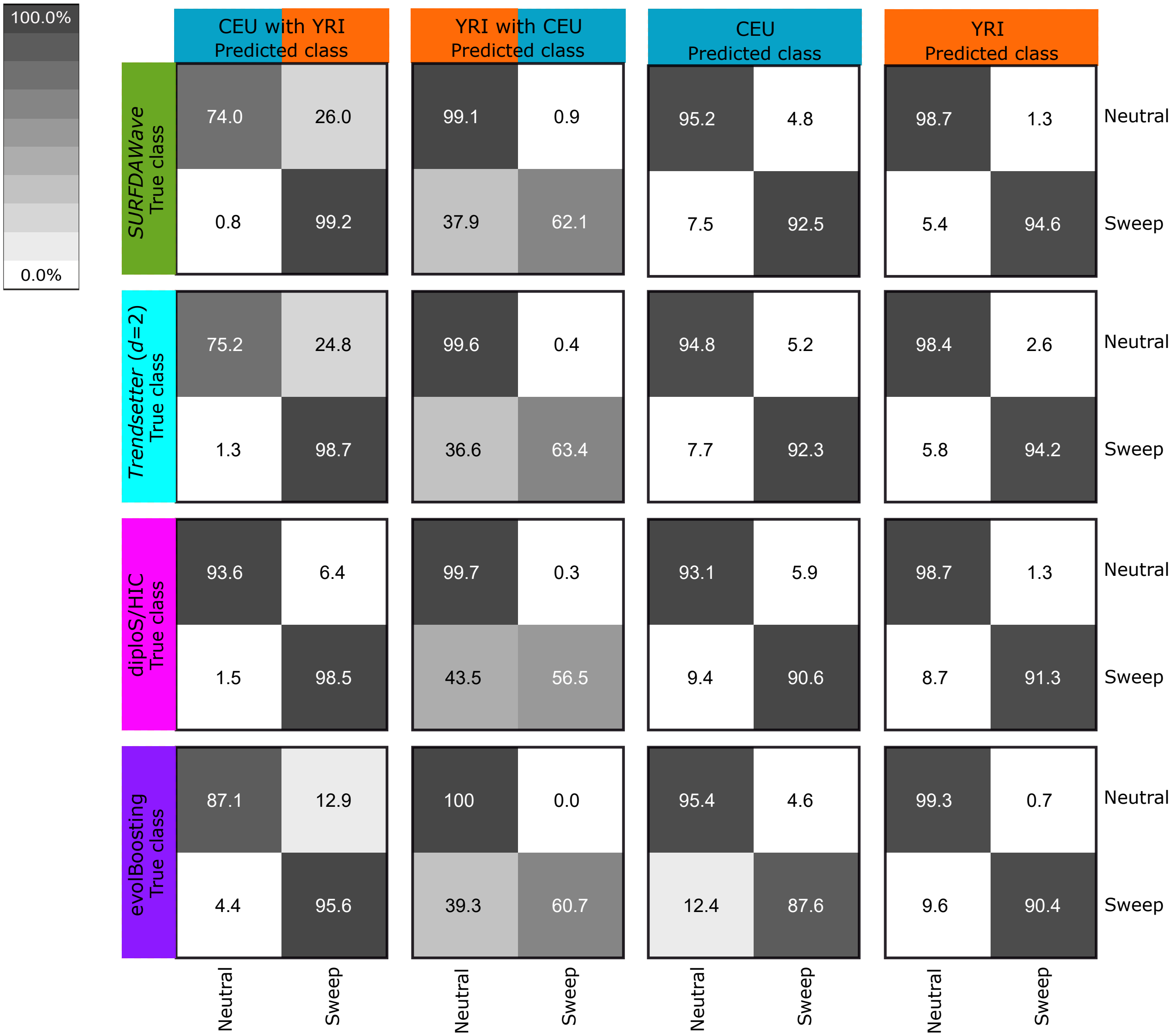

Figure S18: Confusion matrices showing classification results for demographic mis-specification compared to when classifiers are trained with multiple demographic histories rates in *SURFDAWave*, *Trendsetter*, diploS/HIC, and evolBoosting. Summary statistics $\widehat{\pi}$, H_1, H_{12}, H_2/H_1, and frequency of the first, second, third, fourth, and fifth most common haplotypes used by both *Trendsetter* and *SURFDAWave*. *SURFDAWave* results shown are using Daubechies' least-asymmetric wavelets to estimate spatial distributions of summary statistics when level and γ are chosen through cross validation (see *Training the models*). Training data consist of a balanced dataset of simulations conducted under demographic specifications for European (CEU) and African (YRI) human populations when training for multiple demographic histories. (Left) Classification rates of simulations conducted under CEU European demographic specifications when the model is trained with simulations conducted under YRI African demographic specifications. (Middle right) Classification rates of simulations conducted under YRI African demographic specifications when the model is trained with simulations conducted under CEU European demographic specifications. (Middle right) Classification rates of simulations conducted under CEU European demographic specifications. (Right) Classification rates of simulations conducted under YRI African demographic specifications.
