# Supplementary material for: Learning the properties of adaptive regions with functional data analysis
Source: PLoS Genet. 2020 Aug 27;16(8):e1008896. doi: 10.1371/journal.pgen.1008896 (PMC7480868; doi:10.1371/journal.pgen.1008896)
Supplement: S20 Fig — SURFDAWave results shown are using Daubechies’ least-asymmetric wavelets to estimate spatial distributions of summary statistics and γ and levels are chosen through cross validation (see Training the models). Summary statistics π^, H1, H12, H2/H1, and frequency of the first, second, third, fourth, and fifth most common haplotypes used by both Trendsetter and SURFDAWave. (PDF) [file pgen.1008896.s040.pdf]

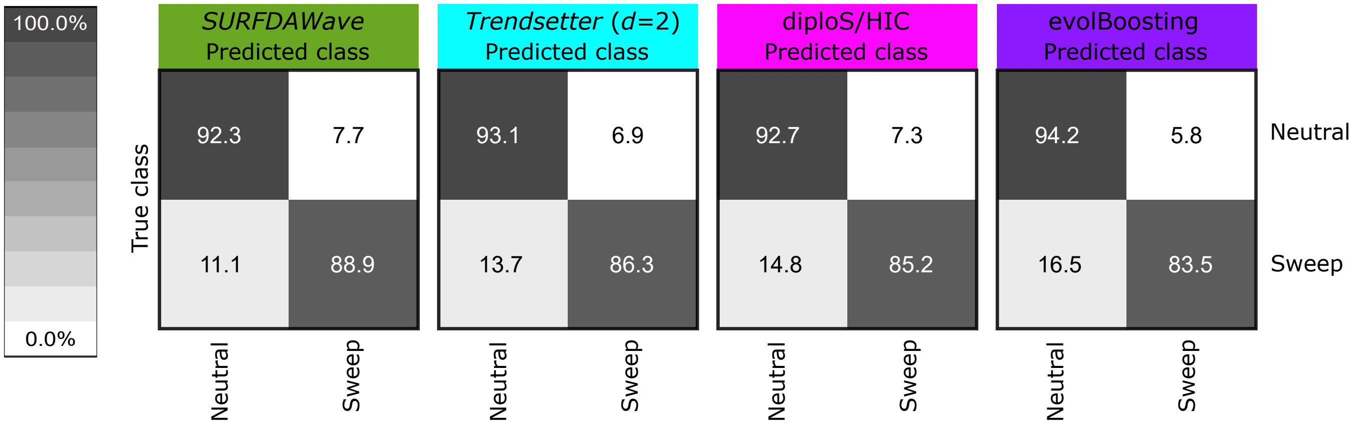

Figure S20: Confusion matrices comparing classification rates when *SURFDAWave*, *Trendsetter*, diploS/HIC, and evolBoosting are trained and tested using simulations conducted under *Drosophila* population parameters to differentiate between sweeps and neutrality. *SURFDAWave* results shown are using Daubechies' least-asymmetric wavelets to estimate spatial distributions of summary statistics and γ and levels are chosen through cross validation (see *Training the models*). Summary statistics $\widehat{\pi}$, H_1, H_{12}, H_2/H_1, and frequency of the first, second, third, fourth, and fifth most common haplotypes used by both *Trendsetter* and *SURFDAWave*.
