# Supplementary material for: Learning the properties of adaptive regions with functional data analysis
Source: PLoS Genet. 2020 Aug 27;16(8):e1008896. doi: 10.1371/journal.pgen.1008896 (PMC7480868; doi:10.1371/journal.pgen.1008896)
Supplement: S21 Fig — All simulations were conducted under European (CEU) demographic history specifications. SURFDAWave results shown are using Daubechies’ least-asymmetric wavelets to estimate spatial distributions of summary statistics and γ and levels are chosen through cross validation (see Training the models). Summary statistics π^, H1, H12, H2/H1, and frequency of the first, second, third, fourth, and fifth most common haplotypes used by both Trendsetter and SURFDAWave. (PDF) [file pgen.1008896.s041.pdf]

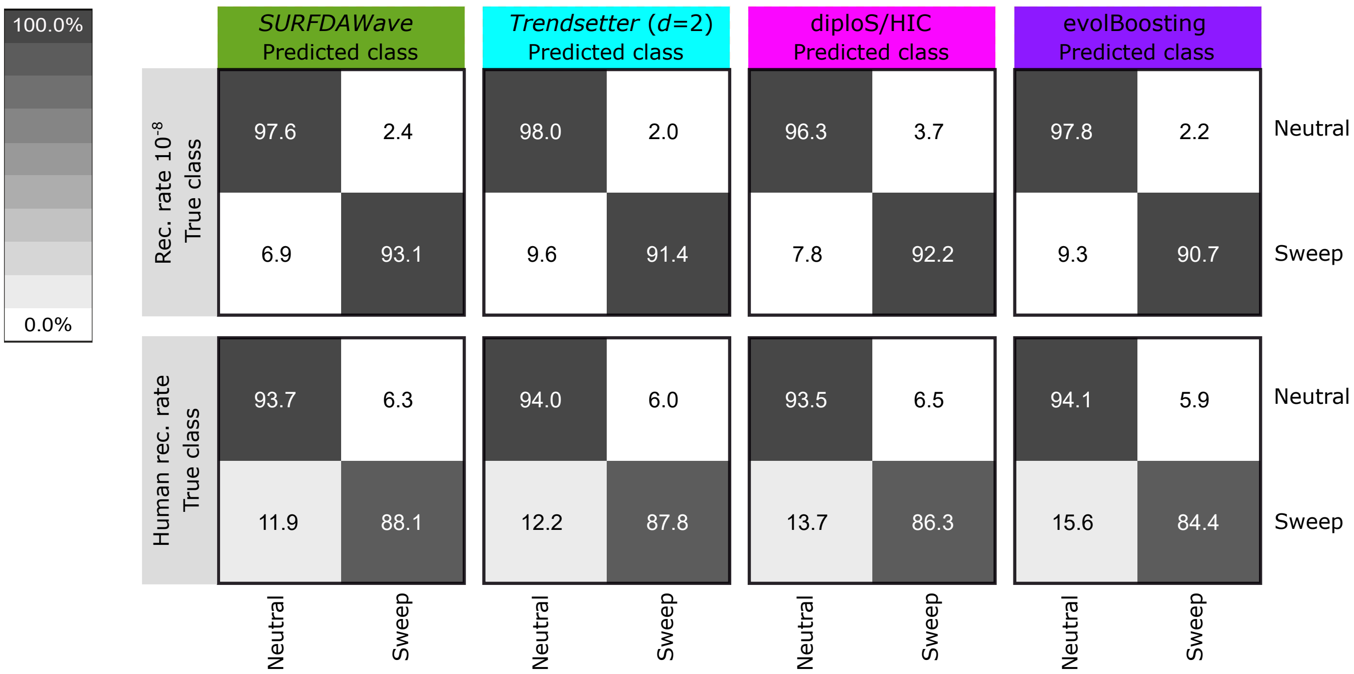

Figure S21: Confusion matrices comparing classification rates of *SURFDAWave*, *Trendsetter*, diploS/HIC, and evolBoosting when applied to simulations with a recombination rate drawn from an exponential distribution with mean 10^{-8} per site per generations, truncated at three times the mean (top row) and recombination rate drawn from a human empirical recombination map (bottom row) to differentiate between sweeps and neutrality. All simulations were conducted under European (CEU) demographic history specifications. *SURFDAWave* results shown are using Daubechies' least-asymmetric wavelets to estimate spatial distributions of summary statistics and γ and levels are chosen through cross validation (see *Training the models*). Summary statistics $\widehat{\pi}$, H_1, H_{12}, H_2/H_1, and frequency of the first, second, third, fourth, and fifth most common haplotypes used by both *Trendsetter* and *SURFDAWave*.
