# Supplementary material for: Learning the properties of adaptive regions with functional data analysis
Source: PLoS Genet. 2020 Aug 27;16(8):e1008896. doi: 10.1371/journal.pgen.1008896 (PMC7480868; doi:10.1371/journal.pgen.1008896)
Supplement: S22 Fig — (Left box plot) Difference in prediction and truth of log scaled time at which mutation became beneficial. (Middle box plot) Difference in prediction and truth of log scaled frequency reached by mutation prior to it becoming beneficial (f). (Right box plot) Difference in prediction and truth of log scaled selection coefficient (s). (PDF) [file pgen.1008896.s042.pdf]

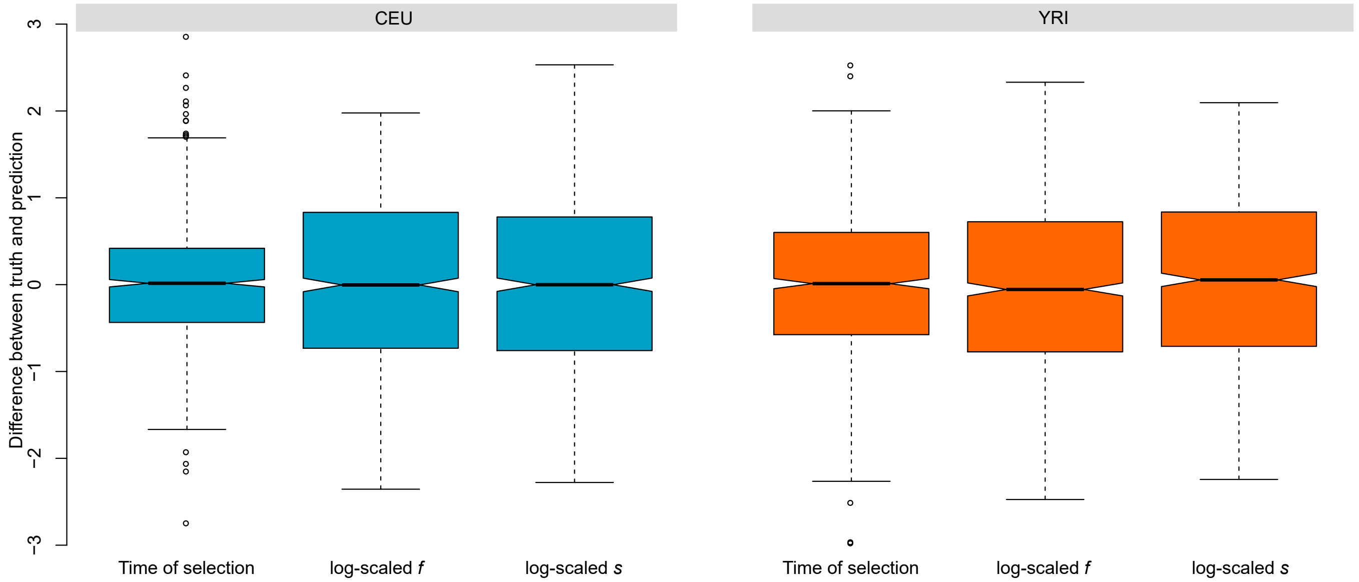


Figure S22: Difference between standardized predicted and actual selection parameters with *SURFDAWave* for the CEU and YRI demographic models. (Left box plot) Difference in prediction and truth of log scaled time at which mutation became beneficial. (Middle box plot) Difference in prediction and truth of log scaled frequency reached by mutation prior to it becoming beneficial ($f$). (Right box plot) Difference in prediction and truth of log scaled selection coefficient ($s$).
