# Supplementary material for: Learning the properties of adaptive regions with functional data analysis
Source: PLoS Genet. 2020 Aug 27;16(8):e1008896. doi: 10.1371/journal.pgen.1008896 (PMC7480868; doi:10.1371/journal.pgen.1008896)
Supplement: S23 Fig — SURFDAWave was trained on simulations of scenarios simulated under demographic specifications for European CEU demographic history. Note that the wavelet reconstructions for all summary statistics are plotted on the same scale, thereby making the distributions of some summaries difficult to decipher as their magnitudes are relatively small. SURFDAWave results shown are using Daubechies’ least-asymmetric wavelets to estimate spatial distributions of summary statistics. Level 1 and γ = 0.6 chosen through cross validation. (PDF) [file pgen.1008896.s043.pdf]

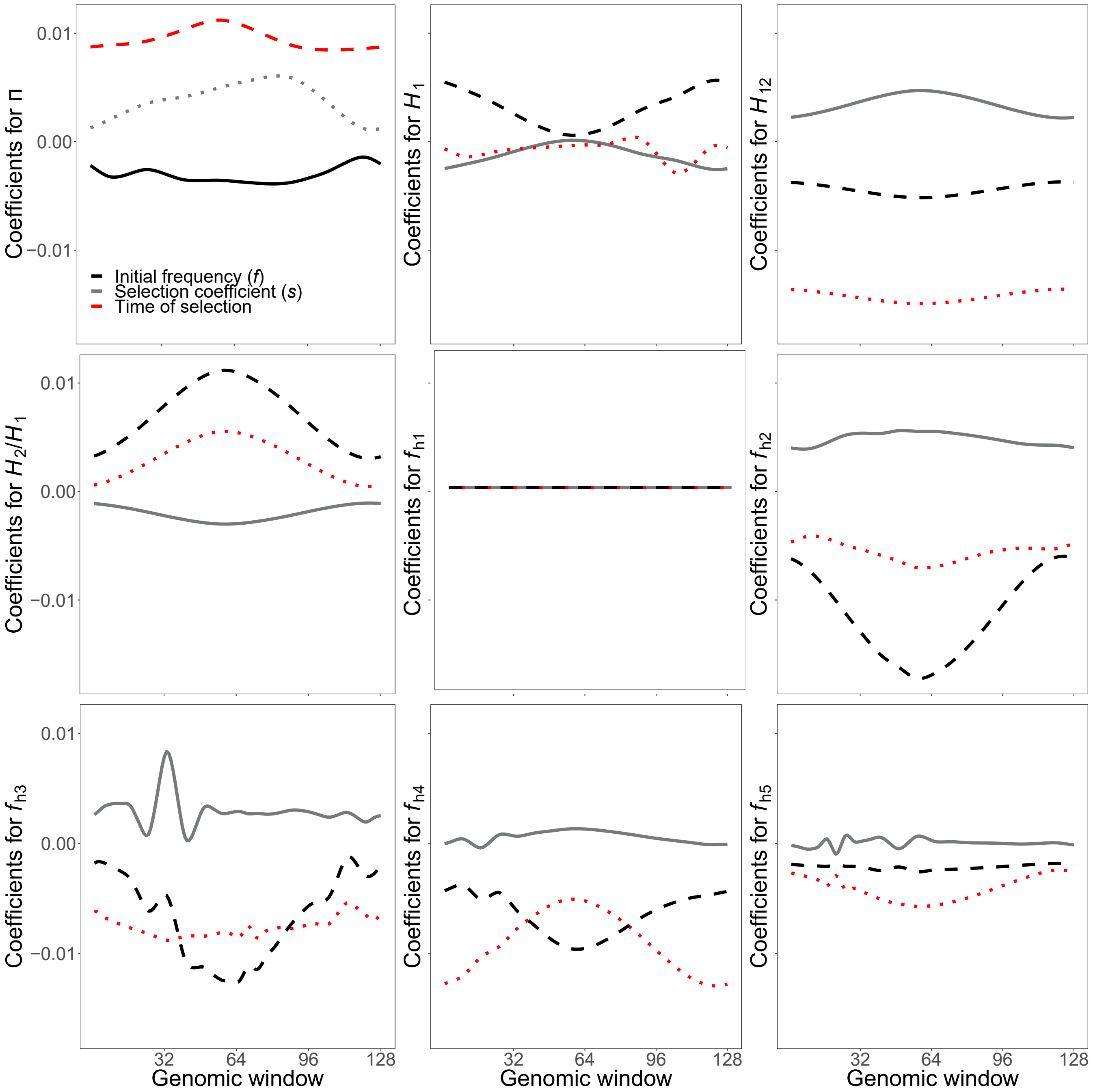

Figure S23: Reconstructed wavelets from regression coefficients (βs) in predicting time at which mutation became beneficial, frequency reached by mutation before becoming beneficial, and selection strength for summary statistics $\widehat{\pi}$, H_1, H_{12}, H_2/H_1, and frequencies of first to fifth most common haplotypes for *SURFDAWave* when $\gamma = 0.6$. *SURFDAWave* was trained on simulations of scenarios simulated under demographic specifications for European CEU demographic history. Note that the wavelet reconstructions for all summary statistics are plotted on the same scale, thereby making the distributions of some summaries difficult to decipher as their magnitudes are relatively small. *SURFDAWave* results shown are using Daubechies' least-asymmetric wavelets to estimate spatial distributions of summary statistics. Level 1 and $\gamma = 0.6$ chosen through cross validation.
