# Supplementary material for: Learning the properties of adaptive regions with functional data analysis
Source: PLoS Genet. 2020 Aug 27;16(8):e1008896. doi: 10.1371/journal.pgen.1008896 (PMC7480868; doi:10.1371/journal.pgen.1008896)
Supplement: S25 Fig — Difference in prediction and truth of time at which mutation became beneficial, difference in prediction and truth of log scaled frequency reached by mutation prior to it becoming beneficial (f), and difference in prediction and truth of log scaled selection coefficient (s) shown as set of three box plots. (Top row) Parameter prediction when training and testing sample sizes are n = 20, 50 or 200 shown for the CEU demographic history. (Row two) Parameter prediction when recombination rate is drawn from an exponential distribution with mean 10−8 per site per generation, truncated at three times the mean or when recombination is drawn from a human empirical recombination map using CEU demographic history. (Row three) Parameter prediction when testing range for initial frequency is f ∈ [0.1, 0.2], which falls outside of training range for CEU and YRI demographic histories. (Bottom row) Parameter prediction when training data is a balanced dataset containing simulations from both CEU and YRI demographic histories and is tested under the specified demographic history. (PDF) [file pgen.1008896.s045.pdf]

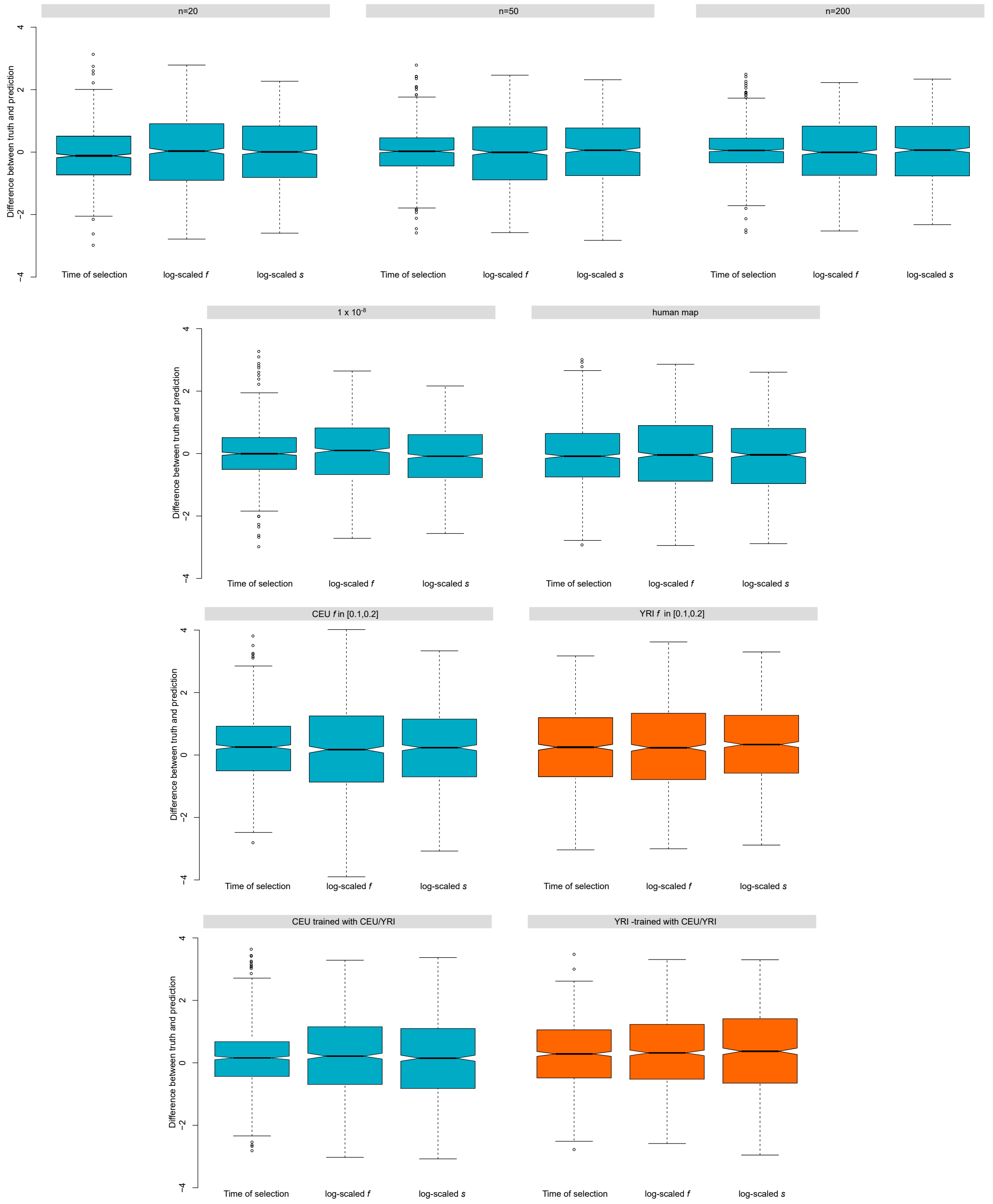

Figure S25: Difference between standardized predicted and actual selection parameters with *SURFDAWave* under several confounding scenarios. Difference in prediction and truth of time at which mutation became beneficial, difference in prediction and truth of log scaled frequency reached by mutation prior to it becoming beneficial (f), and difference in prediction and truth of log scaled selection coefficient (s) shown as set of three box plots. (Top row) Parameter prediction when training and testing sample sizes are $n = 20$, 50 or 200 shown for the CEU demographic history. (Row two) Parameter prediction when recombination rate is drawn from an exponential distribution with mean 10^{-8} per site per generation, truncated at three times the mean or when recombination is drawn from a human empirical recombination map using CEU demographic history. (Row three) Parameter prediction when testing range for initial frequency is $f \in [0.1, 0.2]$, which falls outside of training range for CEU and YRI demographic histories. (Bottom row) Parameter prediction when training data is a balanced dataset containing simulations from both CEU and YRI demographic histories and is tested under the specified demographic history.
