# Supplementary material for: Learning the properties of adaptive regions with functional data analysis
Source: PLoS Genet. 2020 Aug 27;16(8):e1008896. doi: 10.1371/journal.pgen.1008896 (PMC7480868; doi:10.1371/journal.pgen.1008896)
Supplement: S27 Fig — For each classifier we predict the probability of sweep for test 1000 simulations. We divide the predicted probabilities into 950 overlapping windows each of length 0.5, with the first window beginning ranging from 0 to 0.05 and the second from 0.001 to 0.051 and so on with the last window ranging from 0.95 to 1.0. Using these ranges as thresholds, we calculate the mean probability of all predicted probabilities within this range (Mean Prediction) along with the fraction of these cases that are classified as sweep (Observed Fraction). (PDF) [file pgen.1008896.s047.pdf]

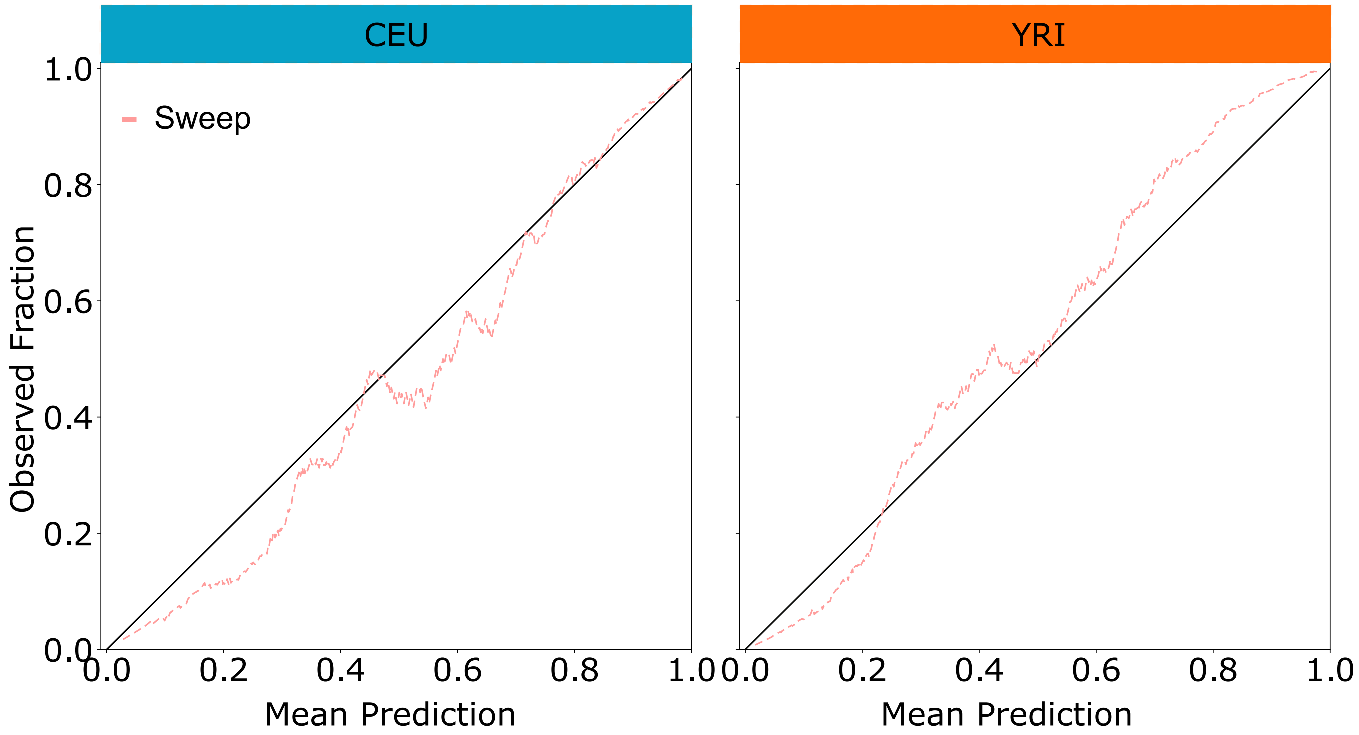

Figure S27: Reliability diagrams showing how close our predicted probabilities are to actual probabilities. For each classifier we predict the probability of sweep for test 1000 simulations. We divide the predicted probabilities into 950 overlapping windows each of length 0.5, with the first window beginning ranging from 0 to 0.05 and the second from 0.001 to 0.051 and so on with the last window ranging from 0.95 to 1.0. Using these ranges as thresholds, we calculate the mean probability of all predicted probabilities within this range (Mean Prediction) along with the fraction of these cases that are classified as sweep (Observed Fraction).
