# Supplementary material for: Learning the properties of adaptive regions with functional data analysis
Source: PLoS Genet. 2020 Aug 27;16(8):e1008896. doi: 10.1371/journal.pgen.1008896 (PMC7480868; doi:10.1371/journal.pgen.1008896)
Supplement: S28 Fig — (Middle) Scatter plot of Selection coefficient versus Time of selection. (Right) Scatter plot of Initial frequency versus selection coefficient. (PDF) [file pgen.1008896.s048.pdf]

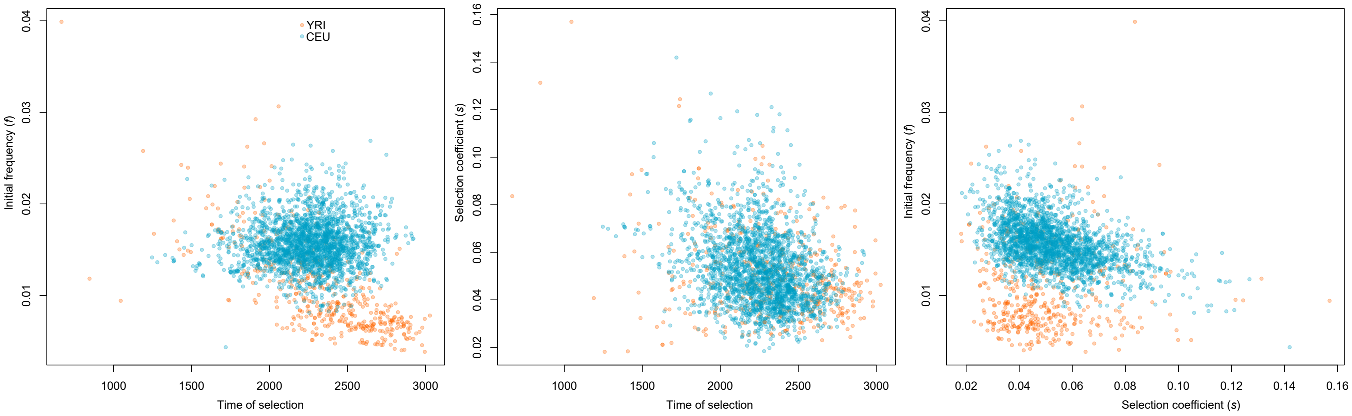

Figure S28: Predicted selection parameters for all genes in YRI and CEU with probability of being classified as sweep greater than 0.7 (Left) Scatter plot of predicted initial frequency mutation reached before becoming beneficial (Initial frequency) versus generations before present at which selection began (Time of selection). (Middle) Scatter plot of Selection coefficient versus Time of selection. (Right) Scatter plot of Initial frequency versus selection coefficient.
