# Supplementary material for: Learning the properties of adaptive regions with functional data analysis
Source: PLoS Genet. 2020 Aug 27;16(8):e1008896. doi: 10.1371/journal.pgen.1008896 (PMC7480868; doi:10.1371/journal.pgen.1008896)
Supplement: S29 Fig — (Middle) Frequency reached by mutation before becoming beneficial (f) as a function of probability of sweep. (Right) Selection coefficient (s) as a function of probability of sweep. (PDF) [file pgen.1008896.s049.pdf]

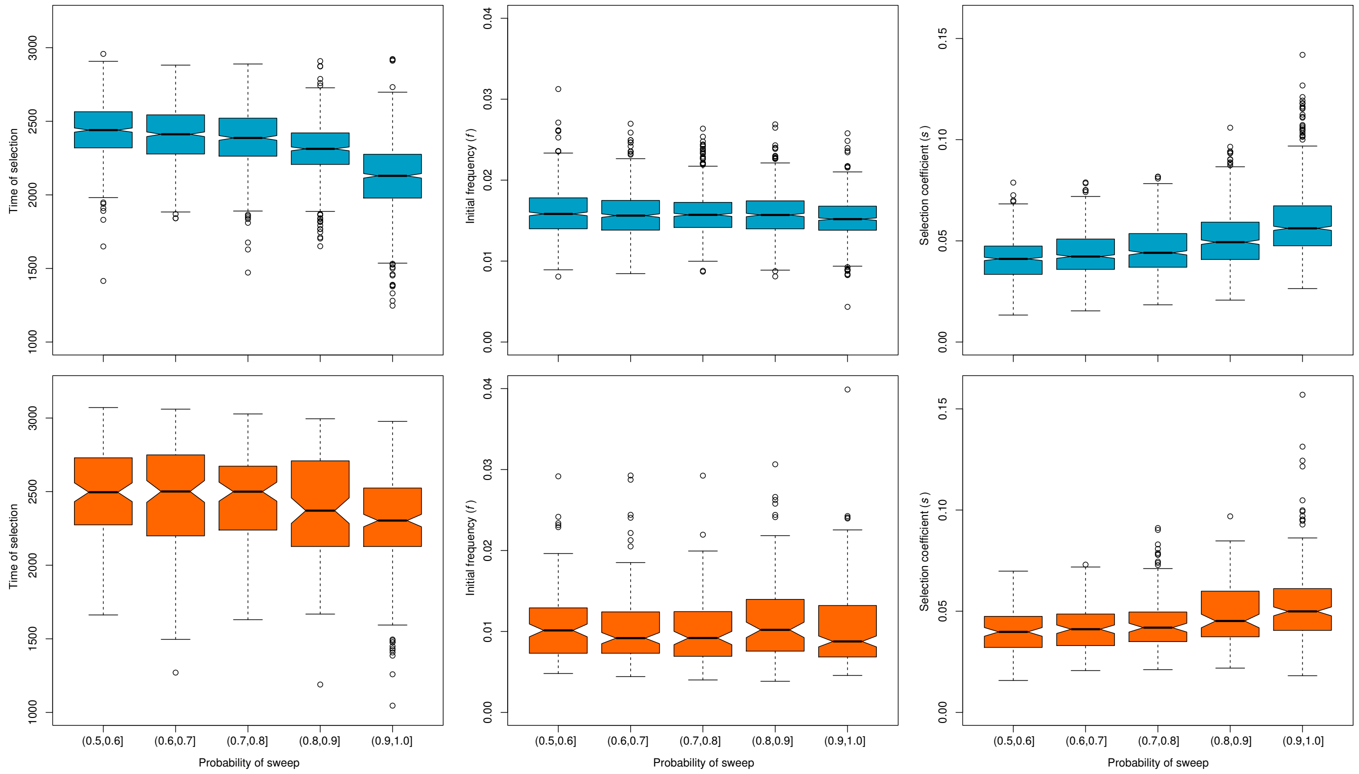

Figure S29: Predicted selection parameters for all genes in YRI (orange) and CEU (blue) with probability of being classified as sweep greater than 0.5 divided into bins of probability of sweep (Left) Predicted number of generations before present at which selection began (Time of selection) as a function of the probability of sweep. (Middle) Frequency reached by mutation before becoming beneficial (f) as a function of probability of sweep. (Right) Selection coefficient (s) as a function of probability of sweep.
