# Supplementary material for: Learning the properties of adaptive regions with functional data analysis
Source: PLoS Genet. 2020 Aug 27;16(8):e1008896. doi: 10.1371/journal.pgen.1008896 (PMC7480868; doi:10.1371/journal.pgen.1008896)
Supplement: S30 Fig — Probability of adaptive introgression across the genomic region of labeled chromosome containing the genes of interest. SURFDAWave is trained to differentiate among selective sweeps, adaptive introgression, and neutrality with simulations conducted under demographic specifications of the CEU demographic history. The black dots show the predicted probability of adaptive introgression and the gray bars show the positions of the labeled genes. Gaps between black dots are the result of filtering low quality genomic regions (see Application of empirical data), such that no SNPs exist in these regions and can therefore not be classified (see S33 Fig as an example of how we classify a SNP spanned by our feature vector). (PDF) [file pgen.1008896.s050.pdf]

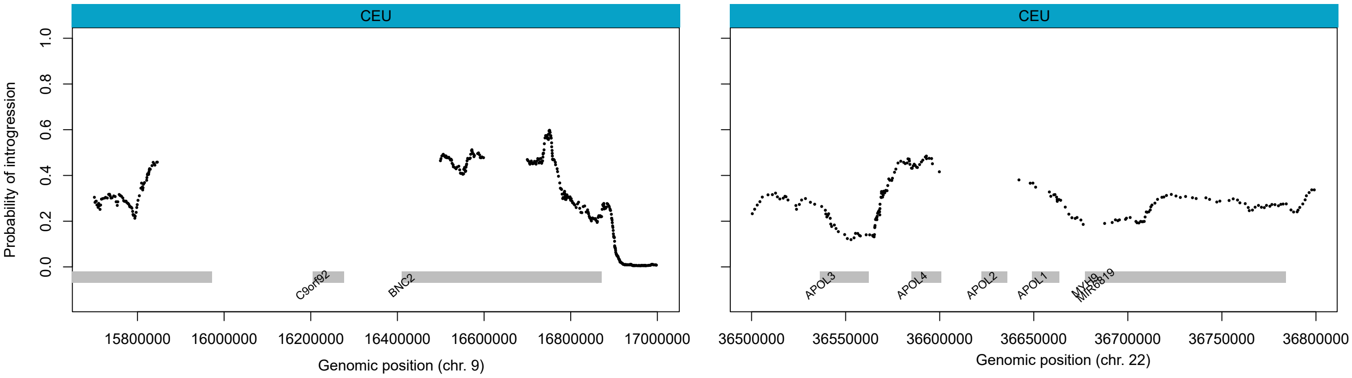

Figure S30: *SURFDAWave* classifier's application to empirical data for CEU to detect adaptive introgression. Probability of adaptive introgression across the genomic region of labeled chromosome containing the genes of interest. *SURFDAWave* is trained to differentiate among selective sweeps, adaptive introgression, and neutrality with simulations conducted under demographic specifications of the CEU demographic history. The black dots show the predicted probability of adaptive introgression and the gray bars show the positions of the labeled genes. Gaps between black dots are the result of filtering low quality genomic regions (see *Application of empirical data*), such that no SNPs exist in these regions and can therefore not be classified (see Figure S33 as an example of how we classify a SNP spanned by our feature vector).
