# Supplementary material for: Learning the properties of adaptive regions with functional data analysis
Source: PLoS Genet. 2020 Aug 27;16(8):e1008896. doi: 10.1371/journal.pgen.1008896 (PMC7480868; doi:10.1371/journal.pgen.1008896)
Supplement: S31 Fig — (PDF) [file pgen.1008896.s051.pdf]

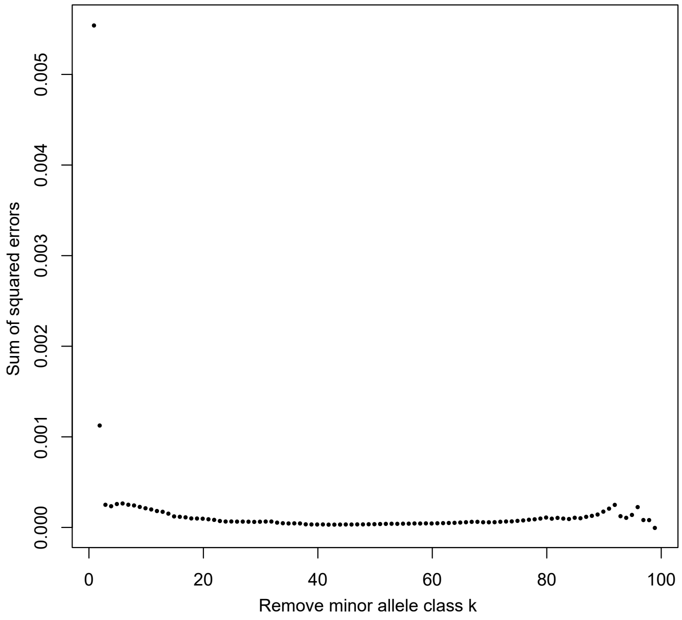

Figure S31: Sum of squared differences between the empirical CEU and the simulated neutral Terhorst normalized minor allele frequency spectra conditional on removing all minor allele classes with k or fewer minor alleles.
