# Supplementary material for: Learning the properties of adaptive regions with functional data analysis
Source: PLoS Genet. 2020 Aug 27;16(8):e1008896. doi: 10.1371/journal.pgen.1008896 (PMC7480868; doi:10.1371/journal.pgen.1008896)
Supplement: S32 Fig — SURFDAWave results shown are using Daubechies’ least-asymmetric wavelets to estimate spatial distributions of summary statistics. Level and gamma chosen through cross validation. (PDF) [file pgen.1008896.s052.pdf]

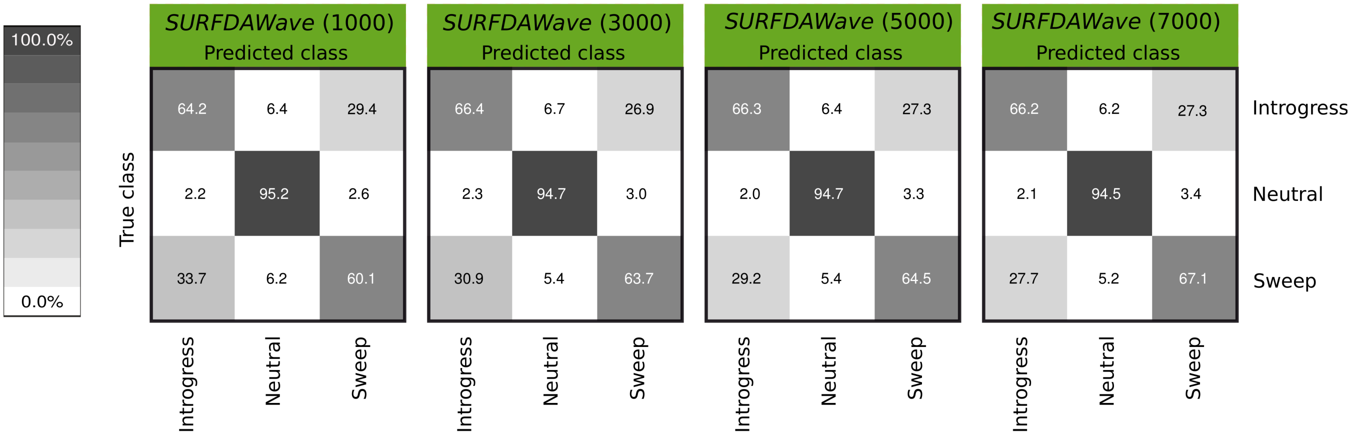

Figure S32: Confusion matrices comparing classification rates of *SURFDAWave* differentiating among adaptive introgression, sweeps, and neutrality when simulated under a constant-size demographic model with non-adaptive introgression with 1000, 3000, 5000, or 7000 training samples per class. *SURFDAWave* results shown are using Daubechies' least-asymmetric wavelets to estimate spatial distributions of summary statistics. Level and *gamma* chosen through cross validation.
