# Supplementary material for: Learning the properties of adaptive regions with functional data analysis
Source: PLoS Genet. 2020 Aug 27;16(8):e1008896. doi: 10.1371/journal.pgen.1008896 (PMC7480868; doi:10.1371/journal.pgen.1008896)
Supplement: S33 Fig — Each bold black line underlines one of the eight 10-SNP long windows. Here we show a sample of six haplotypes (rows) across a string of SNPs (columns) for which we calculate summary statistics in p = 8 windows. Summary statistics are calculated for each 10-SNP window, with windows overlapping with each neighbor for five SNPs. The central SNP is taken to be the putative selected site and is located in the overlap of windows four and five. Here we have underlined the alternating windows used to calculate the two-dimensional statistics in red. (PDF) [file pgen.1008896.s053.pdf]

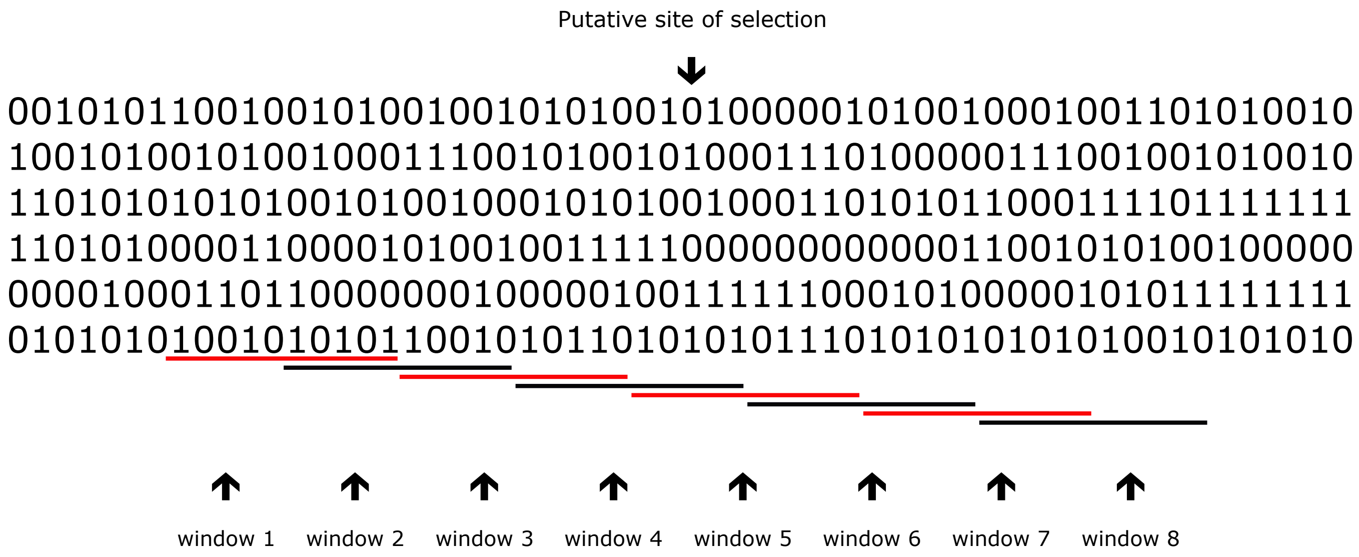

Figure S33: Schematic illustrating windows for which summary statistics are calculated in our implementation of *SURFDAWave*. Each bold black line underlines one of the eight 10-SNP long windows. Here we show a sample of six haplotypes (rows) across a string of SNPs (columns) for which we calculate summary statistics in $p = 8$ windows. Summary statistics are calculated for each 10-SNP window, with windows overlapping with each neighbor for five SNPs. The central SNP is taken to be the putative selected site and is located in the overlap of windows four and five. Here we have underlined the alternating windows used to calculate the two-dimensional statistics in red.
