# Supplementary material for: Learning the properties of adaptive regions with functional data analysis
Source: PLoS Genet. 2020 Aug 27;16(8):e1008896. doi: 10.1371/journal.pgen.1008896 (PMC7480868; doi:10.1371/journal.pgen.1008896)
Supplement: S34 Fig — (Left column) Distribution of time at which tracked mutation becomes beneficial (reaches initial frequency) in simulations of selective sweeps. (Middle column) Distribution of log-scaled initial frequency (input parameter) reached by mutation before becoming beneficial in simulations of selective sweeps. (Right column) Distribution of log-scaled selection coefficient in simulations of selective sweep. (PDF) [file pgen.1008896.s054.pdf]

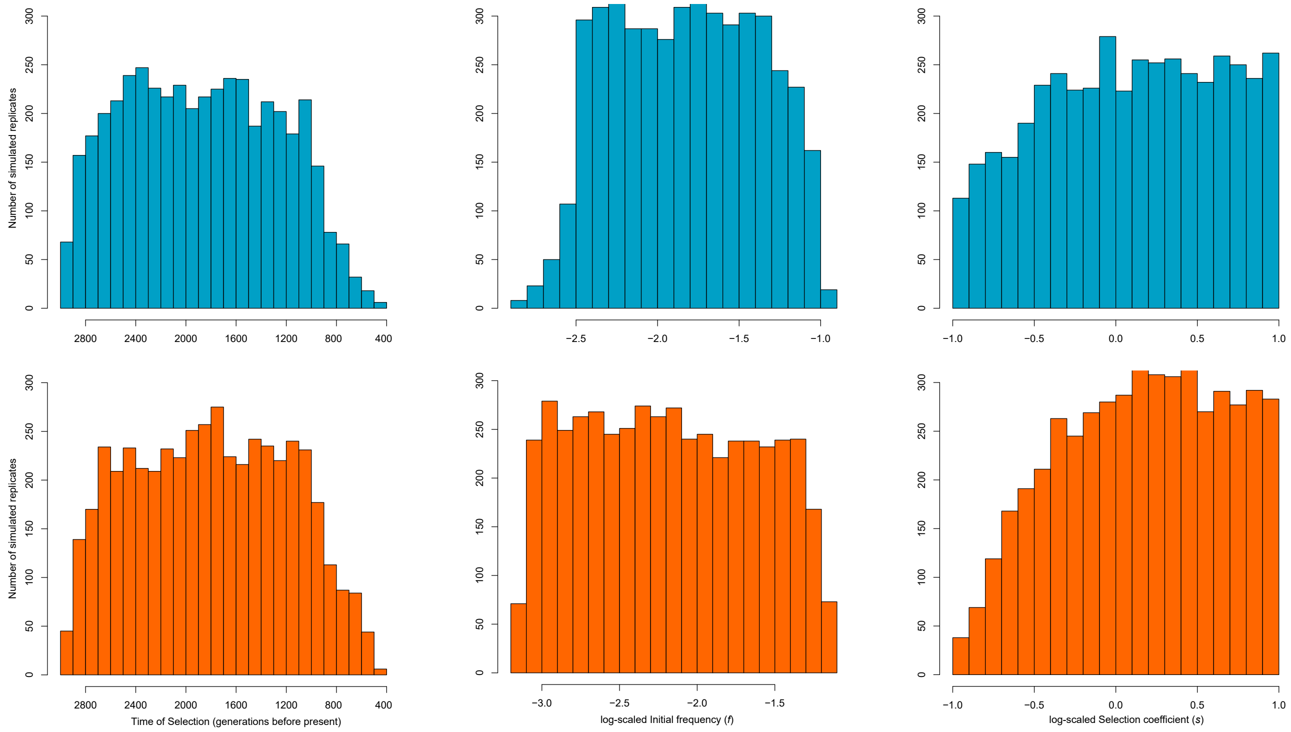

Figure S34: Distribution of selection parameters for simulations of sweeps conducted with demographic history parameters of CEU (top row) and YRI (bottom row). (Left column) Distribution of time at which tracked mutation becomes beneficial (reaches initial frequency) in simulations of selective sweeps. (Middle column) Distribution of log-scaled initial frequency (input parameter) reached by mutation before becoming beneficial in simulations of selective sweeps. (Right column) Distribution of log-scaled selection coefficient in simulations of selective sweep.
